# Seven New Cytotoxic and Antimicrobial Xanthoquinodins from *Jugulospora vestita*

**DOI:** 10.3390/jof6040188

**Published:** 2020-09-25

**Authors:** Lulu Shao, Yasmina Marin-Felix, Frank Surup, Alberto M. Stchigel, Marc Stadler

**Affiliations:** 1 Department Microbial Drugs, Helmholtz Centre for Infection Research, Inhoffenstrasse 7, 38124 Braunschweig, Germany; Lulu.Shao@helmholtz-hzi.de (L.S.); Frank.Surup@helmholtz-hzi.de (F.S.); 2 South China Botanical Garden, Chinese Academy of Sciences, Xingke Road 723, Tianhe District, Guangzhou 510650, China; 3 School of Life Sciences, University of Chinese Academy of Sciences, Yuquanlu 19A, Beijing 100049, China; 4 Mycology Unit, Medical School and IISPV, Universitat Rovira i Virgili, C/Sant Llorenç 21, 43201 Reus, Tarragona, Spain; albertomiguel.stchigel@urv.cat

**Keywords:** antimicrobial activity, cytotoxicity, secondary metabolites, Sordariales, xanthoquinodins

## Abstract

During the course of a screening for novel biologically active secondary metabolites produced by the Sordariomycetes (Ascomycota, Fungi), the ex-type strain of *Jugulospora vestita* was found to produce seven novel xanthone-anthraquinone heterodimers, xanthoquinodin A11 (**1**) and xanthoquinodins B10–15 (**2**–**7**), together with the already known compound xanthoquinodin B4 (**8**). The structures of the xanthoquinodins were determined by analysis of the nuclear magnetic resonance (NMR) spectroscopic and mass spectrometric data. Moreover, the absolute configurations of these metabolites were established by analysis of the ^1^H−^1^H coupling constants, nuclear Overhauser effect spectroscopy (NOESY) correlations, and Electronic Circular Dichroism (ECD) spectroscopic data. Antifungal and antibacterial activities as well as cytotoxicity of all compounds were tested. Xanthoquinodin B11 showed fungicidal activities against *Mucor hiemalis* [minimum inhibitory concentration (MIC) 2.1 µg/mL], *Rhodotorula glutinis* (MIC 2.1 µg/mL), and *Pichia anomala* (MIC 8.3 µg/mL). All the compounds **1**–**8** displayed anti-Gram-positive bacteria activity (MIC 0.2–8.3 µg/mL). In addition, all these eight compounds showed cytotoxicity against KB 3.1, L929, A549, SK-OV-3, PC-3, A431, and MCF-7 mammalian cell lines. The six novel compounds (**1**–**3**, **5**–**7**), together with xanthoquinodin B4, were also found in the screening of other strains belonging to *Jugulospora rotula*, revealing the potential chemotaxonomic significance of the compound class for the genus.

## 1. Introduction

Nowadays, the increasing drug resistance by bacterial and fungal pathogens and the decrease of new therapeutic agents and developmental candidates are a global hurdle [1]. This problem has led to strong demand to increase the chemical diversity of antibiotics and antifungals, with the fungi, whose secondary metabolites remain poorly studied, being the potential solution for this challenge [2]. Fungal secondary metabolites can also be employed as new and beneficial therapeutic agents, such as the cyathane diterpenoids found in the genus *Hericium* (Basidiomycota), which can be used to treat neurodegenerative diseases [3]. Another example is the cytotoxic compounds, which can hold a great potential for the cancer treatment since these can be combined with targeted therapy, achieving the delivery of the drug to cancer cell-specific genes or proteins or to tissue microenvironment of developing cancer [4]. Even though most anticancer drugs are retrieved from plants and bacteria [2], some natural substances from fungi are currently in the preclinical and clinical development stage, such as irofulven, which has been evaluated in phase I and II, showing promising results against brain and central nervous system, breast, colon, lung, ovarian, pancreas, and prostate cancers, as well as leukemia and sarcoma [5,6,7].

During the course of an ongoing project, rare and interesting members of the Sordariomycetes are being tested for the production of novel biologically active secondary metabolites, since this group of fungi has already been demonstrated to include prolific producer taxa [8,9,10]. According to Bills and Gloer [11], many important metabolites with practical potential have been discovered from the Sordariomycetes. Prominent examples are antibacterial antibiotics (cephalosporins and fusidic acid), the immunomodulatory drug cyclosporine, the ergot alkaloids, the anthelmintic cyclodepsipeptide PF1022A, and the serine palmitoyltransferase inhibitor myriocin that gave rise to the anti-inflammatory drug fingolimod. In addition, the potent antiparasitic nodulisporic acid, as well as the antimycotic sordarins, have also been found from species of Sordariomycetes [10,12].

*Jugulospora vestita* was initially described by Udagawa and Horie [13] as *Apiosordaria vestita* to accommodate a soil fungus isolated from Nepal. This species is characterized by ostiolate ascomata and two-celled ascospores, with pitted upper cells tending to appear reticulate [13]. In a recent phylogenetic study based on sequences of the internal transcribed spacer region (ITS), the nuclear rDNA large subunit (LSU), and fragments of ribosomal polymerase II subunit 2 (*rpb2*) and β-tubulin (*tub2*) genes, the type strain of this taxon was located far from the type species of the genus *Apiosordaria*, *A. verruculosa*, being placed in the monophyletic clade of the genus *Jugulospora* [14]. Therefore, the new combination *J. vestita* was proposed. The screening for novel biologically active secondary metabolites of the ex-type strain of this species led to the isolation of seven previously undescribed xanthoquinodins, together with the already known xanthoquinodin B4. Their structures were elucidated by one-dimensional and two-dimensional nuclear magnetic resonance (1D- and 2D-NMR) spectroscopy, and Electronic Circular Dichroism (ECD) spectra. Details of the isolation, structure elucidation, antimicrobial activity, and cytotoxicity of these new xanthoquinodins are presented herein.

## 2. Materials and Methods

### 2.1. General

Ultraviolet-Visible (UV/Vis) spectra were acquired using a UV-Vis spectrophotometer UV-2450 (Shimadzu, Kyoto, Japan). Optical rotations were recorded in methanol (MeOH) solution on a MCP 150 polarimeter at 20 °C (Anton-Paar Opto Tec GmbH, Seelze, Germany). ECD spectra were obtained on a J-815 spectropolarimeter (JASCO, Pfungstadt, Germany). High-resolution electrospray ionization mass spectra (HR-ESI-MS) were acquired with an Agilent 1200 Infinity Series HPLC-UV system (Agilent Technologies, Santa Clara, CA, USA) utilizing a C_18_ Acquity UPLC BEH column (2.1 × 50 mm, 1.7 µm: Waters, Milford, MA, USA), solvent A: H_2_O + 0.1% formic acid; solvent B: acetonitrile (ACN) + 0.1% formic acid, gradient: 5% B for 0.5 min increasing to 100% B in 19.5 min, maintaining 100% B for 5 min, flow rate 0.6 mL min^−1^, UV/Vis detection 190–600 nm) connected to an time-of-flight mass spectrometer (ESI-TOF-MS, Maxis, Bruker, Billerica, MA, USA) (scan range 100–2500 *m/z*, rate 2 Hz, capillary voltage 4500 V, dry temperature 200 °C). NMR spectra were recorded with an Avance III 500 spectrometer (Bruker, Billerica, MA, USA, ^1^H-NMR: 500 MHz, and ^13^C-NMR: 125 MHz).

### 2.2. Fermentation, Extraction, and Isolation

The ex-type strain of *Jugulospora vestita* CBS 135.91, which was isolated from soil from Nepal (https://wi.knaw.nl/page/fungal_table), was grown on potato dextrose agar (PDA; HiMedia, Mumbai, India) plates for 7 days at 23 °C; then, the fungal colonies on the culture medium were cut into pieces (1 × 1 cm) and transferred into two 250 mL Erlenmeyer flasks, each containing 100 mL of yeast-malt extract broth (YM broth; 4 g/L yeast extract, 10 g/L malt extract, 4 g/L d-glucose and pH 6.3 [3]). The seed fungus was incubated for 5 days at 23 °C under shake condition at 140 rpm. Fermentation was carried out in 20 × 1 L Erlenmeyer flasks, each containing 400 mL of YM broth and inoculated with 5.0 mL of the mycelial suspension and cultivated for 13 days at 26 °C on a rotary shaker at 108 rpm.

The mycelium and the supernatant were separated by filtration via gauze. The mycelium was macerated three times by acetone and put in an ultrasonic water bath for 30 min at 40 °C. The supernatant was mixed with 275 g of adsorbent resin (Amberlite XAD-16 N, Sigma-Aldrich, Deisenhofen, Germany) and stirred for 2 h. The Amberlite^®^ resin was then filtered and eluted three times with acetone. The resulting acetone extracts were dried in vacuo at 40 °C and the remaining aqueous residue was diluted with the same amounts of ethyl acetate (EtOAc) and extracted three times. The mycelium and the supernatant extracts were combined according to their chromatographic homogeneity to afford 738 mg of an oily crude extract.

The total extract was dissolved in MeOH and subjected to preparative reverse phase HPLC (PLC 2020, Gilson, Middleton, WI, USA). As stationary phase, VP Nucleodur 100-5 C18 ec column (250 × 40 mm, 7 µm, Macherey-Nagel, Düren, Germany) was used, while the mobile phase consisted of: solvent A, deionized water; solvent B, ACN. Purification of the crude extract was performed by using a linear gradient elution of 35–80% aqueous ACN with 0.05% formic acid at a flow rate of 45 mL/min for 55 min, 80–100% solvent B in 5 min, and finally, isocratic elution at 100% solvent B for 5 min to afford compounds **2** (*t*_R_: 13.4–13.5 min, 34 mg), **3** (*t*_R_: 13.2–13.3 min, 13 mg), and other observed peaks (F1–F8). Compounds **8** (*t*_R_: 10.6–10.8 min, 18.5 mg) and **7** (*t*_R_: 11.4–11.5 min, 5.5 mg) were obtained from purification of fraction F4 with the elution gradient 65–75% solvent B for 23 min, followed by isocratic elution with 100% B for 10 min. With the same method as for fraction F4, compounds **4** (*t*_R_: 11.6–11.7 min, 9.5 mg), **6** (*t*_R_: 12.5–12.6 min, 2 mg), and **5** (*t*_R_: 12.2–12.3 min, 3.3 mg) were obtained from fraction F5 and F6, as well as **1** (*t*_R_: 13.5–13.6 min, 7.5 mg) from fraction F8.

For comparison of secondary metabolite production of strains of *Jugulospora*, *Jugulospora vestita* strain CBS 135.91 and *Jugulospora rotula* strains CBS 110112, CBS 110113, FMR 12691, and FMR 12781, as well as *Triangularia backusii* FMR 12439 (a species that was previously also placed in the genus *Jugulospora*), were grown on PDA at 23 °C and the well-grown cultures were cut into small pieces using a cork borer (7 mm). Subsequently, a 200 mL Erlenmeyer flask containing 100 mL of YM were inoculated using five of those pieces and incubated at 23 °C on a rotary shaker (140 rpm). The growth of the fungus was monitored by constantly checking the amount of free glucose using Medi-test Glucose (Macherey-Nagel, Düren, Germany), and the fermentation was terminated 3 days after glucose depletion. Then, the mycelium and the supernatant were separated by filtration via gauze. The mycelia were extracted one time with acetone in an ultrasonic bath at 40 °C for 30 min. The resulting acetone extracts were dried in vacuo at 40 °C. The remaining aqueous residues were diluted with the same amounts of ethyl acetate and extracted one time. The supernatants were extracted with the same amount of EtOAc. The solvents were dried in vacuo at 40 °C.

### 2.3. Spectral Data

#### 2.3.1. Xanthoquinodin A11 (**1**)

Yellow, powder; [*α*]^20^_D_ + 491° (*c* 0.005, MeOH); UV (MeOH) *λ*_max_ (log *ɛ*) 199 (4.6), 274 (3.8), 345 (4.4); CD (*c* 1.6 × 10^–3^ M, MeOH) λ_max_ (Δ*ɛ*) 228 (+14.90), 271 (+3.15), 326 (+28.27), 378 (+4.99); ^1^H-NMR and ^13^C-NMR see Table 1; ESI-MS: *m/z* 643.20 (M − H)^−^ and 645.26 (M + H)^+^; high resolution electrospray ionisation mass spectrometry (HRESIMS) *m/z* 645.1965 (M + H)^+^ (calculated for C_35_H_33_O_12_, 645.1967).

#### 2.3.2. Xanthoquinodin B10 (**2**)

Yellow, amorphous solid; [α]^20^_D_ + 474° (*c* 0.005, MeOH); UV (MeOH) *λ*_max_ (log *ɛ*) 199 (4.5), 274 (3.8), 353 (4.3); CD (*c* 1.6 × 10^–3^ M, MeOH) *λ*_max_ (Δ*ɛ*) 228 (+15.91), 264 (+2.69), 320 (+15.51), 359 (+14.38); ^1^H-NMR and ^13^C-NMR see Table 1; ESI-MS: *m/z* 643.19 (M − H)^−^ and 645.24 (M + H)^+^; HRESIMS *m/z* 645.1967 (M + H)^+^ (calculated for C_35_H_33_O_12_, 645.1967).

#### 2.3.3. Xanthoquinodin B11 (**3**)

Yellow, amorphous solid; [α]^20^_D_ + 438° (*c* 0.005, MeOH); UV (MeOH) *λ*_max_ (log *ɛ*) 198 (4.5), 274 (3.6), 354 (4.1); CD (*c* 1.6 × 10^–3^ M, MeOH) *λ*_max_ (Δ*ɛ*) 234 (–22.84), 263 (+2.78), 325 (+33.59), 353 (+19.33); ^1^H-NMR and ^13^C-NMR see Table 1; ESI-MS: *m/z* 643.19 (M − H)^−^ and 645.24 (M + H)^+^; HRESIMS *m/z* 645.1966 (M + H)^+^ (calculated for C_35_H_33_O_12_, 645.1967).

#### 2.3.4. Xanthoquinodin B12 (**4**)

Yellow, crystalline solid; [α]^20^_D_ + 442° (*c* 0.004, MeOH); UV (MeOH) *λ*_max_ (log *ɛ*) 199 (4.5), 275 (3.8), 355 (4.2); CD (*c* 1.5 × 10^–3^ M, MeOH) *λ*_max_ (Δ*ɛ*) 228 (+1.85), 264 (+2.74), 324 (+22.52), 359 (+12.43); ^1^H-NMR and ^13^C-NMR see Table 1; ESI-MS: *m/z* 659.20 (M − H)^−^ and 661.24 (M + H)^+^; HRESIMS *m/z* 661.1915 (M + H)^+^ (calculated for C_35_H_34_O_13_, 661.1916).

#### 2.3.5. Xanthoquinodin B13 (**5**)

Yellow, amorphous solid; [α]^20^_D_ + 489° (*c* 0.001, MeOH); UV (MeOH) *λ*_max_ (log *ɛ*) 199 (4.6), 274(3.9), 354 (4.3); CD (*c* 1.6 × 10^–3^ M, MeOH) *λ*_max_ (Δ*ɛ*) 228 (+12.74), 264 (+2.90), 323 (+15.04), 360 (+13.37); ^1^H-NMR and ^13^C-NMR see Table 2; ESI-MS: *m/z* 615.18 (M − H) ^−^ and 617.19 (M + H)^+^; HRESIMS *m/z* 617.1650 (M + H)^+^ (calculated for C_33_H_29_O_12_, 617.1654).

#### 2.3.6. Xanthoquinodin B14 (**6**)

Yellow, amorphous solid; [α]^20^_D_ + 508° (*c* 0.002, MeOH); UV (MeOH) *λ*_max_ (log *ɛ*) 199 (4.5), 275 (4.0), 361 (4.1); CD (*c* 1.6 × 10^–3^ M, MeOH) *λ*_max_ (Δ*ɛ*) 233 (–12.96), 264 (+8.84), 323 (+26.05); ^1^H-NMR and ^13^C-NMR see Table 2; ESI-MS: *m/z* 643.20 (M − H)^−^ and 645.24 (M + H)^+^; HRESIMS *m/z* 645.1967 (M + H)^+^ (calculated for C_35_H_33_O_12_, 645.1967).

#### 2.3.7. Xanthoquinodin B15 (**7**)

Yellow, amorphous solid; [α]^20^_D_ + 425° (*c* 0.004, MeOH); UV (MeOH) *λ*_max_ (log *ɛ*) 199 (4.4), 207 (4.3), 276 (4.0), 361 (4.1); CD (*c* 1.5 × 10^–3^ M, MeOH) *λ*_max_ (Δ*ɛ*) 207 (–35.18), 231 (–17.20), 264 (+10.67), 323 (+37.35); ^1^H-NMR and ^13^C-NMR see Table 2; ESI-MS: *m/z* 661.19 (M − H)^−^ and 663.27 (M + H)^+^; HRESIMS *m/z* 663.2072 (M + H)^+^ (calculated for C_35_H_35_O_13_, 663.2072).

#### 2.3.8. Xanthoquinodin B4 (**8**)

Yellow, amorphous solid; [α]^20^_D_ + 540.2° (*c* 0.005, MeOH); UV (MeOH) *λ*_max_ (log *ɛ*) 202 (4.4), 274 (3.9), 352 (4.3); CD (*c* 1.5 × 10^–3^ M, MeOH) *λ*_max_ (Δ*ɛ*) 227 (+15.00), 263 (+1.74), 324 (+14.12), 357 (+14.60); ^1^H-NMR (500 MHz, CDCl_3_): δ_H_ 2.11 (1H, m, H-4a), 2.24 (1H, m, H-4b), 2.39 (3H, s, H-16′), 2.60 (1H, d, *J* = 18.0 Hz, H-15′b), 2.68 (2H, m, H-5), 2.73 (1H, d, *J* = 18.0 Hz, H-15′a), 3.73 (3H, s, 15-OCH_3_), 4.4 (1H, dd, *J* = 12.5, 5.0 Hz, H-3), 4.55 (1H, s, H-1′), 4.83 (1H, d, *J* = 6.0 Hz, H-11′), 6.10 (1H, s, H-11), 6.47 (1H, d, *J* = 8.5 Hz, H-13′), 6.51 (1H, dd, *J* = 8.5, 6.5 Hz, H-12′), 6.78 (1H, s, H-5′), 6.80 (1H, s, H-3′), 11.05 (1H, s, 10-OH), 11:28 (1H, s, 6′-OH), 13.95 (1H, s, 6-OH);^13^C NMR (125 MHz, CDCl_3_): δ_C_ 22.0 (CH_3_, 16′-CH_3_), 23.8 (CH_2_, CH_2_-4), 27.7 (CH_2_, CH_2_-5), 35.3 (CH_2_, CH_2_-15′), 39.0 (CH, CH_2_-11′), 42.8 (C, C-14′), 53.2 (CH_3_, 15-OCH_3_), 71.6 (CH, CH_2_-3), 73.5 (CH, CH-1′), 85.3 (C, C-2), 101.6 (C, C-7), 105.0 (C, C-9), 105.3 (C, C-14′), 111.1 (C, C-7′), 114.1 (CH, CH-11), 115.4 (C, C-13), 119.0 (CH, CH-5′), 122.1 (CH, CH-3′), 131.9 (CH, CH-12′), 132.9 (CH, CH-13′), 140.6 (C, C-2′), 147.9 (C, C-4′), 148.0 (C, C-12), 160.0 (C, C-10), 161.7 (C, C-6′), 169.9 (C, C-15), 178.5 (C, C-6), 183.9 (C, C-8′), 186.7 (C, C-8), 188.2 (C, C-10′); ESI-MS: *m/z* 573.15 (M − H) ^−^ and 575.21 (M + H)^+^; HRESIMS *m/z* 575.1547 (M + H)^+^ (calculated for C_31_H_27_O_11_, 575.1548).

### 2.4. Biological Assays

Compounds were tested for their antimicrobial activity against four fungi (*Candida albicans*, *Mucor hiemails*, *Pichia anomala*, *Rhodotorula glutinis* and *Schizosaccharomyces pombe*), four different Gram-positive bacteria (*Bacillus subtilis*, *Micrococcus luteus*, *Mycobacterium smegmatis* and *Staphylococcus aureus*), and three Gram-negative bacteria (*Chromobacterium violaceum*, *Escherichia coli* and *Pseudomonas aeruginosa*), using oxytetracycline as a positive control against Gram-positive and Gram-negative bacteria, while nystatin was used as an antifungal positive control. Besides, cytotoxicities of the compounds against seven mammalian cell lines (human endocervical adenocarcinoma KB 3.1, breast cancer MCF-7, lung cancer A549, ovary cancer SK-OV-3, prostate cancer PC-3, squamous cancer A431, and mouse fibroblasts L929) were determined by the microculture tetrazolium test (MTT) method, using epothilon B as the positive control. Both bioactivity assays were performed following our standard protocols [15].

### 2.5. Phylogenetic Study

The phylogenetic analysis was carried out based on the combination of the ITS, LSU, *rpb2*, and *tub2* sequences of the strain of *Jugulospora vestita* studied here and selected members belonging to the Sordariales, with *Camarops amorpha* SMH 1450 as outgroup. Each locus was aligned separately using MAFFT v. 7 [16], manually adjusted in MEGA v. 6.06 [17], and the individual gene phylogenies were checked for conflicts before the four gene datasets were concatenated [18,19]. The Maximum-Likelihood (ML) and Bayesian Inference (BI) methods were used in a phylogenetic analysis including the four loci concatenated as described by Hernández-Restrepo et al. [20]. Bootstrap support (bs) ≥ 70 and posterior probability values (pp) ≥ 0.95 were considered significant [21]. The alignment used in the phylogenetic analysis was deposited in TreeBASE (S26889).

## 3. Results and Discussion

### 3.1. Structure Elucidation of Compounds ***1***–***7***

In total, seven novel compounds (**1**−**7**) and xanthoquinodin B4 (**8**) [22], were isolated from the ex-type strain of *Jugulospora vestita* (Figure 1). Their structures were elucidated by 1D- and 2D-NMR spectroscopy (Supplementary Figures S1–S6, S8–S13, S15–S20, S22–S27, S29–S34, S36–S41, and S43–S48), HR-MS (Supplementary Figures S7, S14, S21, S28, S35, S42, and S49), and ECD spectra.

Compound **1** was obtained as a yellow powder and its molecular formula was established as C_35_H_32_O_12_ (20 degrees of unsaturation) according to the mass ion peak at *m*/*z* 645.1967 [M + H]^+^ in the HRESIMS spectrum. The ^1^H- and ^13^C-NMR spectra (Table 1), accompanied with heteronuclear single quantum coherence (HSQC) correlations, revealed signals of two methyl (δ_C_ 22.0, 13.5), one methoxy (δ_C_ 53.3), five sp^3^ methylenes (δ_C_ 36.2, 35.1, 27.5, 23.7, and 18.4), three sp^3^ methines (δ_C_ 72.8, 71.8, and 37.3), five aromatic methines (δ_C_ 132.4, 131.9, 123.1, 119.3, and 110.9), seventeen sp^2^ quaternary carbons, and two sp^3^ quaternary carbons (δ_C_ 41.5 and 84.3 (oxygenated)). In the ^1^H–^1^H correlation spectroscopy (^1^H–^1^H COSY) spectrum, there were three isolated spin systems (H-3–H-4–H-5, H-11′–H-12′–H-13′, and H-19′–H-20′–H-21′). The heteronuclear multiple bond correlation (HMBC) spectrum showed correlations from H-21′ (δ_H_ 0.86) to C-20 and C-19′, from H-16′ (δ_H_ 2.38) to C-3′, C-4′, and C-5′, from H-3′ (δ_H_ 6.89) to C-1′, C-5′, C-7′, and C-16′, from H-5′ (δ_H_ 6.81) to C-3′, C-6′, C-7′, and C-16′, from H-1′ (δ_H_ 5.96) to C-2′, C-3′, C-7′, C-9′, C-13′, C-14′, C-15′, C-18′, and C-8′ (weak correlation), and from H-13′ (δ_H_ 6.05) to C-1′, C-9′, C-11′, C-14′, and C-10′ (weak correlation), above analysis, indicating an anthraquinone moiety (ABC-ring) with 1′-butyrate group. Moreover, the HMBC correlations from H-3 (δ_H_ 4.25) to C-2, C-4, C-5, and C-15, from H-5 (δ_H_ 2.65) to C-3, C-4, C-6, and C-7, from H-13 (δ_H_ 6.07) to C-9, C-11, and C-15′, and from 15-OCH_3_ (δ_H_ 3.67) to C-15 revealed the rest part as the xanthone moiety (DEF-ring). In addition, the key HMBC correlations from H-15′ (δ_H_ 2.74 and 2.68) to C-1′, C-9′, C-13′, C-14′, C-11, C-12, and C-13, and from OH-10 (δ_H_ 11.76) to C-9, C-14, C-11, and C-11′ (weak correlation) indicated that a methylene (C-15′) linked these two moieties at C-12 and C-14′, as well as C-11 connected to C-11′.

**Figure 2:**
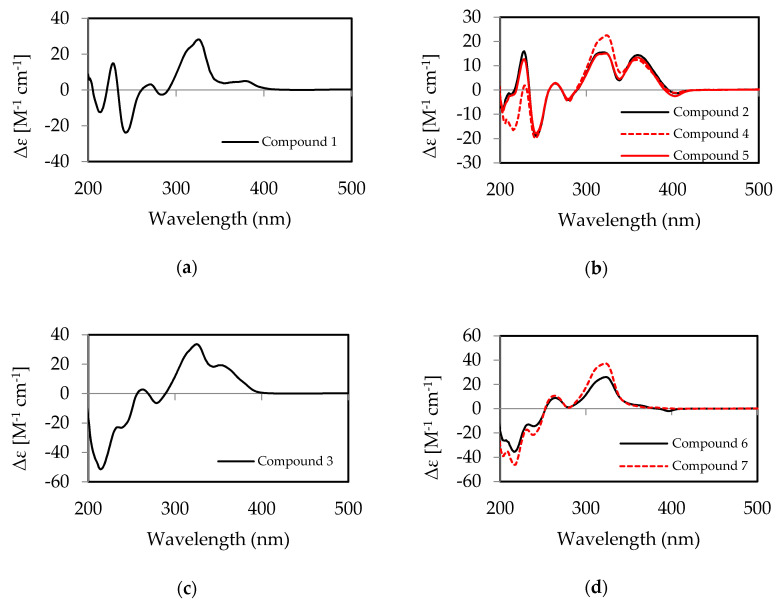
Electronic Circular Dichroism (ECD) spectra of compounds **1**–**7** measured in MeOH, (**a**) ECD spectrum of **1** (2*S*, 3*S*, 1′*S*, 11′*S*, and 14′*R*), (**b**) ECD spectra of **2** (2*S*, 3*S*, 1′*S*, 11′*S*, and 14′*R*), **4** (2*S*, 3*S*, 5*S*, 1′*S*, 11′*S*, and 14′*R*), and **5** (2*S*, 3*S*, 1′*S*, 11′*S*, and 14′*R*), (**c**) ECD spectrum of **3** (2*R*, 3*S*, 1′*S*, 11′*S*, and 14′*R*), (**d**) ECD spectra of **6** (2*R*, 3*S*, 1′*S*, 11′*S*, and 14′*R*) and **7** (2*R*, 3*S*, 1′*S*, 11′*S*, and 14′*R*).

Based on the combined above NMR analysis data and the molecular formula, the planar structure of **1** was elucidated as xanthone–anthraquinone heterodimer similar to xanthoquinodin A6 [21] and xanthoquinodin A9 [23]. The difference is at C-1′, where the hydroxyl is replaced by the butyl side chain of **1**. Noticeably, the *β*-keto-enol tautomeric system showed at C-8′ (δ_C_ 185.8) and C-10′ (δ_C_ 186.0), which both displayed keto carbonyl property in carbon chemical shift data. The Δ^12′,13′^ double-bond was revealed as *Z* for the small coupling constant (*J*_H12′H13′_ = 8.5 Hz). There are five chiral centers (C-2, C-3, C-1′, C-11′, and C-14′) in compound **1**, whose relative configuration was assigned by analysis of NOESY correlations and ^1^H,^1^H coupling constants. Since bridging carbons C-12′ and C-13′ must be on the same side, the relative configurations at C-11′ and C-14′ were deduced as *S* and *R*, respectively. In the NOESY spectrum, the strong intensity correlations of H-1′ with H*_α_*-15′ and H*_β_*-15′ indicated the S* configuration at C-1′. The methyl ester group was in axial bond orientation positioned between the F and G ring. A diaxial orientation was deduced for both H-3 and H-4a due to the large coupling constant (*J*_H3H4a_ = 12.3 Hz) between these protons. For the assignment of absolute configuration of **1**, the ECD spectrum (Figure 2a) was measured, and showed a similar pattern as the spectrum of xanthoquinodin A6 [22], proving that both compounds possessed the same stereochemistry. On the basis of the above data, the absolute configuration of compound **1** was assigned as 2*S*, 3*S*, 1′*S*, 11′*S*, and 14′*R*, and named xanthoquinodin A11.

Compound **2** was obtained as a yellow amorphous solid. The molecular ion cluster at *m*/*z* 645.1967 [M + H]^+^ in the HRESIMS spectrum indicated that the molecular formula of **2** was C_35_H_32_O_12_ (20 degrees of unsaturation). The ^1^H- and ^13^C-NMR spectra, accompanied with HSQC correlations, revealed signals of two methyl (δ_C_ 22.1, 13.5), one methoxy (δ_C_ 53.3), five sp^3^ methylenes (δ_C_ 36.2, 35.0, 27.7, 23.9, and 18.4), three sp^3^ methines (δ_C_ 72.7, 71.7, and 38.6), five aromatic methines (δ_C_ 132.3, 131.8, 123.2, 119.3, and 114.2), seventeen sp^2^ quaternary carbons, and two sp^3^ quaternary carbons (δ_C_ 41.6 and 85.3 (oxygenated)). The same molecular formulae and the resemblance of NMR spectroscopic data of **1** and **2** (Table 1) suggested that they were isomers. The main differences between ^13^C NMR spectrum of **1** and **2** were the upfield shifts at C-13 (Δδ –2.0) and C-14 (Δδ –3.6) in **2**, as well as the clearly downfield shifts at C-10 (Δδ +3.3) and C-11 (Δδ +3.3). Moreover, the strong HMBC correlations from OH-10 (δ_H_ 11.05) to aromatic methine δ_C_ 114.2 indicated that apart from the C-12–C-15′–C-14′ bridge, the two moiety (ABC-ring and FEG-ring) linked by C-13 connected to C-11′, which is similar to xanthoquinodin B series of structures. Meanwhile, the configurations of C-11′, C-14′, C-2, C-3, and 1′-butyrate were assigned as the same as those of **1**. Furthermore, the experimental ECD curves (Figure 2b) of **2** displayed the same as those previously given for xanthoquinodin B4 [23].

Therefore, the absolute configuration of **2** was assigned as 2*S*, 3*S*, 1′*S*, 11′*S*, and 14′*R*. The trivial name of xanthoquinodin B10 was given for compound **2**.

Compound **3** was obtained as a yellow amorphous solid with a molecular formula of C_35_H_32_O_12_ (20 degrees of unsaturation) based on the mass ion peak at *m*/*z* 645.1966 [M + H]^+^ in its HRESIMS spectrum. The ^1^H- and ^13^C-NMR spectra, accompanied with heteronuclear single quantum coherence (HSQC) correlations, revealed signals of two methyl (δ_C_ 22.1, 13.5), one methoxy (δ_C_ 53.6), five sp^3^ methylenes (δ_C_ 36.2, 35.0, 24.4, 22.9, and 18.4), three sp^3^ methines (δ_C_ 72.7, 66.9, and 37.9), five aromatic methines (δ_C_ 132.7, 131.6, 123.2, 119.3, and 114.7), seventeen sp^2^ quaternary carbons, and two sp^3^ quaternary carbons (δ_C_ 41.4 and 84.5 (oxygenated)).

**Figure 3:**
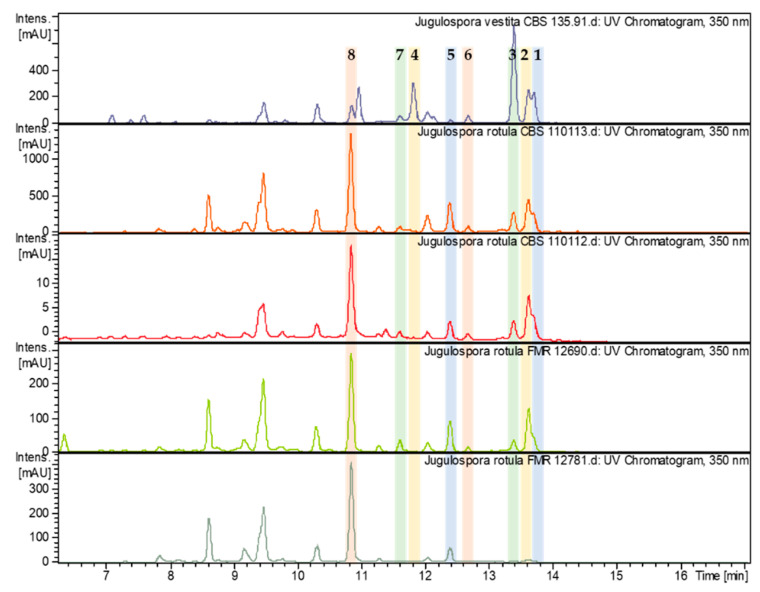
High performance liquid chromatography (HPLC) chromatograms (350 nm) of the ethyl acetate (EtOAc) extracts from the screened strains belonging to the genus *Jugulospora* with peaks of xanthoquinodins indicated by bold numbers referring to the molecules depicted in Figure 1.

The NMR spectroscopic data of **3** (Table 1) resembled those of **2**, with the main chemical shift difference (δ_C_ and δ_H_) located at the positions 2–7 in the F-ring, which indicated epimerization at C-2 or C-3. The small coupling constant (4.0 and 2.0 Hz) of the C-3 methine proton demonstrated an equatorial bond of H-3. Therefore, configurations of chiral carbons C-2 and C-3 were opposite, indicating that compound **2** and **3** were epimers at C-2. In addition, the experimental ECD spectra of compound **2** (Figure 2b) and **3** (Figure 2c) were different at 200–250 nm, which displayed similar patterns to those differentiated for xanthoquinodin B4 and xanthoquinodin B5 [22]. Therefore, the absolute configuration of **3** was assigned as 2*R*, 3*S*, 1′*S*, 11′*S*, and 14′*R*. Xanthoquinodin B11 was the name chosen for compound **3**.

Compound **4** was obtained as a yellow crystalline solid. The mass ion peak at *m*/*z* 661.1915 [M + H]^+^ in its HRESIMS spectrum indicated that the molecular formula of **4** was C_35_H_33_O_13_ (20 degrees of unsaturation). The ^1^H- and ^13^C-NMR spectra, accompanied with HSQC correlations, revealed signals of two methyl (δ_C_ 22.1, 13.5), onrfde methoxy (δ_C_ 53.4), four sp^3^ methylenes (δ_C_ 36.2, 35.1, 32.7, and 18.4), four sp^3^ methines (δ_C_ 72.7, 68.6, 65.9, and 38.7), five aromatic methines (δ_C_ 132.4, 131.7, 123.3, 119.4, and 114.3), seventeen sp^2^ quaternary carbons, and two sp^3^ quaternary carbons (δ_C_ 41.6 and 85.5 (oxygenated)). The NMR spectroscopic data of **4** (Table 1) resembled those of **2**, except for the main chemical shift difference (δ_C_ and δ_H_) located at the positions 3–6 in the F-ring, and C-5 (δ_C_ 65.9), which was oxygenated methine. Compared to **2**, there were one more oxygen and hydrogen atom in **4** according to the molecular formulae. On the basis of the above analysis, **4** was proposed as a new xanthoquinodin compound that possesses one additional hydroxyl substituent at the C-5. The configurations at C-2, C-3, C-1′, C-11′, and C-14′ were assigned as the same as those of **1** and **2** because of the similar corresponding NMR data (Table 1). The configuration at C-5 was confirmed as *S* based on the strong intensity ^1^H–^1^H COSY correlations of H-5 (δ_H_ 4.55, d (4.7)) with H_a_-4 (δ_H_ 2.42, m). However, it barely showed correlations with H_b_-4 (δ_H_ 2.23, m), indicating the dihedral right-angle of H-5–C-5–C-4–H_b_-4 and the bond of H-5 at the axial orientation consistent with the axial bond of H_a_-4 (δ_H_ 2.42, m), which showed large coupling constant (12.3 Hz) with H-3. Furthermore, compound **4** shared the similar experimental ECD curve with compound **2** (Figure 2b). Based on the above data, the absolute configuration of **4** was identified as 2*S*, 3*S*, 5*S*, 1′*S*, 11′*S*, and 14′*R*, and named xanthoquinodin B12.

Compound **5** was obtained as a yellow amorphous solid with a molecular formula of C_33_H_28_O_12_ (20 degrees of unsaturation) according to the mass ion peak at *m*/*z* 617.1650 [M + H]^+^ in its HRESIMS spectrum. The ^1^H- and ^13^C-NMR spectra, accompanied with HSQC correlations, revealed signals of two methyl (δ_C_ 35.0, 21.1), one methoxy (δ_C_ 53.3), three sp^3^ methylenes (δ_C_ 35.0, 27.7, and 23.9), three sp^3^ methines (δ_C_ 73.0, 71.8, and 38.6), five aromatic methines (δ_C_ 132.2, 131.8, 123.3, 119.4, and 114.3), seventeen sp^2^ quaternary carbons, and two sp^3^ quaternary carbons (δ_C_ 41.5 and 85.3 (oxygenated)). The NMR spectroscopic data of **5** (Table 2) were similar to those of **2** (Table 1), except for the absence of the butyrate group, replaced by acetate group at C-1′. In addition, the ECD spectrum of compound **5** showed the same pattern as those of compounds **2** and **4** (Figure 2b), corroborating that these compounds possessed the same stereochemistry. Besides, based on the same methods of analysis, the absolute configuration of **5** was proposed to be the same as that of **2**, being assigned as 2*S*, 3*S*, 1′*S*, 11′*S*, and 14′*R*. Compound **5** was named xanthoquinodin B13.

Compound **6** was obtained as a yellow amorphous solid. The mass ion peak at *m*/*z* 645.1967 [M + H]^+^ in its HRESIMS spectrum indicated that the molecular formula of **6** was C_35_H_32_O_12_ (20 degrees of unsaturation). The ^1^H- and ^13^C-NMR spectra, accompanied with HSQC correlations, revealed signals of two methyl (δ_C_ 22.1, 13.5), one methoxy (δ_C_ 53.8), six sp^3^ methylenes (δ_C_ 38.6, 36.2, 35.1, 27.7, 22.2, and 18.4), three sp^3^ methines (δ_C_ 80.8, 72.6, and 37.9), five aromatic methines (δ_C_ 132.9, 131.4, 123.2, 119.3, and 114.9), seventeen sp^2^ quaternary carbons, and two sp^3^ quaternary carbons (δ_C_ 41.5 and 84.9 (oxygenated)). The NMR spectroscopic data of **6** (Table 2) were similar to those of xanthoquinodin B6 [23], except for the substituent of C-1′, where the hydroxyl was replaced by the butyrate-like compound **1**. Furthermore, the NOESY correlations from H-1′ to H-15′ confirmed that 1′-hydroxyl was at the same orientation with the double bond of C-12′ and C-13′, consistent with all found xanthoquinodins. Likewise, the experimental ECD spectrum of compound **6** (Figure 2d) displayed a similar pattern at 200–250 nm to that of compound **3**, suggesting the same absolute configurations at C-2 and C-3, i.e., *R* and *S*, respectively. Thus, the absolute configuration of compound **6** was assigned as 2*R*, 3*S*, 1′*S*, 11′*S*, and 14′*R,* and the name given to it was xanthoquinodin B14.

Compound **7** was obtained as a yellow amorphous solid with a molecular formula of C_35_H_34_O_13_ (19 degrees of unsaturation) based on the mass ion peak at *m*/*z* 663.2072 [M + H]^+^ in its HRESIMS spectrum. The ^1^H- and ^13^C-NMR spectra, accompanied with HSQC correlations, revealed signals of two methyl (δ_C_ 22.1, 13.5), one methoxy (δ_C_ 53.4), six sp^3^ methylenes (δ_C_ 38.2, 36.2, 35.1, 30.0, 25.6, and 18.4), three sp^3^ methines (δ_C_ 73.8, 72.6, and 38.0), five aromatic methines (δ_C_ 132.9, 131.4, 123.2, 119.3, and 114.1), seventeen sp^2^ quaternary carbons, and two sp^3^ quaternary carbons (δ_C_ 41.4 and 87.3 (oxygenated)). The degrees of unsaturation and the observably different chemical shifts of C-2 to C-6 compared to compound **6** suggested the opening of the *γ*-lactone ring. The analysis of their NMR spectroscopic data (Table 2) and same experimental ECD curves (Figure 2d) revealed that **6** and **7** share the same absolute configuration as 2*R*, 3*S*, 1′*S*, 11′*S*, and 14′*R*, and **7** was named xanthoquinodin B15.

### 3.2. Antimicrobial and Cytotoxic Activities of Compounds ***1***–***8***

The eight compounds isolated showed antimicrobial activity against different fungi and/or different bacteria (Table 3).

Only compound **3** exhibited antifungal activity against *C. albicans, Mu. hiemalis*, *P. anomala*, and *R. glutinis,* with MIC values in a range of 2.10–16.70 µg/mL. However, the other compounds **1**, **2**, and **4**–**8** were not active or showed only weak activity (MIC 66.70 µg/mL) against the four tested fungi. A previous study has reported significant antifungal effects of xanthoquinodins A6 and ketoxanthoquinodin A6 against *Co. truncatum* and *Cu. lunata*, and of xathoquinodins B4 and B5 against *Al. brassicicola*, *Co. gloeosporioides*, *Co. truncatum*, *Cu. lunata* and *Py. grisea* [23].

All the compounds **1**–**8** were active against the three tested Gram-positive bacteria, i.e., *Mi. luteus*, *B. subtilis,* and *S. aureus*, with MIC values in a range of 0.20–8.30 µg/mL, but inactive against *M. smegmatis*. As for the tested Gram-negative bacteria, only compound **8** showed weak activity (MIC 66.70 µg/mL) against *Ch. violaceum*. This agrees with previous studies that demonstrated antibacterial activity of different xanthoquinodins against different Gram-positive bacteria, while activity against any Gram-negative bacteria tested was not observed [23,24].

The cytotoxicity results demonstrated that compounds **1**–**8** were active against all seven mammalian cell lines (Table 4). Interestingly, all those xanthoquinodins exhibited significant selective cytotoxicity against A431 human squamous cancer cells and MCF-7 human breast cancer cells with half maximal inhibitory concentrations (IC_50_) values in a range of 0.03–3.11 µM. Similarly, Anaya-Eugenio et al. reported that xanthoquinodin JBIR-99 exhibited high selective antiproliferative activity against PC-3 prostate cancer cells [25]. Compounds **1**–**3**, and **5**, which possess a F-ring (ρ-hydroxy hexatomic ring) and an ester group at C-1′, showed stronger cytotoxic activities against all tested cell lines, with IC_50_ values in a range of 0.03–1.46 µM, while compound **8** (possessing an OH group at C-1′ instead) displayed cytotoxic activities with IC_50_ values in a range of 0.10–4.70 µM. On the other hand, compounds **4**, **6**, and **7**, which possess a hydroxylated F-ring, γ-lactone ring, and a ring-opening respectively, showed cytotoxic activities against the tested cell lines, with IC_50_ values in a range of 1.03–18.6 µM. Cytotoxicity activities have been observed in different xanthoquinodins [22,23,24,26]. Sadorn et al. reported that xanthoquinodins A6, B4, and B5, which possessed an F-ring, showed stronger cytotoxicity against cell lines (NCI-H187 and Vero) than other xanthoquinodins with an open F-ring or a γ-lactone ring [23]. Furthermore, Chen et al. observed that xanthoquinodin A6 displayed significant cytotoxicity against all tested human cancer cell lines (HL-60, SMMC-7721, A-549, MCF-7, and SW480), with IC_50_ values in the range of 2.04–6.44 µM [22]. Therefore, the *p*-hydroxy hexatomic F-ring plays a key role in the structure–activity relationship of xanthoquinodins.

Moreover, some xanthoquinodins were reported to possess anticoccidial activity [27,28,29], as well as antimalarial activity [23]. Further studies need to be performed to confirm the bioactivity against coccidian protozoa and viruses of the new xanthoquinodins described here.

### 3.3. Comparison of Secondary Metabolite Production of Jugulospora spp.

During the course of the ongoing screening for novel biologically active secondary metabolites from the Sordariales, we observed that the ex-type strains of *Apiosordaria globosa* (CBS 110113) and *A. hispanica* (CBS 110112), now synonymyzed with *J. rotula* [14], produced similar chromatograms to the ex-type strain of *J. vestita*, with the six novel xanthoquinodins (**1**–**3**, **5**–**7**) and xanthoquinodin B4 also being present in both taxa. Moreover, we found the same novel compounds (except **4** and **7**) in two strains of *J. rotula* (FMR 12690 and FMR 12781), both isolated from soil samples, which is also the same substrate from which the ex-type strain of *J. vestita* and those of *A. globosa* and *A. hispanica* have been isolated. Even though all taxa belonging to the genus *Jugulospora* included in our study produced similar chromatograms, the production of the compounds was variable (see Figure 3). *Jugulospora rotula* CBS 110113 produced the xanthoquinodins in much larger amounts than the other strains, except for the compound **3**, which was produced by *J. vestita* CBS 135.91 as a major metabolite. On the other hand, *J. rotula* CBS 110112 produced much lower amounts of these compounds compared to the other four strains. *J. rotula* strains FMR 12690 and FMR 12781 produced higher quantity of compounds **5** and **8** and less of compounds **1**–**3** than the ex-type strain of *J. vestita*. Compound **7** was not detected in *J. rotula* FMR 12781, and compound **4** was not observed in any extracted strain of *J. rotula*. These data still rely on a limited number of experiments, and particularly the dependency of production on the culture medium and the time course of production remain to be studied further to get a better idea about their significance. However, they point towards the potential chemotaxonomic utility of xanthoquinodins in *Jugulospora* and allies.

**Figure 4:**
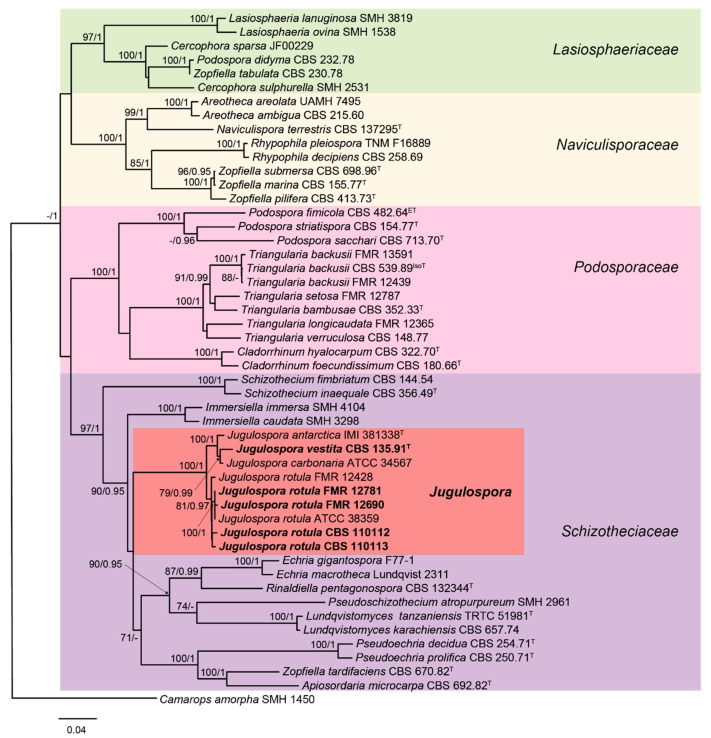
Randomized Axelerated Maximum Likelihood (RAxML) phylogram obtained from the combined sequences of the internal transcribed spacer region (ITS), the nuclear rDNA large subunit (LSU), and fragments of ribosomal polymerase II subunit 2 (*rpb2*) and β-tubulin (*tub2*) genes of selected strains belonging to the families Lasiosphaeriaceae, Naviculisporaceae, Podosporaceae, and Schizotheciaceae. *Camarops amorpha* SMH 1450 was used as an outgroup. Bootstrap support values ≥ 70/Bayesian posterior probability scores ≥0.95 are indicated along branches. Branch lengths are proportional to distance. Screened taxa in the present study are in **bold**. Ex-epitype, ex-isotype, and ex-type strains of the different species are indicated with ^ET^, ^IsoT^, and ^T^, respectively. GenBank accession numbers were indicated in Marin-Felix et al. [14], as well as the methodology followed for performing the phylogenetic study. The major clades are marked in different colors for the sake of better readability.

The genus *Apiosordaria* is polyphyletic and scattered along the also polyphyletic family Lasiosphaeriaceae (order Sordariales) [30]. The main problem in the delimitation of lasiosphaeriaceous taxa is that the traditional circumscription based on the ascospore morphology is artificial, being that this an extremely homoplastic character not useful in predicting phylogenetic relationships [31,32]. Even though the structure of the ascomatal wall is clearly more useful for delimitation of some genera, it is not always useful [32]. Recently, *Apiosordaria* was synonymized with *Triangularia* and placed in the family Podosporaceae based on phylogenetic data [30]. However, several species of the genus, such as *A. microcarpa*, remain improperly taxonomically placed (see Figure 4). In that context, the new combination *Jugulospora vestita* has been recently proposed to accommodate *A. vestita*, in the family Schizotheciaceae, according to a phylogenetic study based on the ITS, LSU, *rpb2,* and *tub2* sequences [14].

In the same context, *A. globosa* and *A. hispanica* were synonymized with *J. rotula*. As we mentioned before, all these taxa now included in the genus *Jugulospora* produced similar chromatograms and compounds. However, the other species of *Apiosordaria* included in the screening study, i.e., *A. backusii* (now transferred to *Triangularia* [30]), produced completely different unrelated compounds (data not shown). Therefore, the production of secondary metabolites could be suitable as chemotaxonomic markers, as demonstrated before in other groups of fungi such as the Xylariales [8,33,34], helping to achieve a more natural classification of lasiosphaeriaceous taxa.

Finally, it is important to mention that some of the xanthoquinodins reported to date were found in members also of the Sordariales (which includes the genus *Jugulospora*), i.e., xanthoquinodins Al, A2, A3, Bl, B2, and B3 in *Humicola* sp. [28,29] and xanthoquinodins A4, A5, A6, B4, and B5 in *Chaetomium elatum* [22].

## 4. Conclusions

The present study led to the isolation of eight cytotoxic and antimicrobial compounds, seven of which turned out to be new to science. Therefore, the potential of Sordariomycetes as prolific producers of bioactive secondary metabolites was again demonstrated here. Moreover, the production of these compounds by *Jugulospora vestita* and other strains belonging to the same genus, but not by other related genera of Sordariales, suggests that the production of secondary metabolites could be suitable as chemotaxonomic markers.

## Figures and Tables

**Figure 1:**
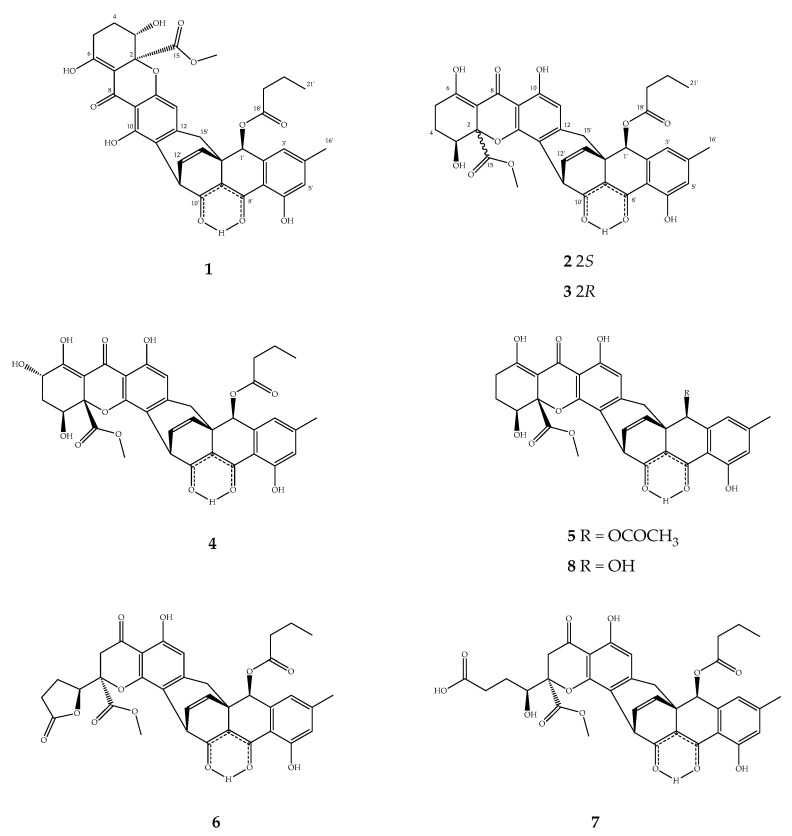
Chemical Structures of Compounds **1**–**8**.

**Table 1:** One-dimensional nuclear magnetic resonance (1D-NMR) spectroscopic data for compounds **1**–**4** (in CDCl_3_).

	1		2		3		4	
No	*δ*_C_, Type	*δ*_H_ (*J* in Hz)	*δ*_C_, Type	*δ*_H_ (*J* in Hz)	*δ*_C_, Type	*δ*_H_ (*J* in Hz)	*δ*_C_, Type	*δ*_H_ (*J* in Hz)
2	84.3, C	–	85.3, C	–	84.5, C	–	85.5, C	–
3	71.8, CH	4.25, dd (12.3, 5.3)	71.7, CH	4.46, dd (12.3, 5.0)	66.9, CH	4.49, dd (4.0, 2.0)	68.6, CH	4.74, dd (12.7, 5.1)
4	23.7, CH_2_	2.15, m, H_a_ 2.08, m, H_b_	23.9, CH_2_	2.21, m, H_a_ 2.10, m, H_b_	22.9, CH_2_	2.18, m, H_a_ 2.00, m, H_b_	32.7, CH_2_	2.42, m, H_a_ 2.23, m, H_b_
5	27.5, CH_2_	2.65, m	27.7, CH_2_	2.69, m	24.4, CH_2_	2.84, m 2.44, m	65.9, CH	4.55, d (4.7)
6	178.1, C	–	178.5, C	–	180.2, C	–	174.0, C	–
7	101.1, C	–	101.6, C	–	100.2, C	–	101.8, C	–
8	186.5, C	–	186.7, C	–	186.9, C	–	188.0, C	–
9	104.9, C	–	105.4, C	–	105.3, C	–	105.8, C	–
10	156.8, C	–	160.1, C	–	160.2, C	–	160.1, C	–
11	117.3, C	–	114.2, CH	6.13, s	114.7, CH	6.15, s	114.3, CH	6.14, s
12	147.8, C	–	147.6, C	–	147.3, C	–	148.5, C	–
13	110.9, CH	6.07, s	115.3, C	–	115.2, C	–	115.5, C	–
14	158.4, C	–	154.8, C	–	153.6, C	–	155.0, C	–
15	169.6, C	–	169.8, C	–	171.0, C	–	169.2, C	–
1′	72.8, CH	5.96, s	72.7, CH	5.98, s	72.7, CH	5.98, s	72.7, CH	5.98, s
2′	136.6, C	–	136.5, C	–	136.6, C	–	136.5, C	–
3′	123.1, CH	6.89, s	123.2, CH	6.90, s	123.2, CH	6.90, s	123.3, CH	6.90, s
4′	147.6, C	–	147.7, C	–	147.8, C	–	147.8, C	–
5′	119.3, CH	6.81, s	119.3, CH	6.80, s	119.3, CH	6.80, s	119.4, CH	6.80, s
6′	161.7, C	–	161.6, C	–	161.7, C	–	161.5, C	–
7′	112.7, C	–	112.4, C	–	112.6, C	–	112.3, C	–
8′	185.8, C	–	185.3, C	–	185.2, C	–	184.8, C	–
9′	105.5, C	–	105.6, C	–	105.7, C	–	105.6, C	–
10′	186.0, C	–	186.1, C	–	186.4, C	–	186.4, C	–
11′	37.3, CH	4.76, dd (6.6, 1.0)	38.6, CH	4.81, dd (6.7, 0.7)	37.9, CH	4.76, dd (6.7, 1.0)	38.7, CH	4.83, dd (6.6, 0.9)
12′	131.9, CH	6.42, dd (8.5, 6.6)	131.8, CH	6.48, dd (8.4, 6.7)	131.6, CH	6.41, dd (8.4, 6.7)	131.7, CH	6.47, dd (8.4, 6.6)
13′	132.4, CH	6.05, dd (8.5, 1.0)	132.3, CH	6.08, dd (8.4, 0.7)	132.7, CH	6.07, dd (8.4, 1.0)	132.4, CH	6.08, d (8.4, 0.9)
14′	41.5, C	–	41.6, C		41.4, C	–	41.6, C	–
15′	35.1, CH_2_	2.74, d (18.5), H_a_ 2.68, d (18.5), H_b_	35.0, CH_2_	2.79, d (18.0), H_a_ 2.67, d (18.0), H_b_	35.0, CH_2_	2.78, d (17.9), H_a_ 2.68, d (17.9), H_b_	35.1, CH_2_	2.80, d (18.0), H_a_ 2.69, d (18.0), H_b_
16′	22.0, CH_3_	2.38, s	22.1, CH_3_	2.38, s	22.1, CH_3_	2.38, s	22.1, CH_3_	2.38, s
18′	173.1, C	–	173.1, C	–	173.0, C	–	173.1, C	–
19′	36.2, CH_2_	2.22, m	36.2, CH_2_	2.23, m	36.2, CH_2_	2.22, m	36.2, CH_2_	2.22, m
20′	18.4, CH_2_	1.57, m	18.4, CH_2_	1.58, m	18.4, CH_2_	1.57, m	18.4, CH_2_	1.57, m
21′	13.5, CH_3_	0.86, t (7.4)	13.5, CH_3_	0.86, t (7.4)	13.5, CH_3_	0.86, t (7.4)	13.5, CH_3_	0.86, t (7.4)
15- OCH_3_	53.4, CH_3_	3.67, s	53.3, CH_3_	3.73, s	53.6, CH_3_	3.74, s	53.4, CH_3_	3.73, s
3-OH	–	14.14	–	14.26	–	–	–	–
6-OH	–	13.84	–	13.95	–	14.10	–	13.70
10-OH	–	11.76	–	11.05	–	11.24	–	10.91
6′-OH	–	11.58	–	11.40	–	11.55	–	11.30

**Table 2:** 1D-NMR spectroscopic data for compounds **5**–**7** (in CDCl_3_).

	5		6		7	
No	*δ*_C_, Type	*δ*_H_ (*J* in Hz)	*δ*_C_, Type	*δ*_H_ (*J* in Hz)	*δ*_C_, Type	*δ*_H_ (*J* in Hz)
2	85.3, C	–	84.9, C	–	87.3, C	–
3	71.8, CH	4.46, dd (12.5, 5.0)	80.8, CH	5.01, dd (7.6, 6.8)	73.8, CH	4.23, dd (10.8, 1.8)
4	23.9, CH_2_	2.23, m 2.13, m	22.2, CH_2_	2.42, m	25.6, CH_2_	1.97, m 1.78, m
5	27.7, CH_2_	2.70, m	27.7, CH_2_	2.67, m	30.0, CH_2_	2.69, m
6	178.5, C	–	175.2, C	–	177.2, C	–
7	101.6, C	–	38.6, CH_2_	3.21, d (17.0) 3.04, d (17.0)	38.2, CH_2_	3.20, s
8	186.8, C	–	194.1, C	–	195.7, C	–
9	105.4, C	–	105.8, C	–	105.9, C	–
10	160.1, C	–	160.1, C	–	160.1, C	–
11	114.3, CH	6.14, s	114.9, CH	6.16, s	114.1, CH	6.14, s
12	147.6, C	–	148.9, C	–	147.8, C	–
13	115.3, C	–	114.9, C	–	114.8, C	–
14	154.7, C	–	154.8, C	–	155.1, C	–
15	169.8, C	–	168.7, C	–	170.0, C	–
1′	73.0, CH	5.97, s	72.6, CH	5.98, s	72.6, CH	5.98, s
2′	136.4, C	–	136.5, C	–	136.5, C	–
3′	123.3, CH	6.90, s	123.2, CH	6.89, s	123.2, CH	6.90, s
4′	147.8, C	–	147.8, C	–	147.8, C	–
5′	119.4, CH	6.81, s	119.3, CH	6.80, s	119.3, CH	6.80, s
6′	161.7, C	–	161.8, C	–	161.7, C	–
7′	112.5, C	–	112.7, C	–	112.6, C	–
8′	185.6, C	–	184.8, C	–	185.4, C	–
9′	105.6, C	–	105.4, C	–	105.5, C	–
10′	185.9, C	–	186.6, C	–	186.2, C	–
11′	38.6, CH	4.79, d (6.7)	37.9, CH	4.65, dd (6.6, 0.9)	38.0, CH	4.73, dd (6.6, 0.6)
12′	131.8, CH	6.49, dd (8.5, 6.7)	131.4, CH	6.41, dd (8.5, 6.6)	131.4, CH	6.42, dd (8.5, 6.6)
13′	132.2, CH	6.09, d (8.5)	132.9, CH	6.09, d (8.5, 0.9)	132.9, CH	6.09, dd (8.5, 0.6)
14′	41.5, C	–	41.5, C	–	41.4, C	–
15′	35.0, CH_2_	2.79, d (17.5) 2.68, d (17.5)	35.1, CH_2_	2.79, d (18.2) 2.68, d (18.2)	35.1, CH_2_	2.79, d (17.5) 2.67, d (17.5)
16′	22.1, CH_3_	2.38, s	22.1, CH_3_	2.38, s	22.1, CH_3_	2.38, s
18′	170.4, C	–	173.1, C	–	173.1, C	–
19′	21.1, CH_3_	2.02, s	36.2, CH_2_	2.22, m	36.2, CH_2_	2.21, m
20′	–	–	18.4, CH_2_	1.57, m	18.4, CH_2_	1.58, m
21′	–	–	13.5, CH_3_	0.86, t (7.4)	13. 5, CH_3_	0.86, t (7.4)
15- OCH_3_	53.3, CH_3_	3.74, s	53.8, CH_3_	3.75, s	53.4, CH_3_	3.73, s
3-OH	–	–	–	–	–	–
6-OH	–	13.95	–	–	–	–
10-OH	–	11.05	–	11.36	–	11.5
6′-OH	–	11.45	–	11.60	–	–

**Table 3:** Minimum inhibitory concentration (MIC, µg/mL) of **1**–**8** against bacterial and fungal test organisms.

Test Organism	1	2	3	4	5	6	7	8	Positive Control
*Schizosaccharomyces pombe*	–	–	66.70	–	–	–	–	–	33.30 ^1^
*Pichia anomala*	–	–	8.30	–	–	–	–	–	33.30 ^1^
*Mucor hiemalis*	66.70	–	2.10	66.70	66.70	66.70	66.70	–	33.30 ^1^
*Candida albicans*	–	–	16.70	–	–	–	–	–	33.30 ^1^
*Rhodotorula glutinis*	–	–	2.10	66.70	–	–	–	–	16.70 ^1^
*Micrococcus luteus*	4.20	2.10	2.10	2.10	2.10	8.30	4.20	8.30	0.80 ^2^
*Bacillus subtilis*	0.40	0.40	0.20	0.80	0.20	4.20	4.20	2.10	8.30 ^2^
*Staphylococcus aureus*	2.10	2.10	1.00	2.10	8.30	4.20	8.30	8.30	0.40 ^2^
*Mycobacterium smegmatis*	–	–	–	–	–	–	–	–	1.70 ^3^
*Escherichia coli*	–	–	–	–	–	–	–	–	3.30 ^2^
*Pseudomonas aeruginosa*	–	–	–	–	–	–	–	–	0.40 ^4^
*Chromobacterium violaceum*	–	–	–	–	–	–	–	66.70	0.80 ^2^

^1^ nystatin, ^2^ oxytetracycline, ^3^ kanamycin, ^4^ gentamicin, –: no inhibition observed under test conditions.

**Table 4:** Cytotoxicity of **1**–**8** against mammalian cell lines [half maximal inhibitory concentrations (IC_50_): µM].

Compound	KB 3.1	L929	A549	SK-OV-3	PC-3	A431	MCF-7
**1**	0.20	1.13	0.28	0.14	0.71	0.05	0.06
**2**	0.19	1.46	0.40	0.13	1.06	0.06	0.04
**3**	0.15	0.98	0.26	0.15	0.65	0.05	0.04
**4**	5.76	13.48	9.39	4.09	8.18	2.27	1.97
**5**	0.19	1.12	0.29	0.14	0.62	0.06	0.03
**6**	3.42	10.56	18.63	3.88	5.43	3.11	1.10
**7**	3.47	9.97	11.48	3.93	4.98	2.87	1.03
**8**	1.06	4.70	1.22	0.47	3.14	0.16	0.10
epothilon B	0.00003	0.00051	0.00009	0.00009	0.00007	0.00005	0.00003

## Supplementary Materials

The following are available online at https://www.mdpi.com/2309-608X/6/4/188/s1, Figure S1: ^1^H NMR spectrum (500 MHz, chloroform-*d*) of xanthoquinodin A11 (**1**), Figure S2: ^13^C NMR spectrum (126 MHz, chloroform-*d*) of xanthoquinodin A11 (**1**), Figure S3: HSQC NMR spectrum (500 MHz, chloroform-*d*) of xanthoquinodin A11 (**1**), Figure S4: COSY NMR spectrum (500 MHz, chloroform-*d*) of xanthoquinodin A11 (**1**), Figure S5: HMBC NMR spectrum (500 MHz, chloroform-*d*) of xanthoquinodin A11 (**1**), Figure S6: NOESY NMR spectrum (500 MHz, chloroform-*d*) of xanthoquinodin A11 (**1**), Figure S7: HRESIMS data of xanthoquinodin A11 (**1**), Figure S8: ^1^H NMR spectrum (500 MHz, chloroform-*d*) of xanthoquinodin B10 (**2**), Figure S9: ^13^C NMR spectrum (125 MHz, chloroform-*d*) of xanthoquinodin B10 (**2**), Figure S10: HSQC NMR spectrum (500 MHz, chloroform-*d*) of xanthoquinodin B10 (**2**), Figure S11: COSY NMR spectrum (500 MHz, chloroform-*d*) of xanthoquinodin B10 (**2**), Figure S12: HMBC NMR spectrum (500 MHz, chloroform-*d*) of xanthoquinodin B10 (**2**), Figure S13: NOESY NMR spectrum (500 MHz, chloroform-*d*) of xanthoquinodin B10 (**2**), Figure S14: HRESIMS data of xanthoquinodin B10 (**2**), Figure S15: ^1^H NMR spectrum (500 MHz, chloroform-*d*) of xanthoquinodin B11 (**3**), Figure S16: ^13^C NMR spectrum (125 MHz, chloroform-*d*) of xanthoquinodin B11 (**3**), Figure S17: HSQC NMR spectrum (500 MHz, chloroform-*d*) of xanthoquinodin B11 (**3**), Figure S18: COSY NMR spectrum (500 MHz, chloroform-*d*) of xanthoquinodin B11 (**3**), Figure S19: HMBC NMR spectrum (500 MHz, chloroform-*d*) of xanthoquinodin B11 (**3**), Figure S20: NOESY NMR spectrum (500 MHz, chloroform-*d*) of xanthoquinodin B11 (**3**), Figure S21: HRESIMS data of xanthoquinodin B11 (**3**), Figure S22: ^1^H NMR spectrum (700 MHz, chloroform-*d*) of xanthoquinodin B12 (**4**), Figure S23: ^13^C NMR spectrum (125 MHz, chloroform-*d*) of xanthoquinodin B12 (**4**), Figure S24: HSQC NMR spectrum (500 MHz, chloroform-*d*) of xanthoquinodin B12 (**4**), Figure S25: COSY NMR spectrum (500 MHz, chloroform-*d*) of xanthoquinodin B12 (**4**), Figure S26: HMBC NMR spectrum (700 MHz, chloroform-*d*) of xanthoquinodin B12 (**4**), Figure S27: NOESY NMR spectrum (500 MHz, chloroform-*d*) of xanthoquinodin B12 (**4**), Figure S28: HRESIMS data of xanthoquinodin B12 (**4**), Figure S29: ^1^H NMR spectrum (700 MHz, chloroform-*d*) of xanthoquinodin B13 (**5**), Figure S30: ^13^C NMR spectrum (125 MHz, chloroform-*d*) of xanthoquinodin B13 (**5**), Figure S31: HSQC NMR spectrum (700 MHz, chloroform-*d*) of xanthoquinodin B13 (**5**), Figure S32: COSY NMR spectrum (700 MHz, chloroform-*d*) of xanthoquinodin B13 (**5**), Figure S33: HMBC NMR spectrum (700 MHz, chloroform-*d*) of xanthoquinodin B13 (**5**), Figure S34: NOESY NMR spectrum (700 MHz, chloroform-*d*) of xanthoquinodin B13 (**5**), Figure S35: HRESIMS data of xanthoquinodin B13 (**5**), Figure S36: ^1^H NMR spectrum (700 MHz, chloroform-*d*) of xanthoquinodin B14 (**6**), Figure S37: ^13^C NMR spectrum (176 MHz, chloroform-*d*) of xanthoquinodin B14 (**6**), Figure S38: HSQC NMR spectrum (700 MHz, chloroform-*d*) of xanthoquinodin B14 (**6**), Figure S39: COSY NMR spectrum (700 MHz, chloroform-*d*) of xanthoquinodin B14 (**6**), Figure S40: HMBC NMR spectrum (700 MHz, chloroform-*d*) of xanthoquinodin B14 (**6**), Figure S41: NOESY NMR spectrum (700 MHz, chloroform-*d*) of xanthoquinodin B14 (**6**), Figure S42: HRESIMS data of xanthoquinodin B14 (**6**), Figure S43: ^1^H NMR spectrum (500 MHz, chloroform-*d*) of xanthoquinodin B15 (**7**), Figure S44: ^13^C NMR spectrum (125 MHz, chloroform-*d*) of xanthoquinodin B15 (**7**), Figure S45: HSQC NMR spectrum (500 MHz, chloroform-*d*) of xanthoquinodin B15 (**7**), Figure S46: COSY NMR spectrum (500 MHz, chloroform-*d*) of xanthoquinodin B15 (**7**), Figure S47: HMBC NMR spectrum (500 MHz, chloroform-*d*) of xanthoquinodin B15 (**7**), Figure S48: NOESY NMR spectrum (500 MHz, chloroform-*d*) of xanthoquinodin B15 (**7**), Figure S49: HRESIMS data of xanthoquinodin B15 (**7**).
