# Supplementary material for: Seven New Cytotoxic and Antimicrobial Xanthoquinodins from Jugulospora vestita
Source: J Fungi (Basel). 2020 Sep 25;6(4):188. doi: 10.3390/jof6040188 (PMC7712541; doi:10.3390/jof6040188)
Supplement: Supplementary file 1 [file jof-06-00188-s001.pdf]

Supporting Information for

Seven new Cytotoxic and Antimicrobial Xanthoquinodins from *Jugulospora vestita*

Lulu Shao [1,2,3], Yasmina Marin-Felix [1,*], Frank Surup [1], Alberto M. Stchigel [4] and Marc Stadler [1]

[1] Department Microbial Drugs, Helmholtz Centre for Infection Research, Inhoffenstrasse 7, 38124 Braunschweig, Germany; Lulu.Shao@helmholtz-hzi.de (L.S.); Yasmina.MarinFelix@helmholtz-hzi.de (Y.M.F.); Frank.Surup@helmholtz-hzi.de (F.S.); Marc.Stadler@helmholtz-hzi.de (M.S.)

[2] South China Botanical Garden, Chinese Academy of Sciences, Xingke Road 723, Tianhe District, Guangzhou 510650, People's Republic of China; Lulu.Shao@helmholtz-hzi.de (L.S.)

[3] School of Life Sciences, University of Chinese Academy of Sciences, Yuquanlu 19A, Beijing 100049, People's Republic of China; Lulu.Shao@helmholtz-hzi.de (L.S.)

[4] Mycology Unit, Medical School and IISPV, Universitat Rovira i Virgili, C/ Sant Llorenç 21, 43201 Reus, Tarragona, Spain; albertomiguel.stchigel@urv.cat (A.M.S.)

* Correspondence: Yasmina.MarinFelix@helmholtz-hzi.de

Contents

Figure S1. ^{1}H NMR spectrum (500 MHz, chloroform-*d*) of xanthoquinodin A11 (**1**)....4
Figure S2. ^{13}C NMR spectrum (126 MHz, chloroform-*d*) of xanthoquinodin A11 (**1**)....5
Figure S3. HSQC NMR spectrum (500 MHz, chloroform-*d*) of xanthoquinodin A11 (**1**)....6
Figure S4. COSY NMR spectrum (500 MHz, chloroform-*d*) of xanthoquinodin A11 (**1**)....7
Figure S5. HMBC NMR spectrum (500 MHz, chloroform-*d*) of xanthoquinodin A11 (**1**)....8
Figure S6. NOESY NMR spectrum (500 MHz, chloroform-*d*) of xanthoquinodin A11 (**1**)....9
Figure S7. HRESIMS data of xanthoquinodin A11 (**1**)....10
Figure S8. ^{1}H NMR spectrum (500 MHz, chloroform-*d*) of xanthoquinodin B10 (**2**)....11
Figure S9. ^{13}C NMR spectrum (125 MHz, chloroform-*d*) of xanthoquinodin B10 (**2**)....12
Figure S10. HSQC NMR spectrum (500 MHz, chloroform-*d*) of xanthoquinodin B10 (**2**)....13
Figure S11. COSY NMR spectrum (500 MHz, chloroform-*d*) of xanthoquinodin B10 (**2**)....14
Figure S12. HMBC NMR spectrum (500 MHz, chloroform-*d*) of xanthoquinodin B10 (**2**)....15
Figure S13. NOESY NMR spectrum (500 MHz, chloroform-*d*) of xanthoquinodin B10 (**2**)....16
Figure S14. HRESIMS data of xanthoquinodin B10 (**2**)....17
Figure S15. ^{1}H NMR spectrum (500 MHz, chloroform-*d*) of xanthoquinodin B11 (**3**)....18
Figure S16. ^{13}C NMR spectrum (125 MHz, chloroform-*d*) of xanthoquinodin B11 (**3**)....19
Figure S17. HSQC NMR spectrum (500 MHz, chloroform-*d*) of xanthoquinodin B11 (**3**)....20
Figure S18. COSY NMR spectrum (500 MHz, chloroform-*d*) of xanthoquinodin B11 (**3**)....21
Figure S19. HMBC NMR spectrum (500 MHz, chloroform-*d*) of xanthoquinodin B11 (**3**)....22
Figure S20. NOESY NMR spectrum (500 MHz, chloroform-*d*) of xanthoquinodin B11 (**3**)....23
Figure S21. HRESIMS data of xanthoquinodin B11 (**3**)....24
Figure S22. ^{1}H NMR spectrum (700 MHz, chloroform-*d*) of xanthoquinodin B12 (**4**)....25
Figure S23. ^{13}C NMR spectrum (125 MHz, chloroform-*d*) of xanthoquinodin B12 (**4**)....26
Figure S24. HSQC NMR spectrum (500 MHz, chloroform-*d*) of xanthoquinodin B12 (**4**)....27
Figure S25. COSY NMR spectrum (500 MHz, chloroform-*d*) of xanthoquinodin B12 (**4**)....28
Figure S26. HMBC NMR spectrum (700 MHz, chloroform-*d*) of xanthoquinodin B12 (**4**)....29
Figure S27. NOESY NMR spectrum (500 MHz, chloroform-*d*) of xanthoquinodin B12 (**4**)....30
Figure S28. HRESIMS data of xanthoquinodin B12 (**4**)....31
Figure S29. ^{1}H NMR spectrum (700 MHz, chloroform-*d*) of xanthoquinodin B13 (**5**)....32

Figure S30. ^{13}C NMR spectrum (125 MHz, chloroform-*d*) of xanthoquinodin B13 (**5**)....33
Figure S31. HSQC NMR spectrum (700 MHz, chloroform-*d*) of xanthoquinodin B13 (**5**) 34
Figure S32. COSY NMR spectrum (700 MHz, chloroform-*d*) of xanthoquinodin B13 (**5**)....35
Figure S33. HMBC NMR spectrum (700 MHz, chloroform-*d*) of xanthoquinodin B13 (**5**) 36
Figure S34. NOESY NMR spectrum (700 MHz, chloroform-*d*) of xanthoquinodin B13 (**5**) 37
Figure S35. HRESIMS data of xanthoquinodin B13 (**5**)....38
Figure S36. ^{1}H NMR spectrum (700 MHz, chloroform-*d*) of xanthoquinodin B14 (**6**)....39
Figure S37. ^{13}C NMR spectrum (176 MHz, chloroform-*d*) of xanthoquinodin B14 (**6**)....40
Figure S38. HSQC NMR spectrum (700 MHz, chloroform-*d*) of xanthoquinodin B14 (**6**) 41
Figure S39. COSY NMR spectrum (700 MHz, chloroform-*d*) of xanthoquinodin B14 (**6**)....42
Figure S40. HMBC NMR spectrum (700 MHz, chloroform-*d*) of xanthoquinodin B14 (**6**) 43
Figure S41. NOESY NMR spectrum (700 MHz, chloroform-*d*) of xanthoquinodin B14 (**6**) 44
Figure S42. HRESIMS data of xanthoquinodin B14 (**6**)....45
Figure S43. ^{1}H NMR spectrum (500 MHz, chloroform-*d*) of xanthoquinodin B15 (**7**)....46
Figure S44. ^{13}C NMR spectrum (125 MHz, chloroform-*d*) of xanthoquinodin B15 (**7**)....47
Figure S45. HSQC NMR spectrum (500 MHz, chloroform-*d*) of xanthoquinodin B15 (**7**) 48
Figure S46. COSY NMR spectrum (500 MHz, chloroform-*d*) of xanthoquinodin B15 (**7**)....49
Figure S47. HMBC NMR spectrum (500 MHz, chloroform-*d*) of xanthoquinodin B15 (**7**) 50
Figure S48. NOESY NMR spectrum (500 MHz, chloroform-*d*) of xanthoquinodin B15 (**7**) 51
Figure S49. HRESIMS data of xanthoquinodin B15 (**7**)....52

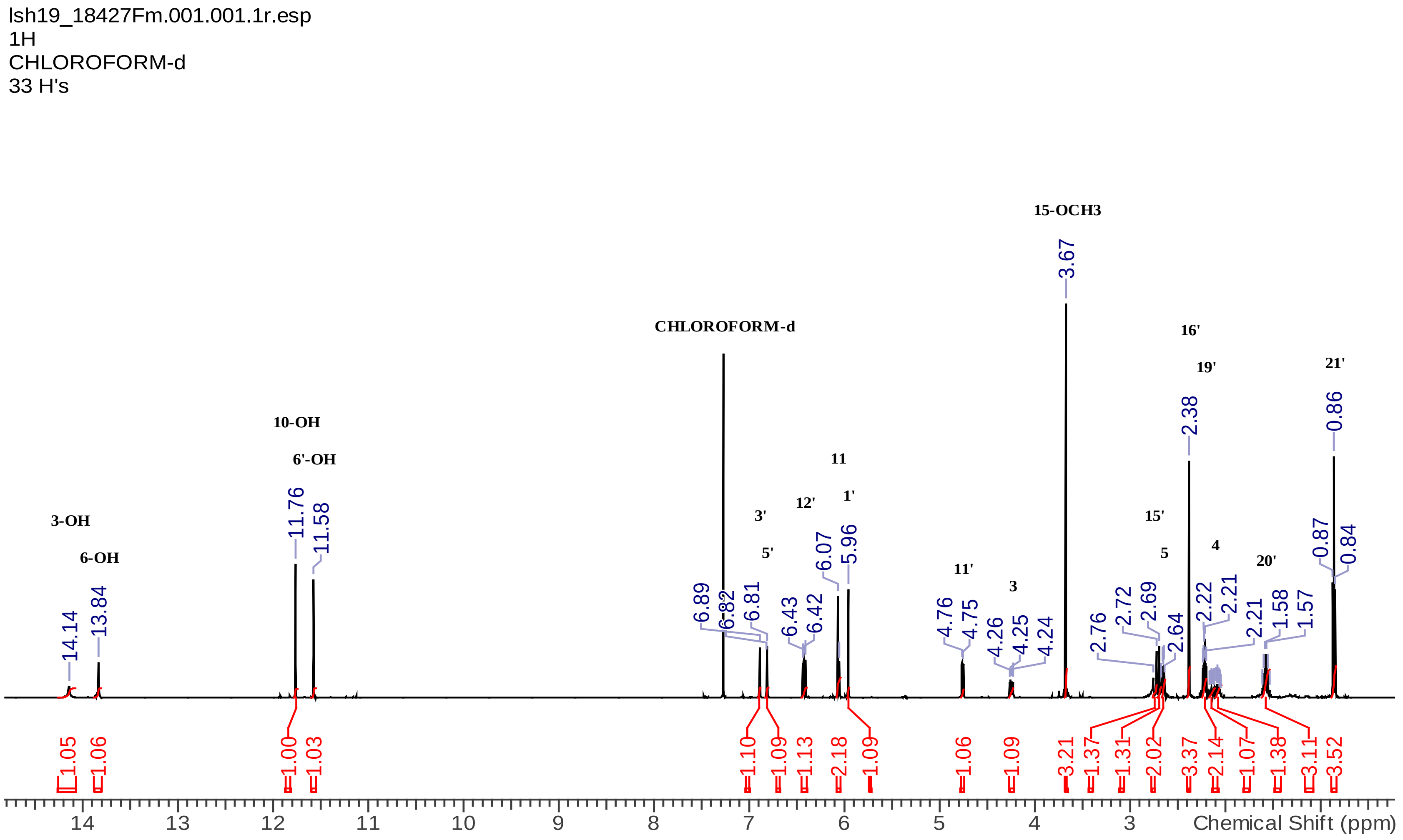

Figure S1. ^{1}H NMR spectrum (500 MHz, chloroform-*d*) of xanthoquinodin A11 (**1**)

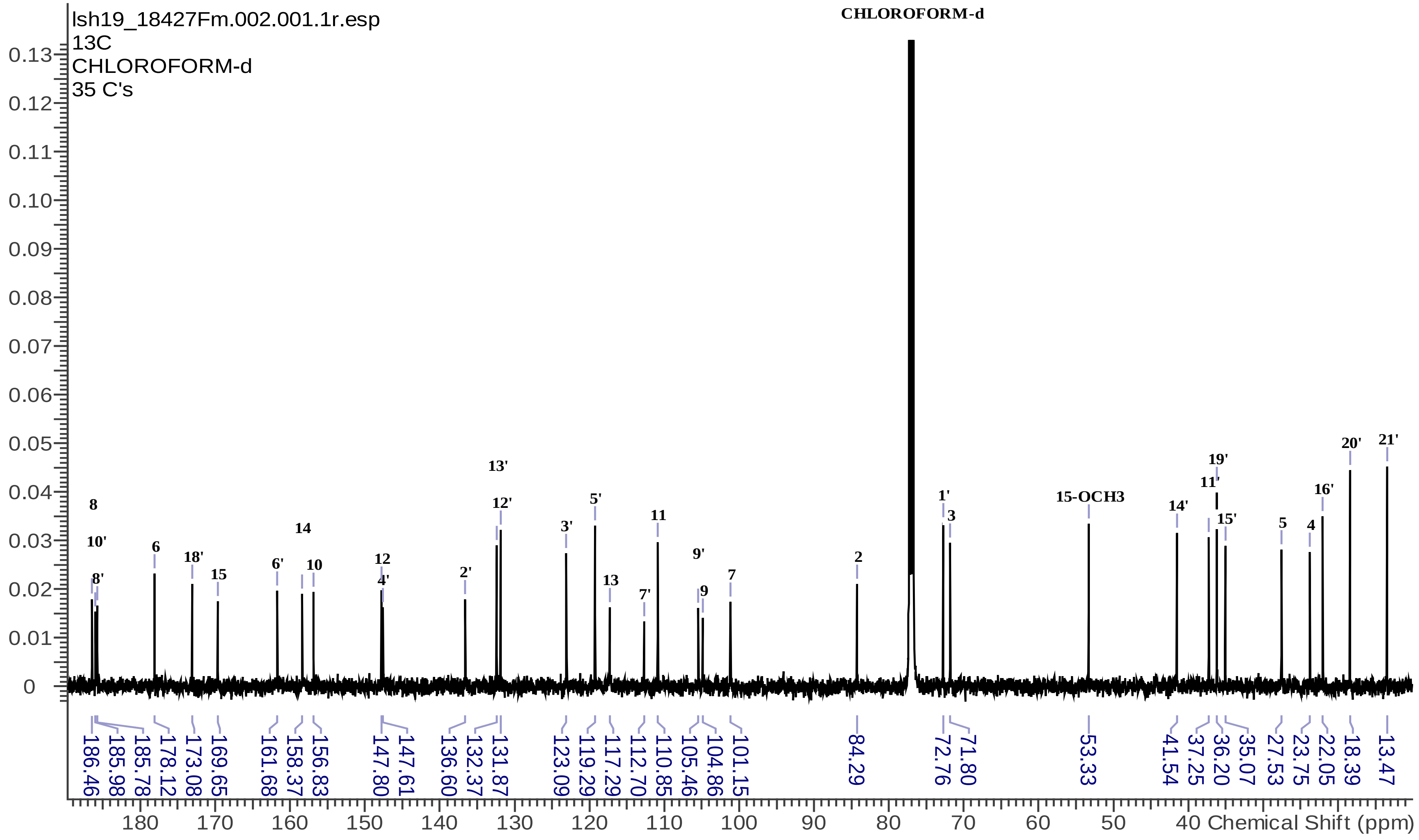

Figure S2. ^{13}C NMR spectrum (126 MHz, chloroform-*d*) of xanthoquinodin A11 (**1**)

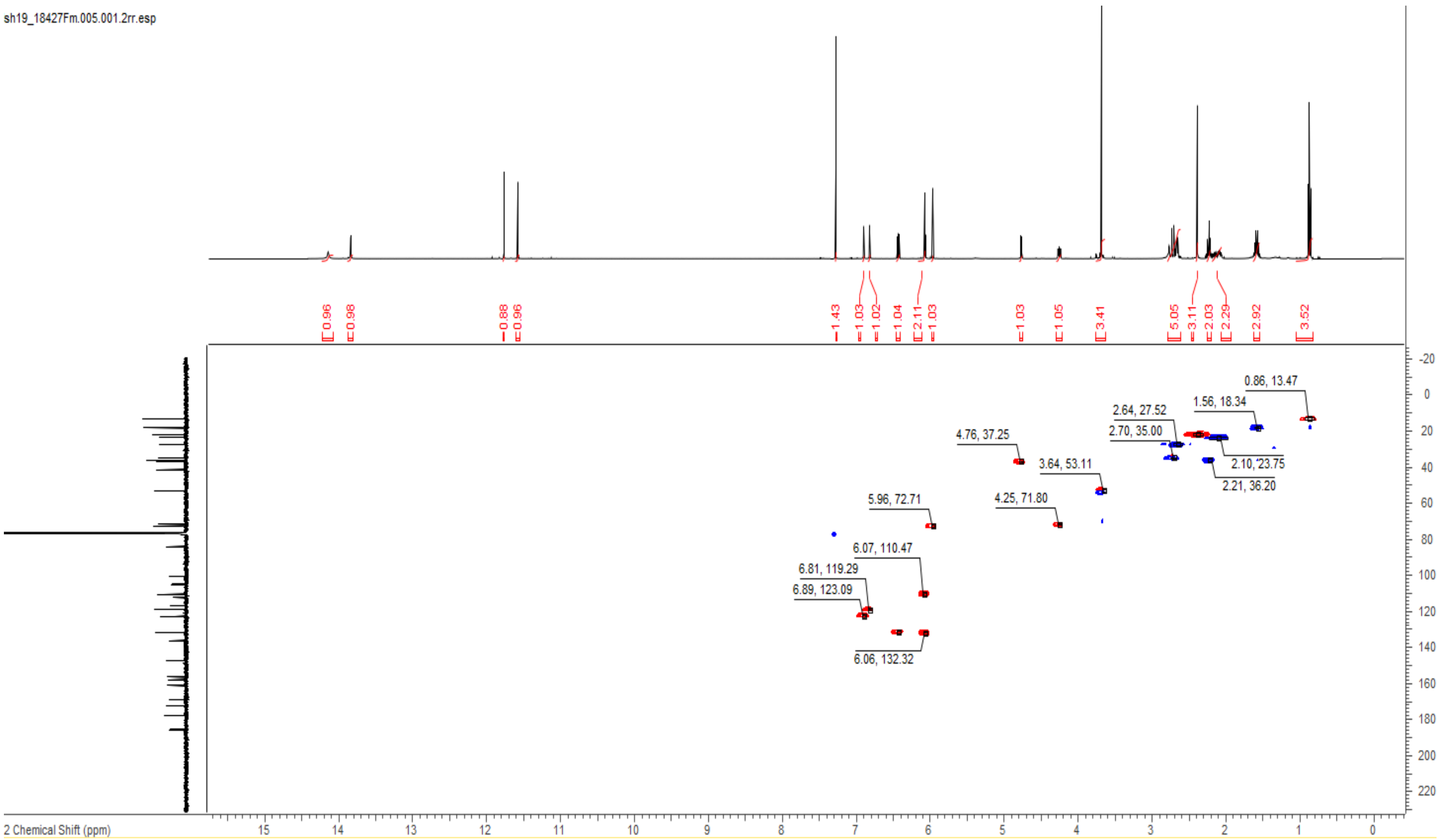

Figure S3. HSQC NMR spectrum (500 MHz, chloroform-*d*) of xanthoquinodin A11 (**1**)

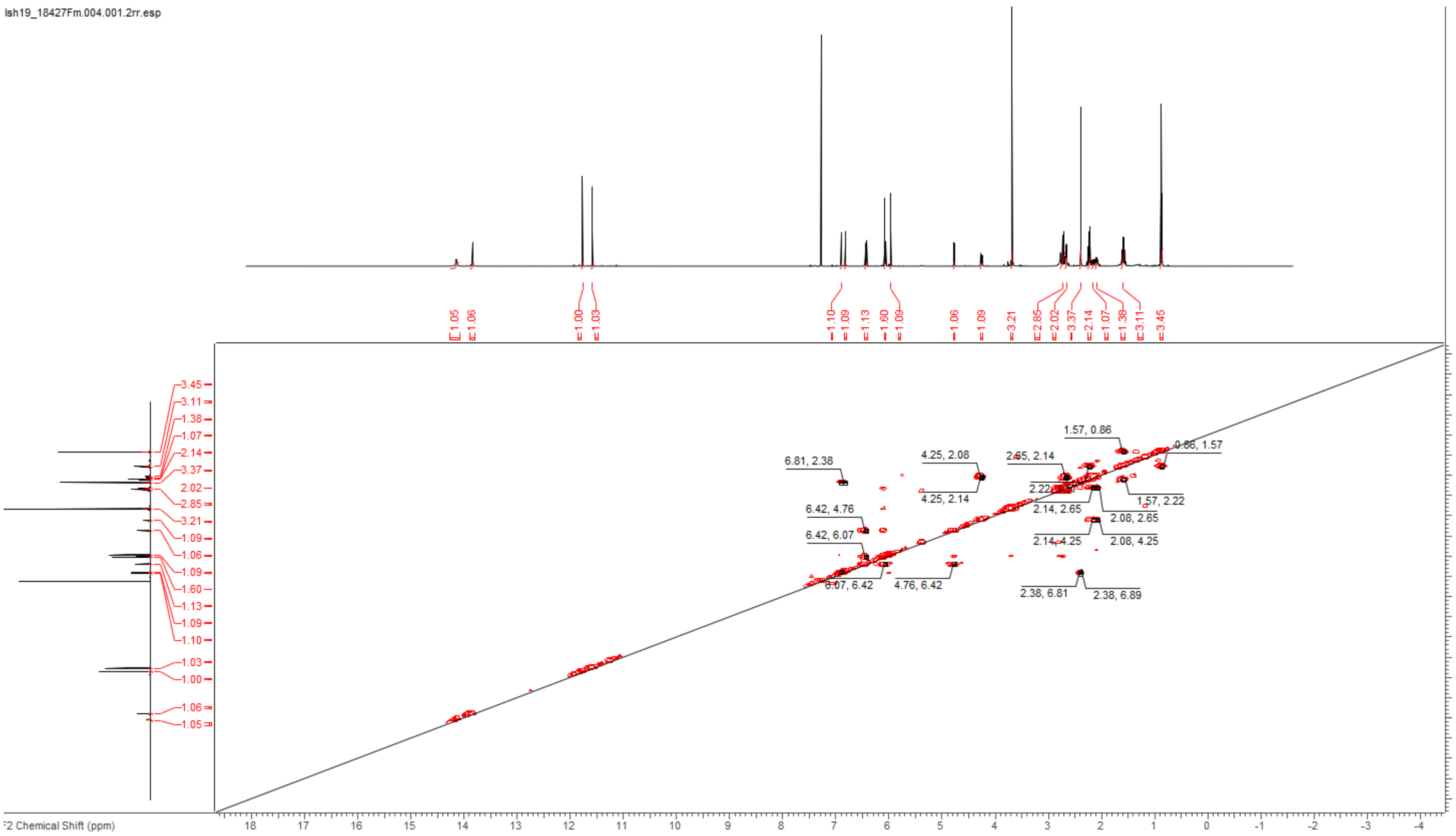

Figure S4. COSY NMR spectrum (500 MHz, chloroform-*d*) of xanthoquinodin A11 (**1**)

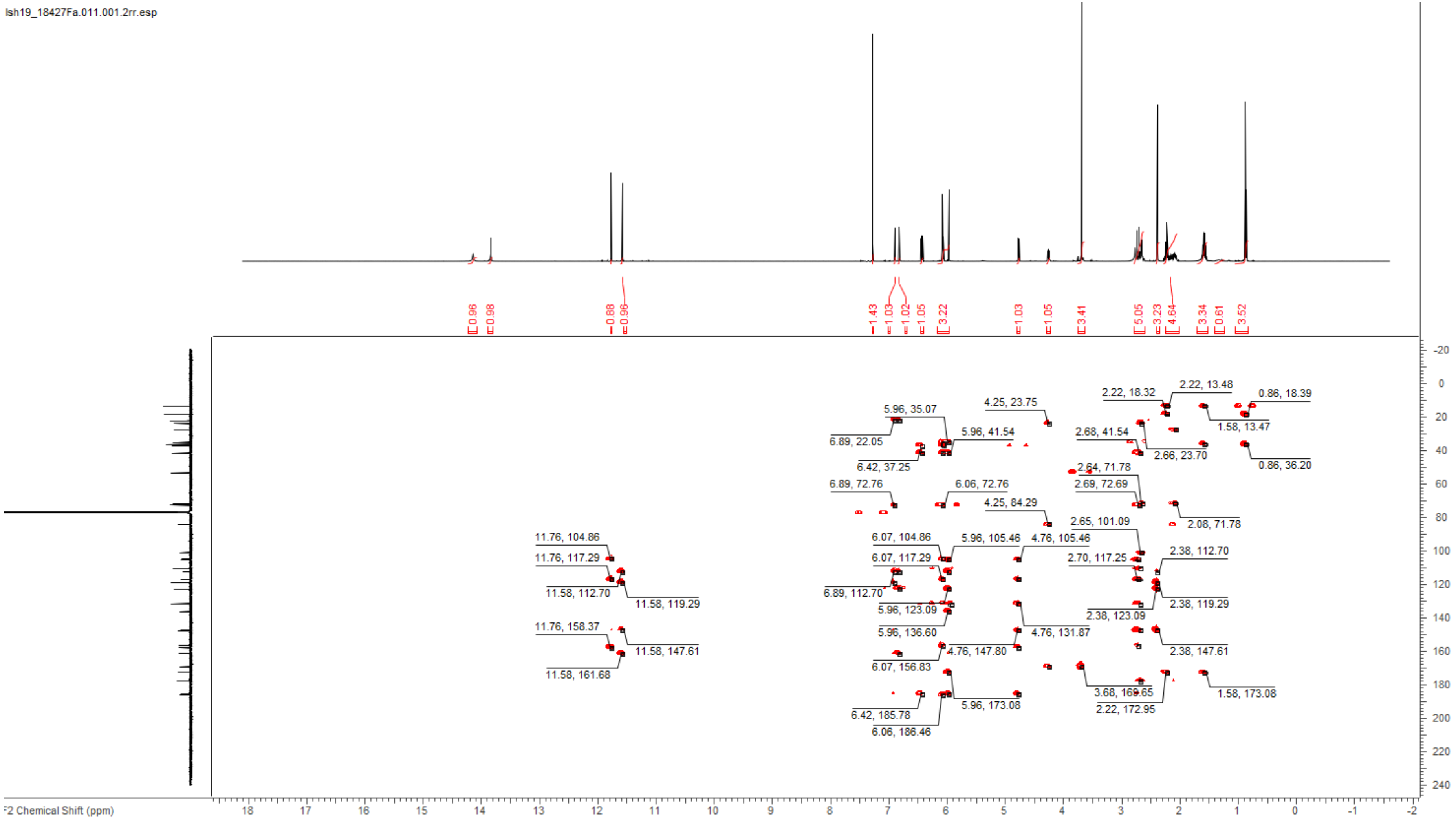

Figure S5. HMBC NMR spectrum (500 MHz, chloroform-*d*) of xanthoquinodin A11 (**1**)

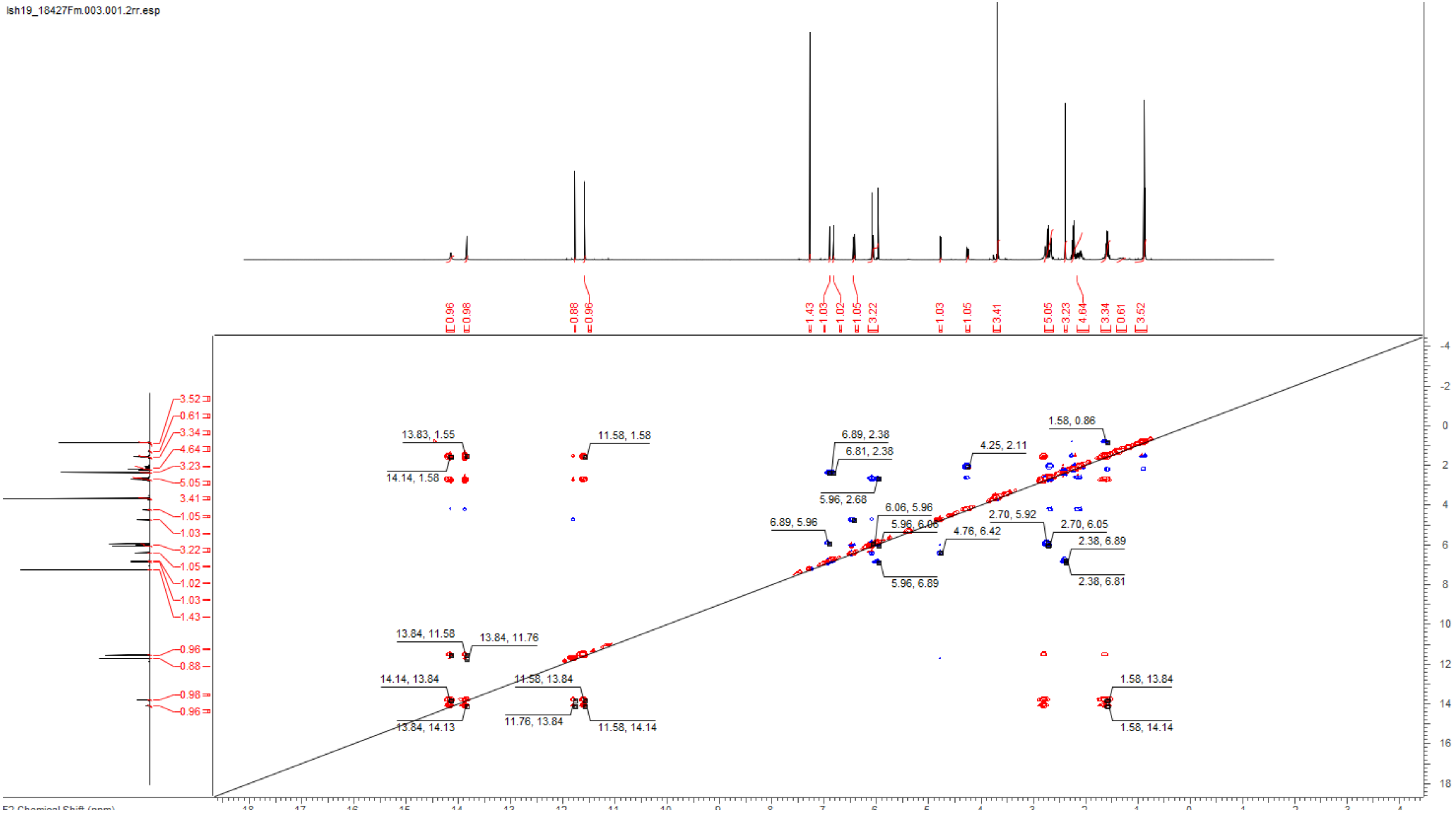

Figure S6. NOESY NMR spectrum (500 MHz, chloroform-*d*) of xanthoquinodin A11 (**1**)

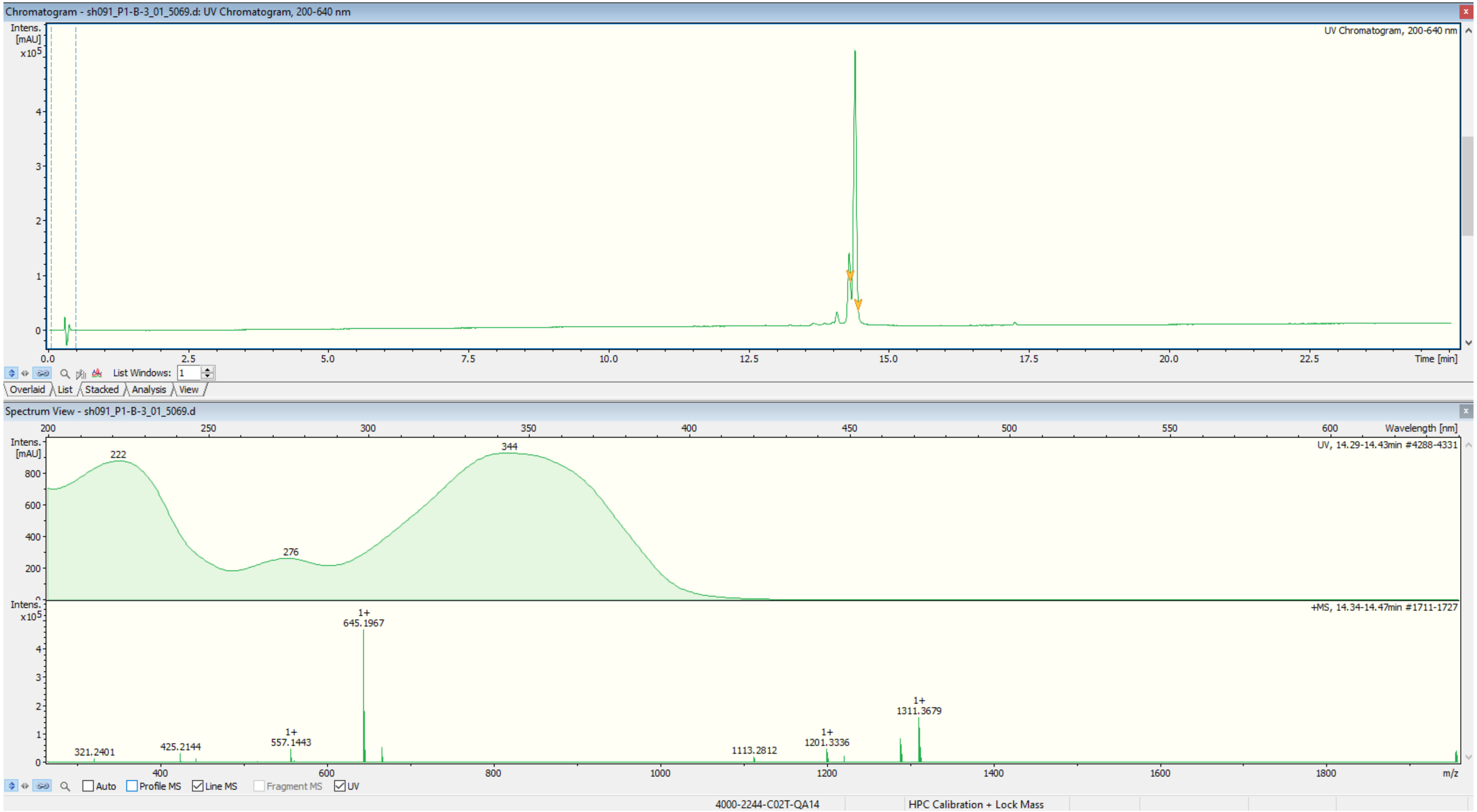

Figure S7. HRESIMS data of xanthoquinodin A11 (**1**)

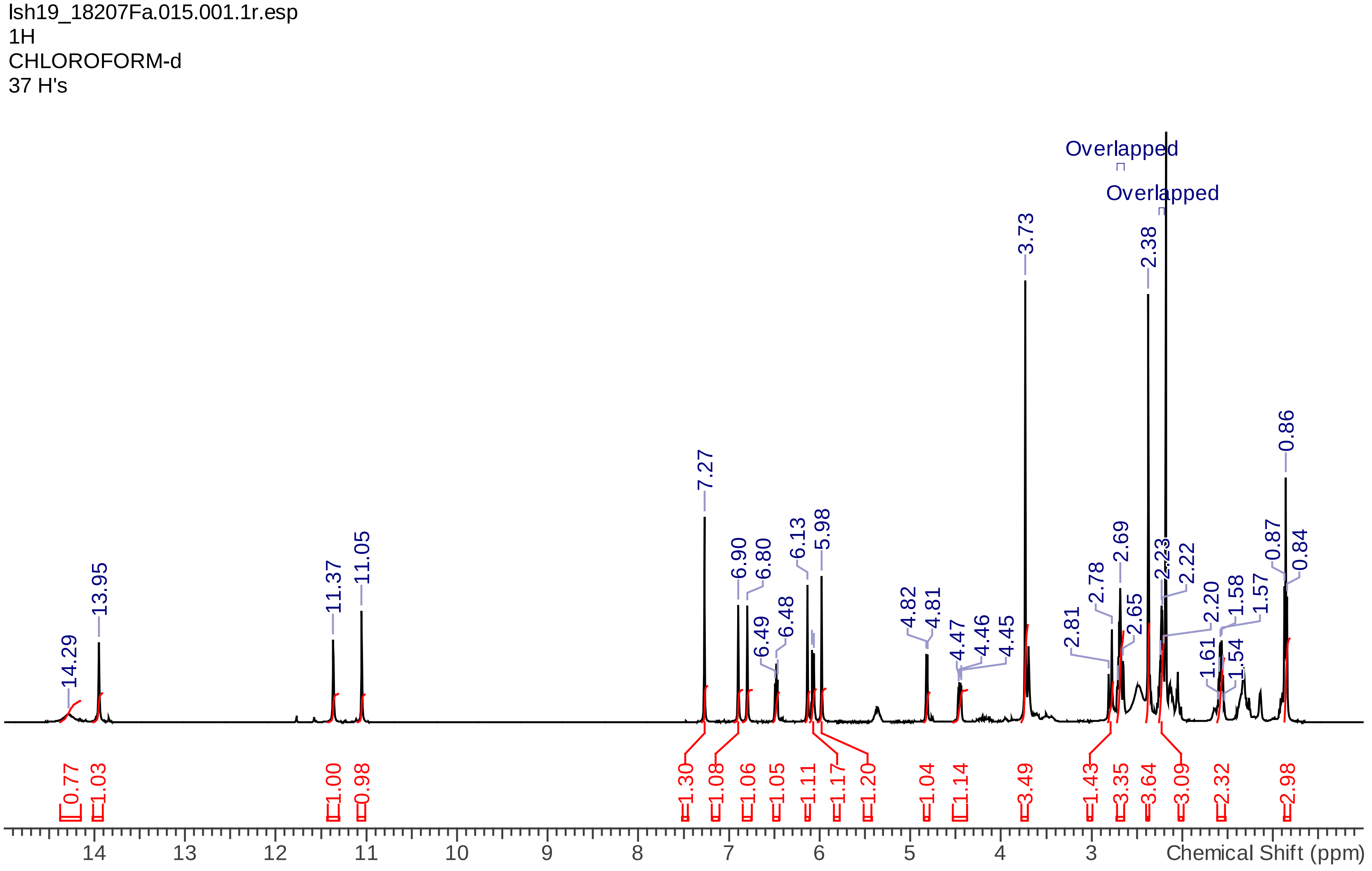

Figure S8. ^{1}H NMR spectrum (500 MHz, chloroform-*d*) of xanthoquinodin B10 (**2**)

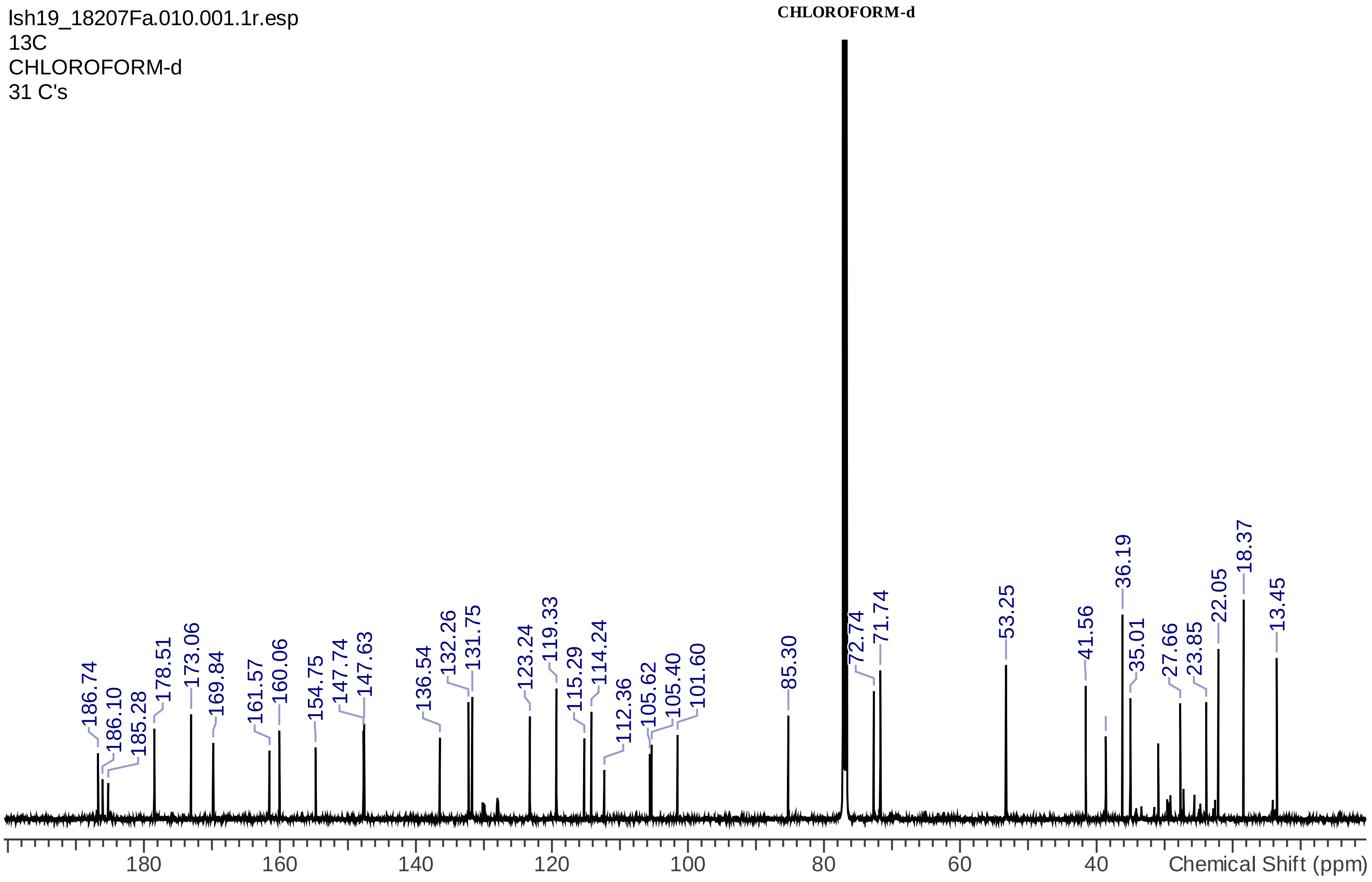

Figure S9. ^{13}C NMR spectrum (125 MHz, chloroform-*d*) of xanthoquinodin B10 (**2**)

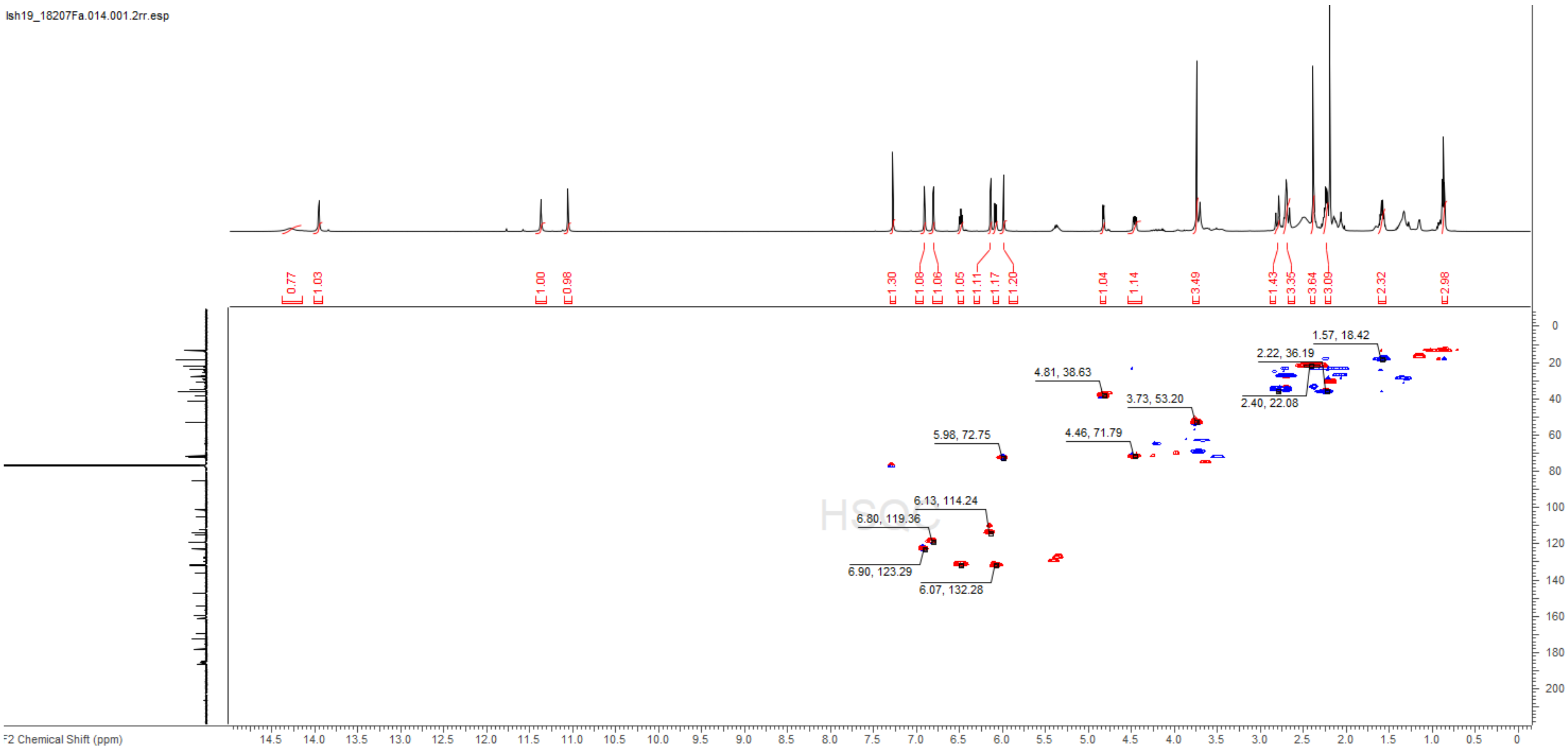

Figure S10. HSQC NMR spectrum (500 MHz, chloroform-*d*) of xanthoquinodin B10 (**2**)

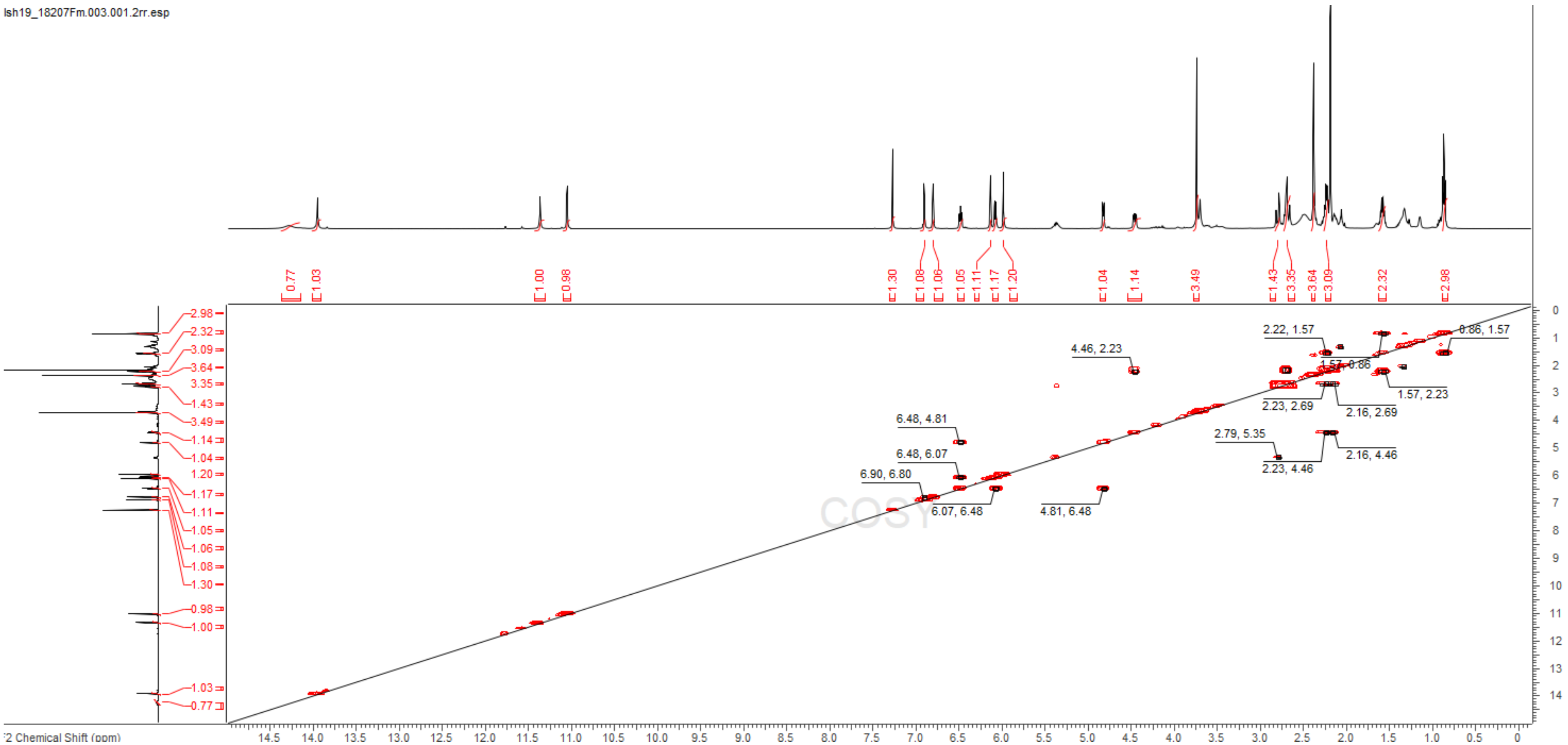

Figure S11. COSY NMR spectrum (500 MHz, chloroform-*d*) of xanthoquinodin B10 (**2**)

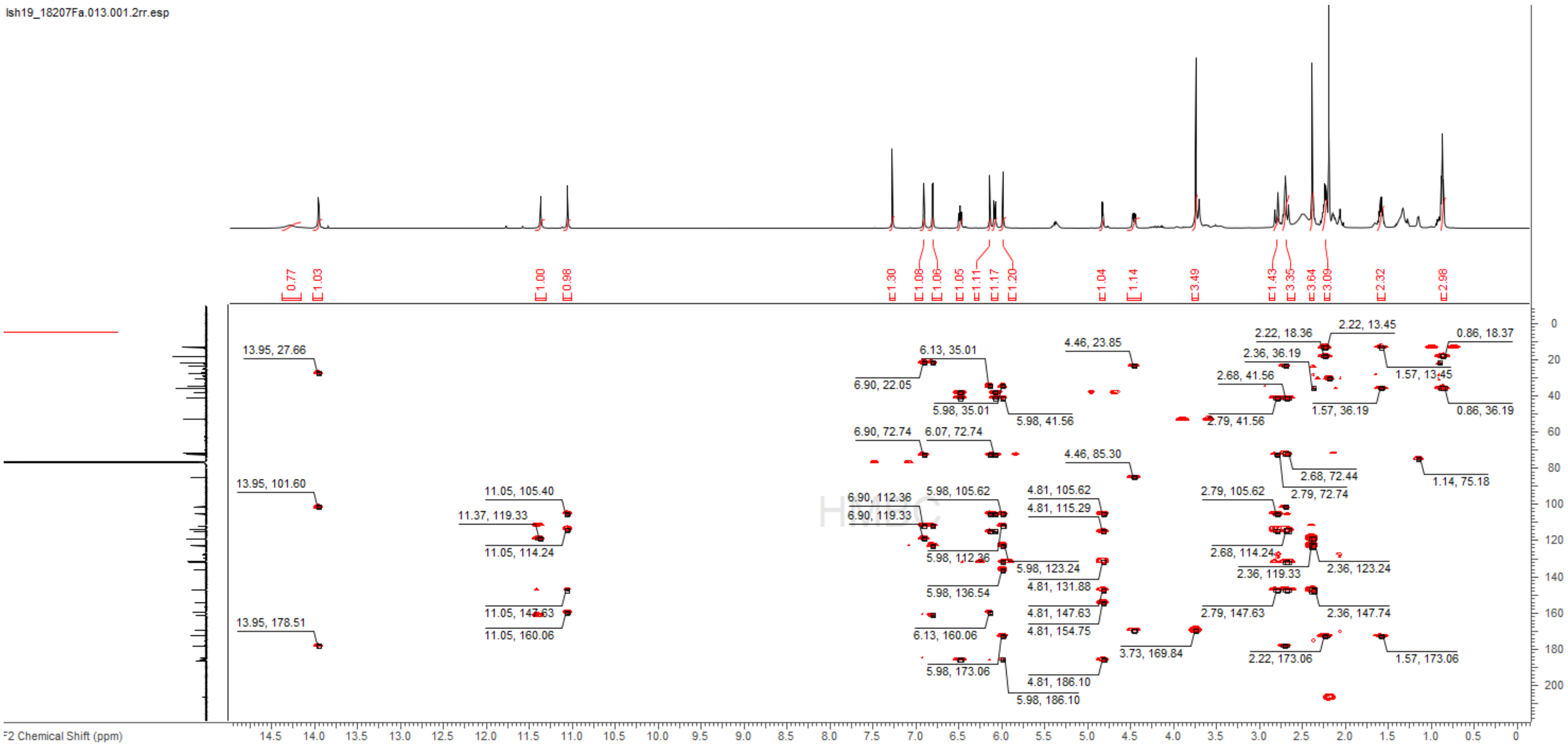

Figure S12. HMBC NMR spectrum (500 MHz, chloroform-*d*) of xanthoquinodin B10 (**2**)

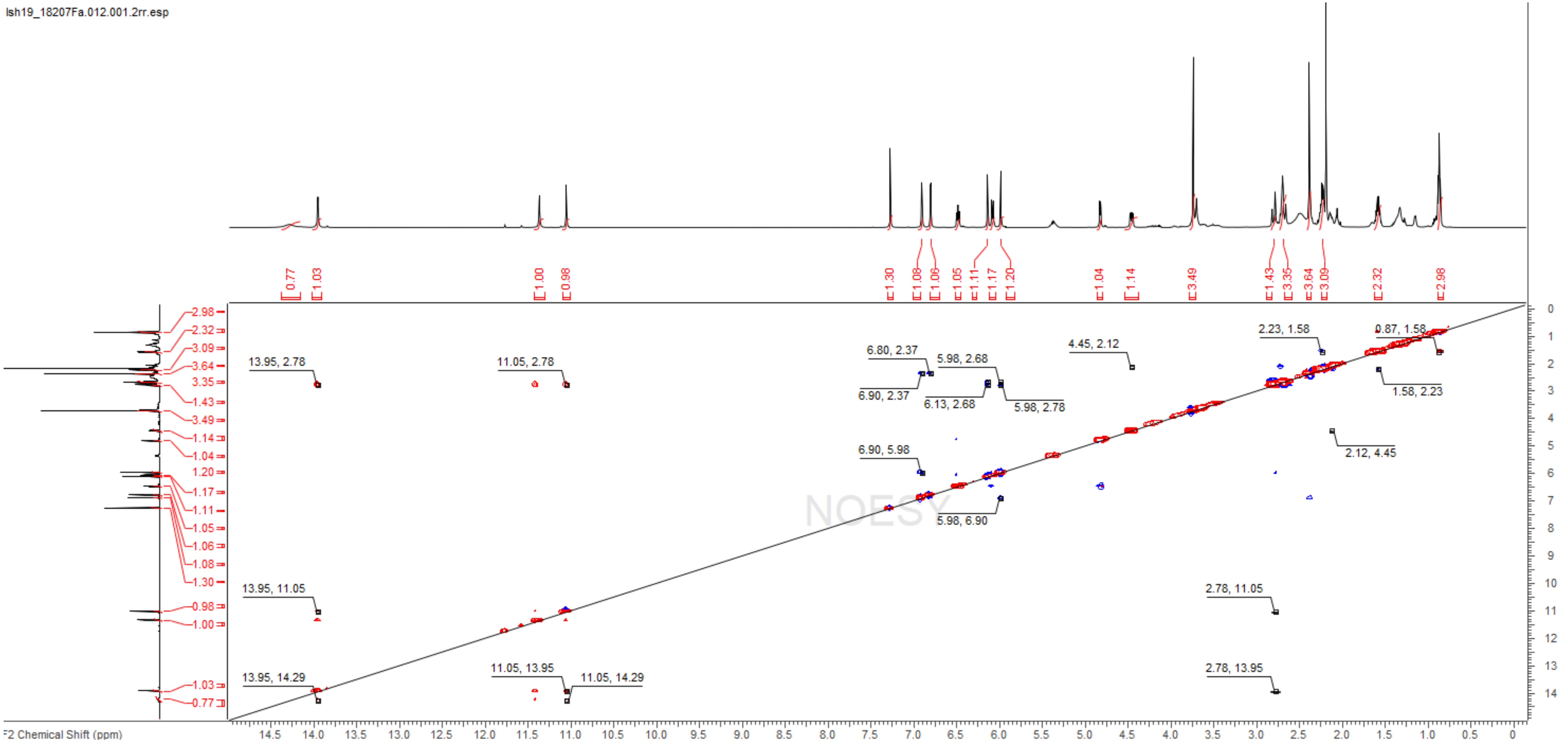

Figure S13. NOESY NMR spectrum (500 MHz, chloroform-*d*) of xanthoquinodin B10 (**2**)

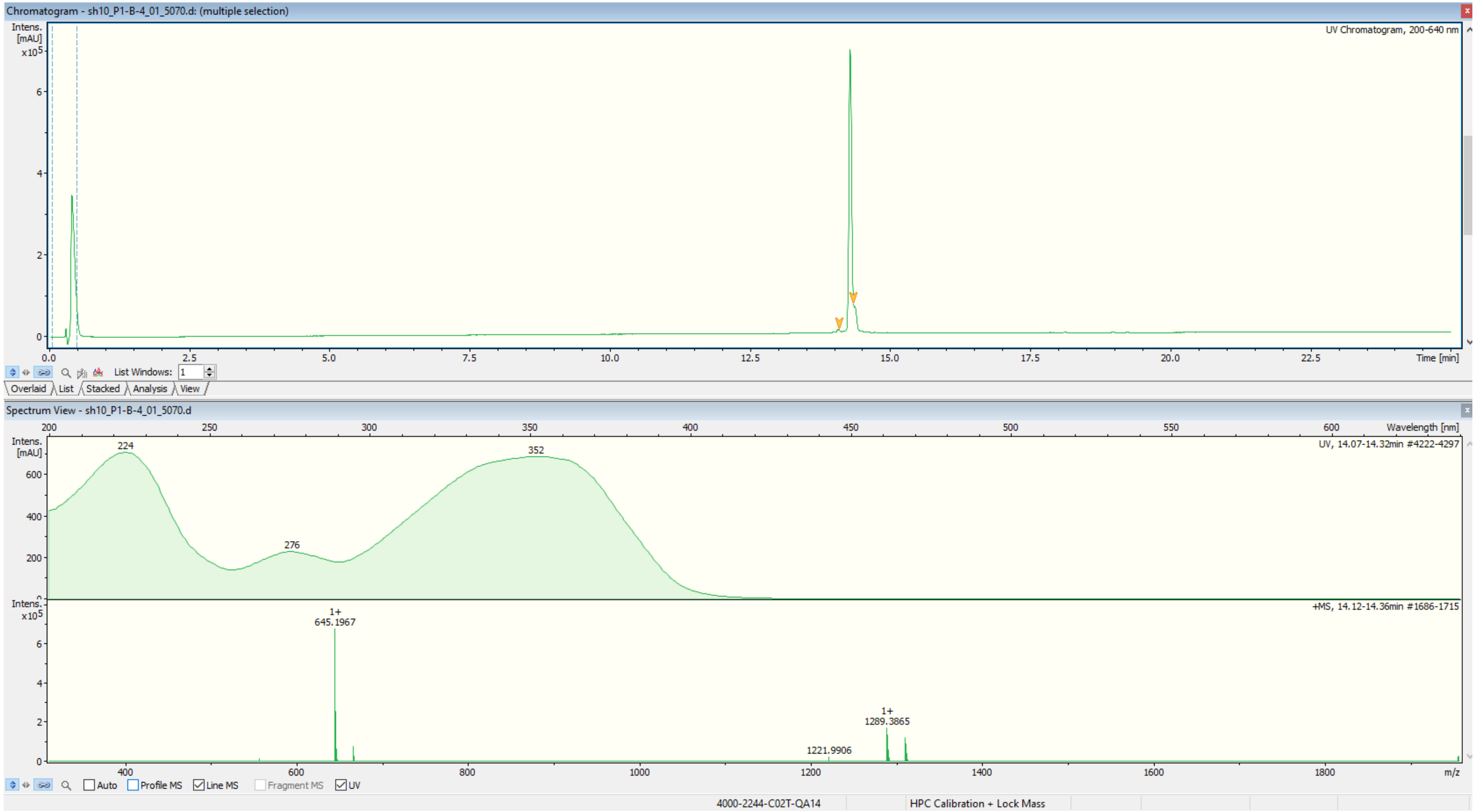

Figure S14. HRESIMS data of xanthoquinodin B10 (**2**)

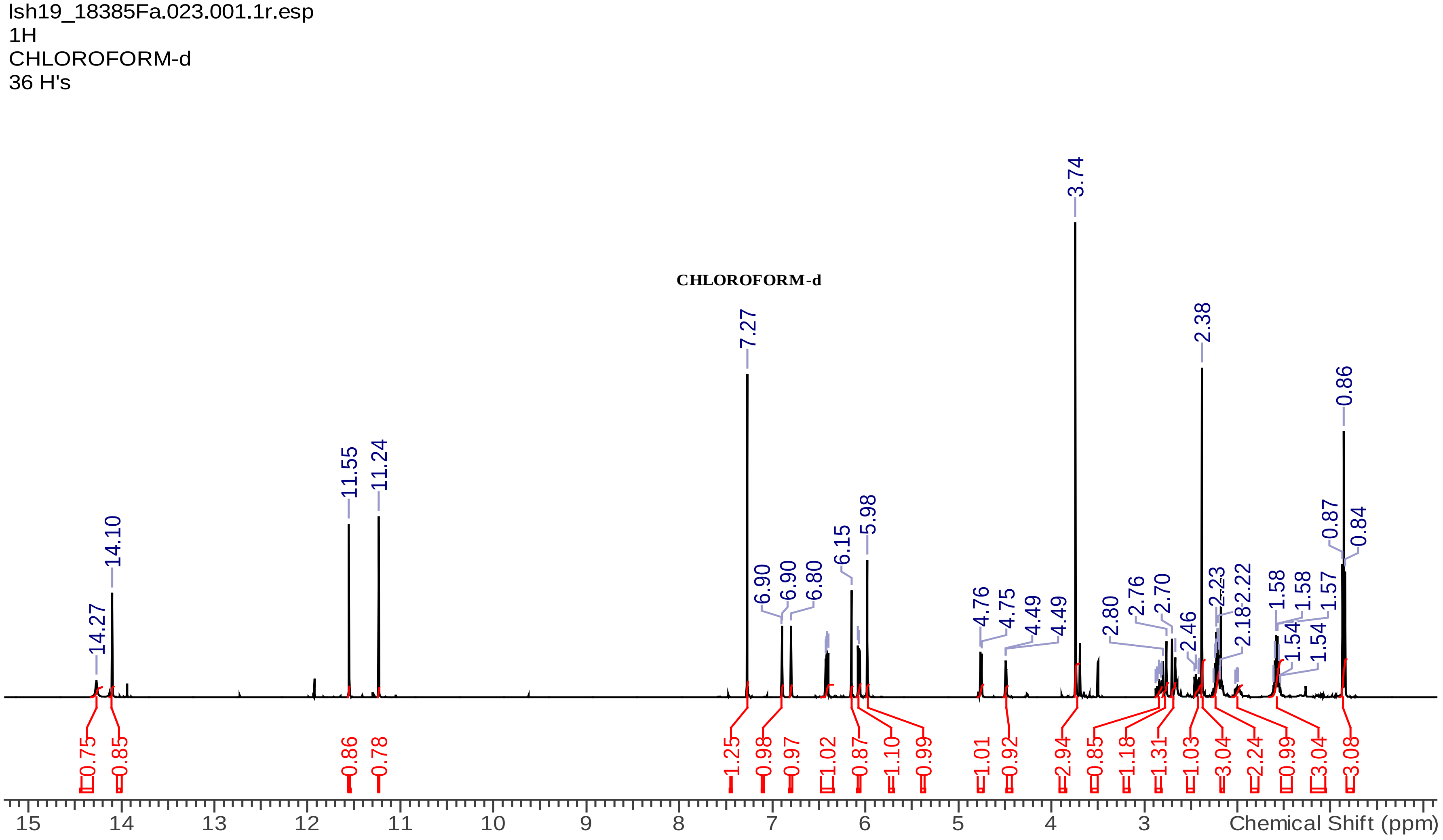

Figure S15. ^{1}H NMR spectrum (500 MHz, chloroform-*d*) of xanthoquinodin B11 (**3**)

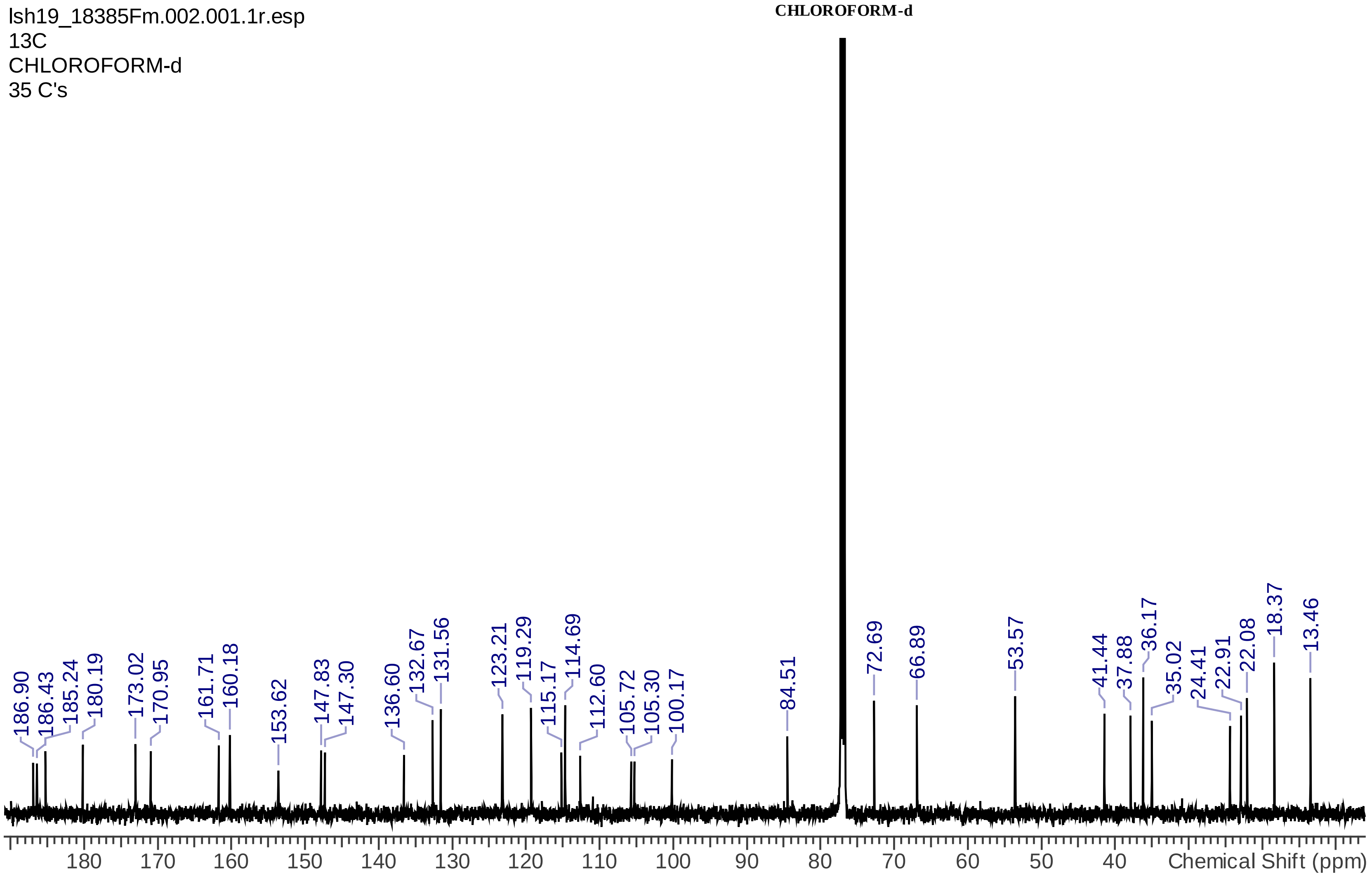

Figure S16. ^{13}C NMR spectrum (125 MHz, chloroform-*d*) of xanthoquinodin B11 (**3**)

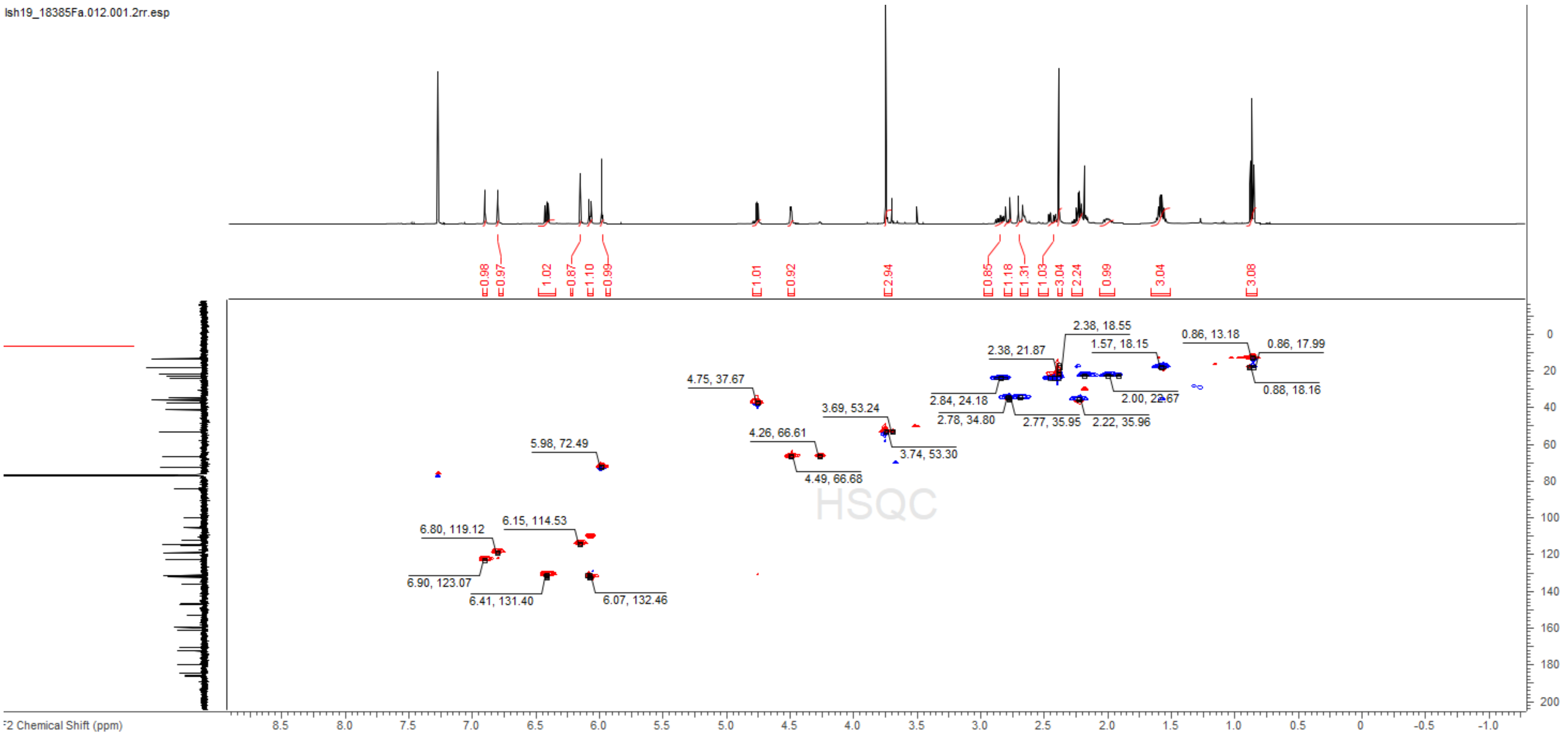

Figure S17. HSQC NMR spectrum (500 MHz, chloroform-*d*) of xanthoquinodin B11 (**3**)

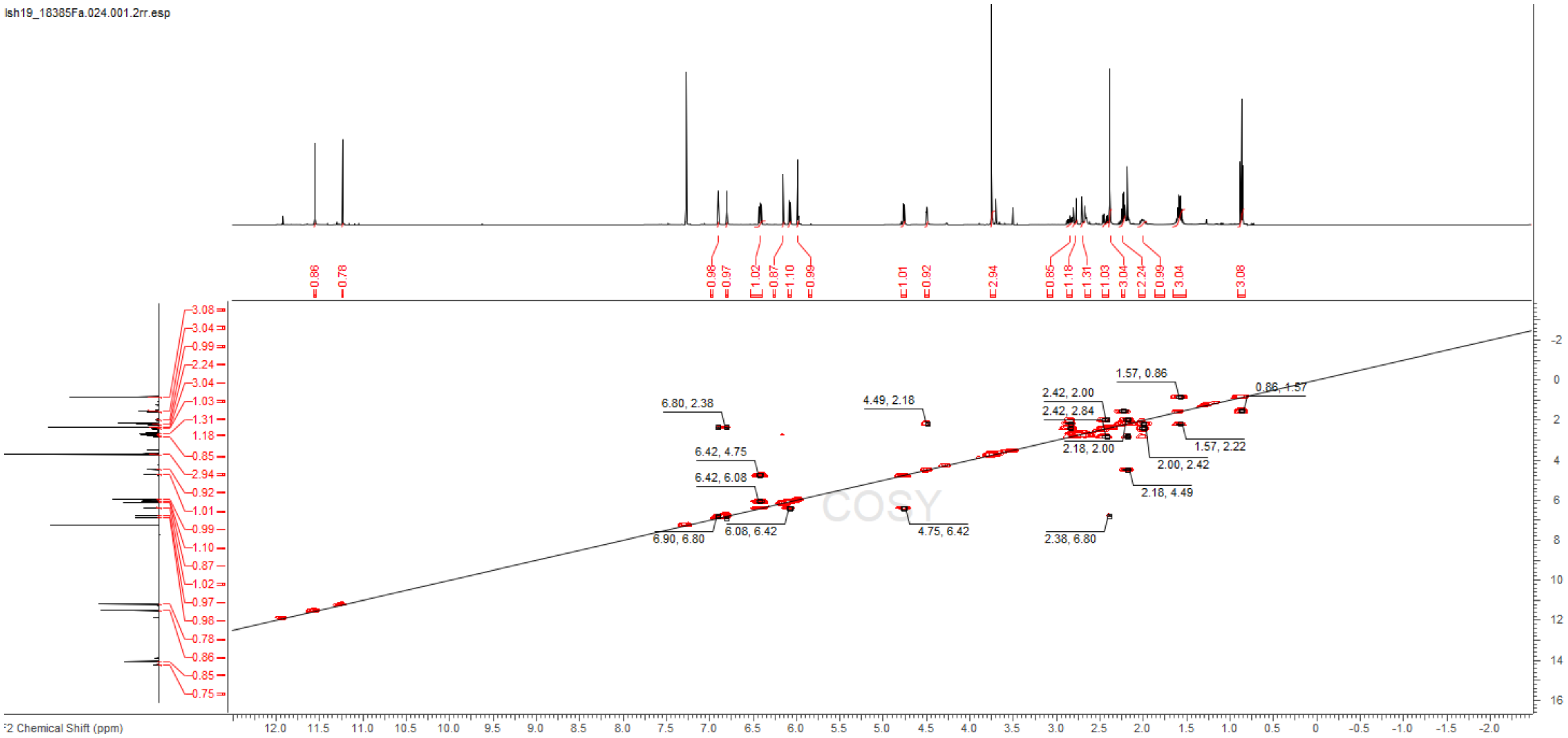

Figure S18. COSY NMR spectrum (500 MHz, chloroform-*d*) of xanthoquinodin B11 (**3**)

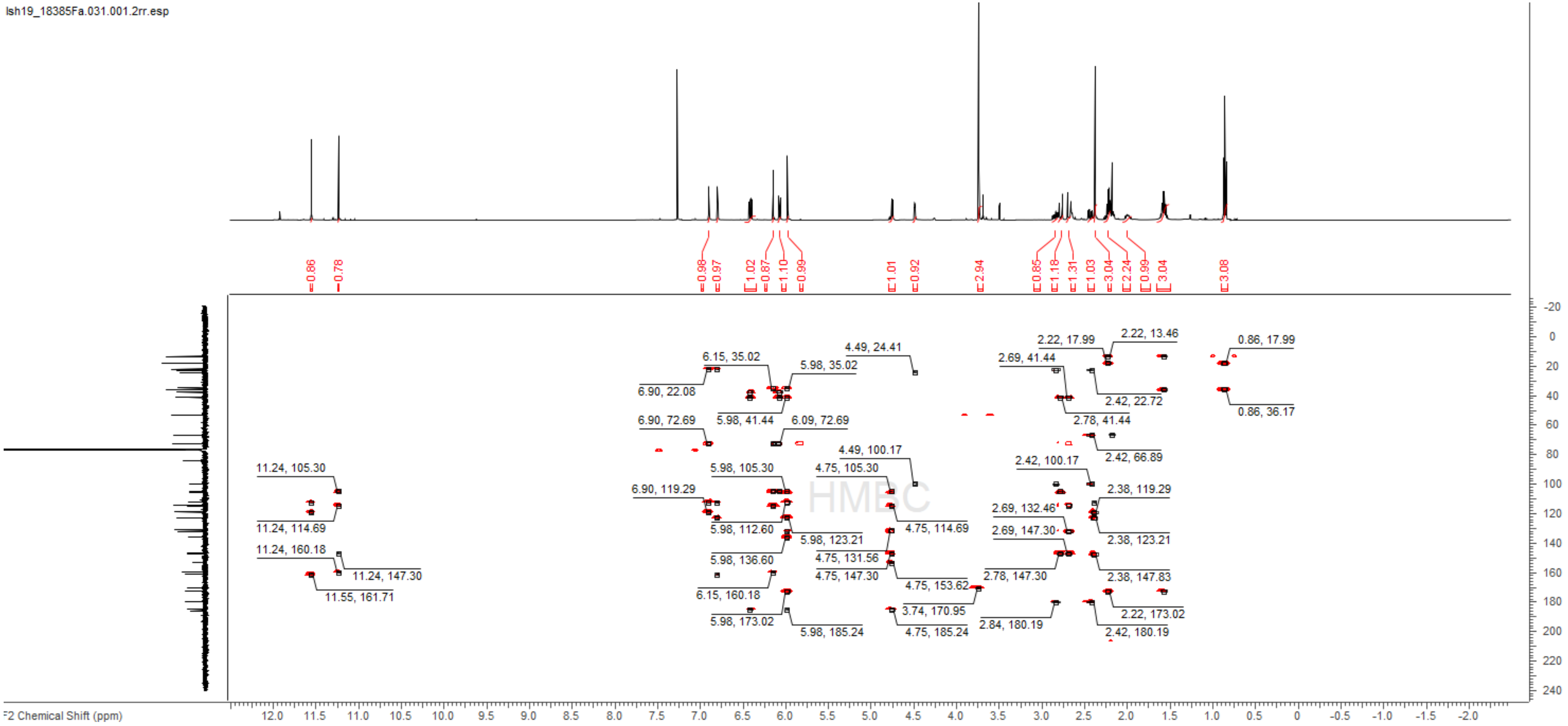

Figure S19. HMBC NMR spectrum (500 MHz, chloroform-*d*) of xanthoquinodin B11 (**3**)

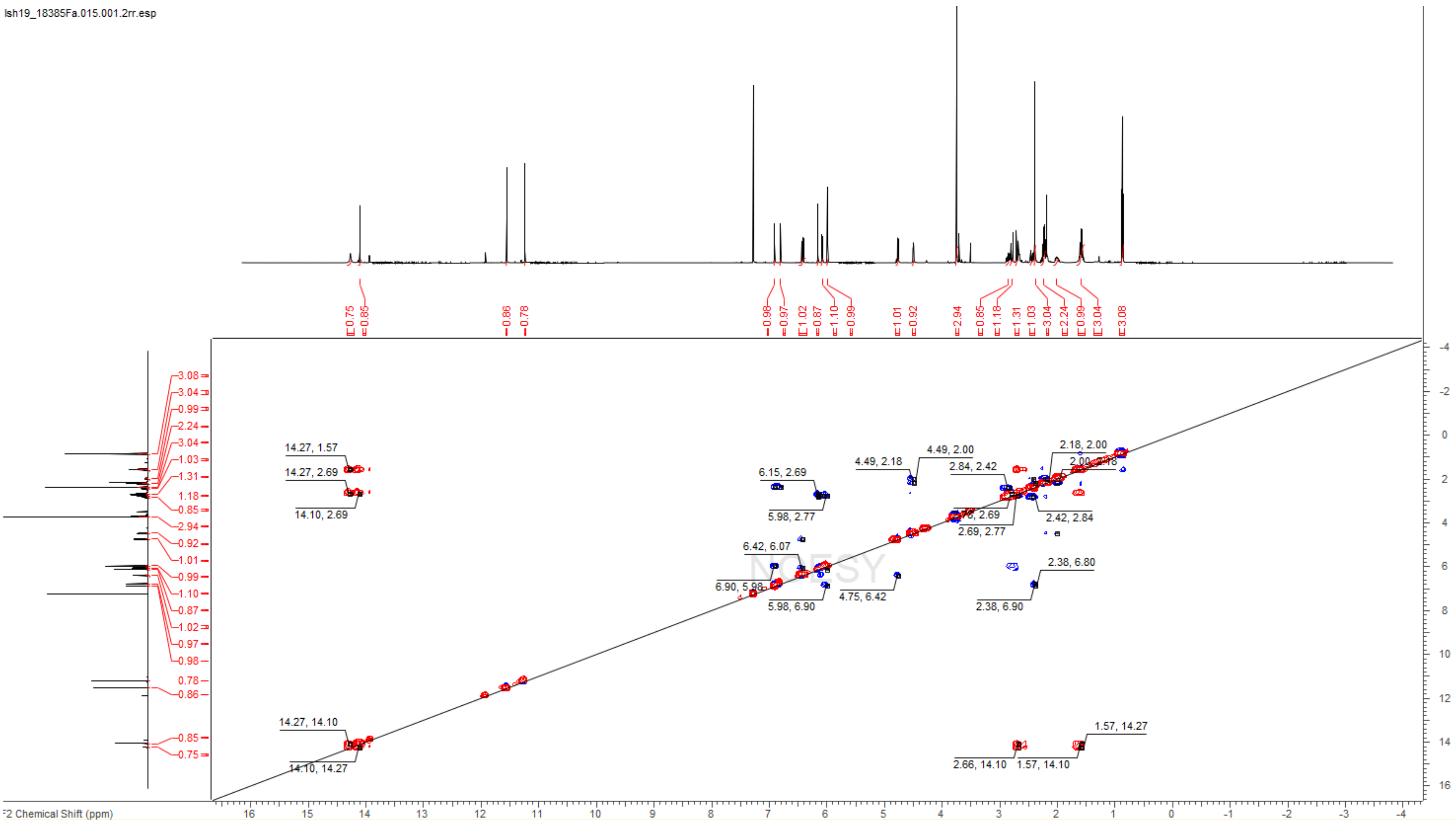

Figure S20. NOESY NMR spectrum (500 MHz, chloroform-*d*) of xanthoquinodin B11 (**3**)

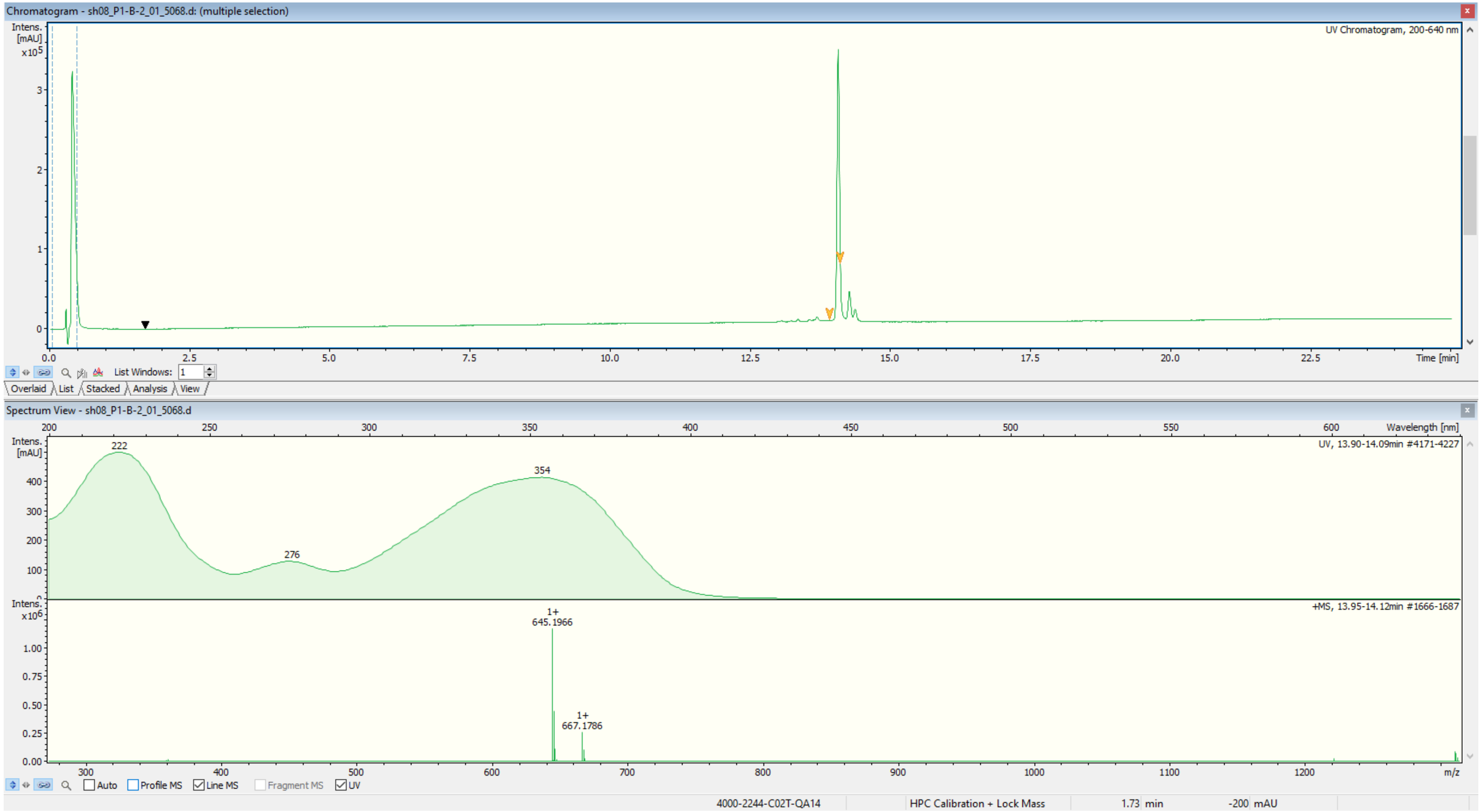

Figure S21. HRESIMS data of xanthoquinodin B11 (**3**)

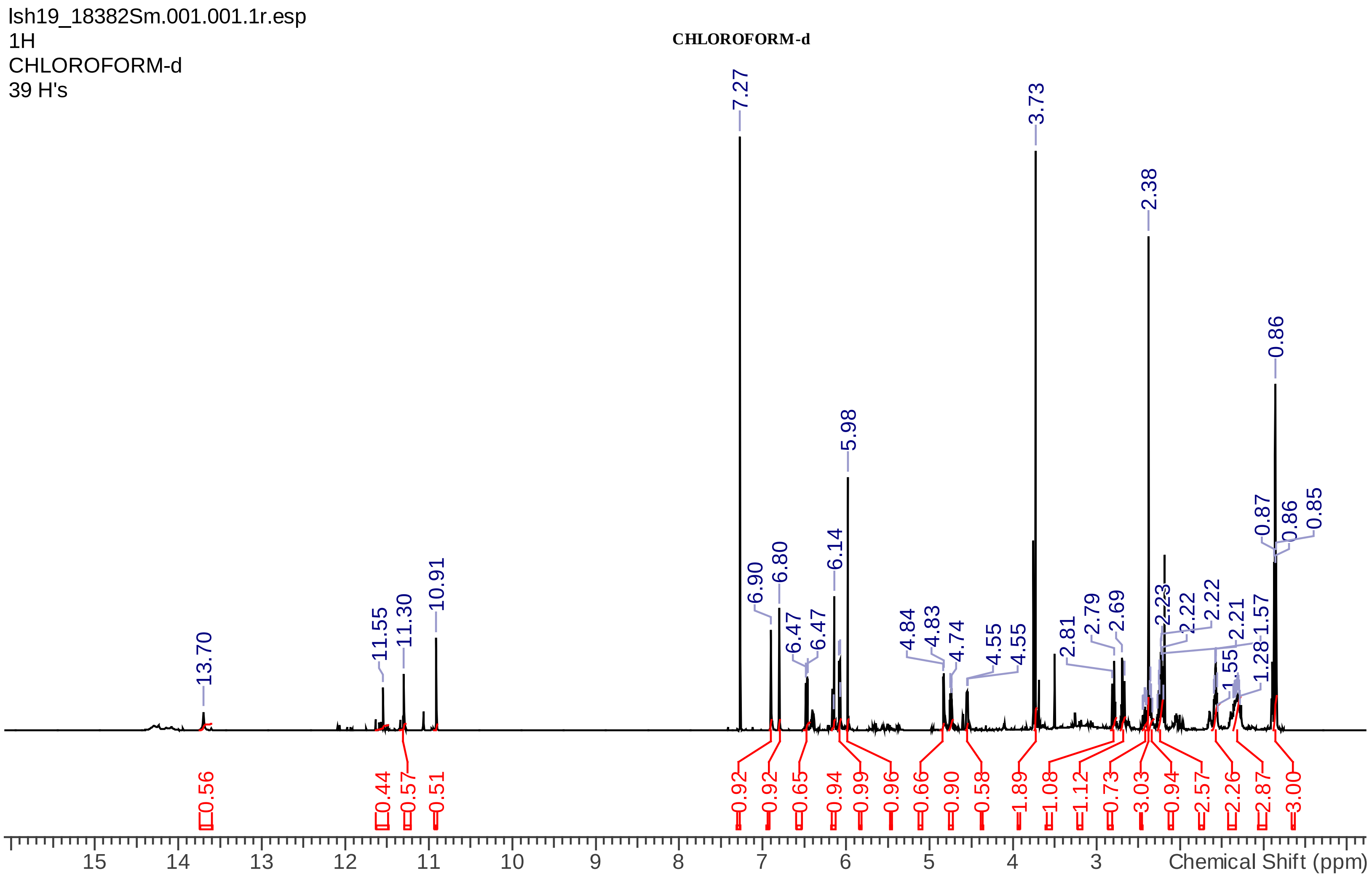

Figure S22. ^{1}H NMR spectrum (700 MHz, chloroform-*d*) of xanthoquinodin B12 (**4**)

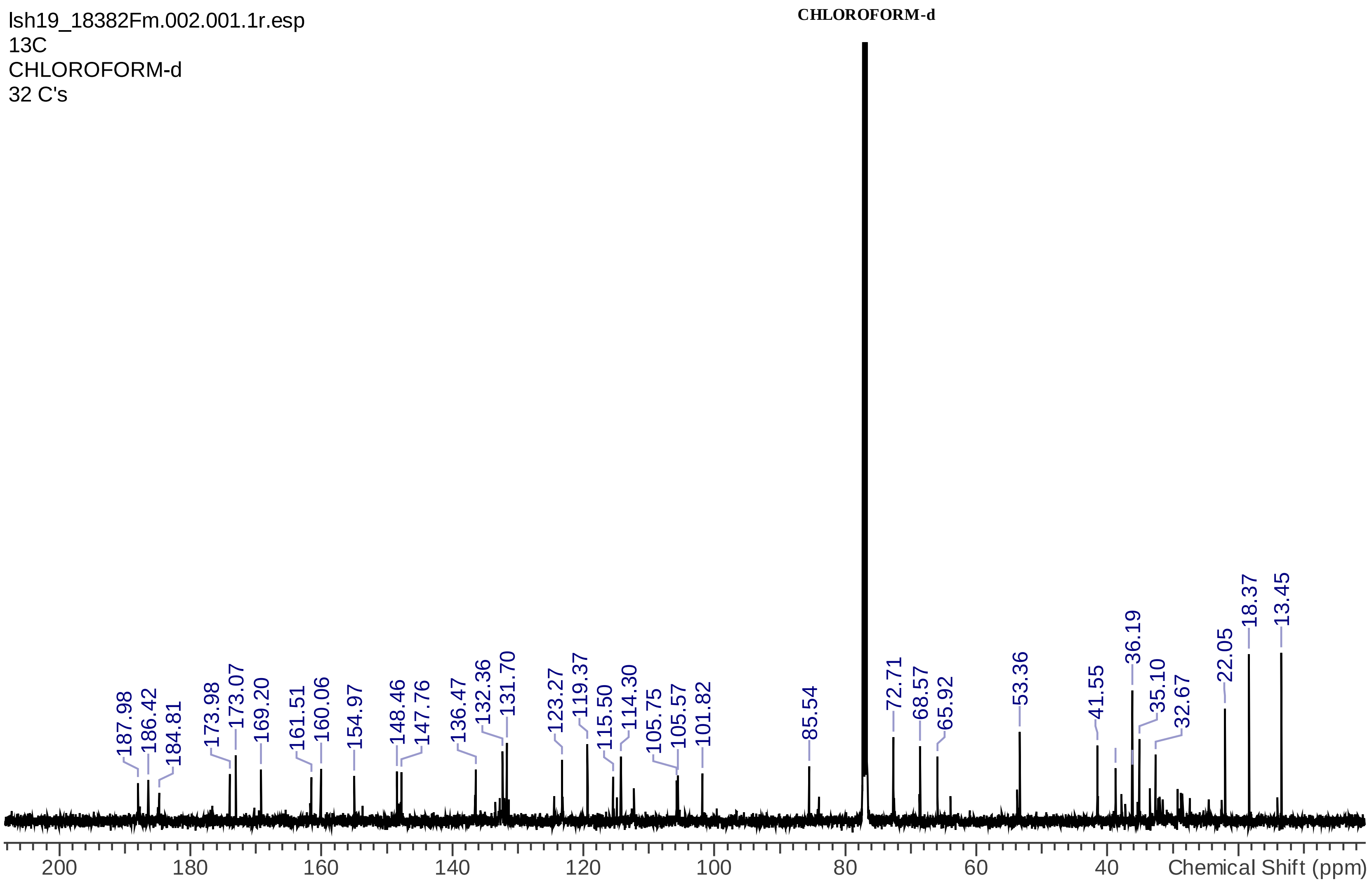

Figure S23. ^{13}C NMR spectrum (125 MHz, chloroform-*d*) of xanthoquinodin B12 (**4**)

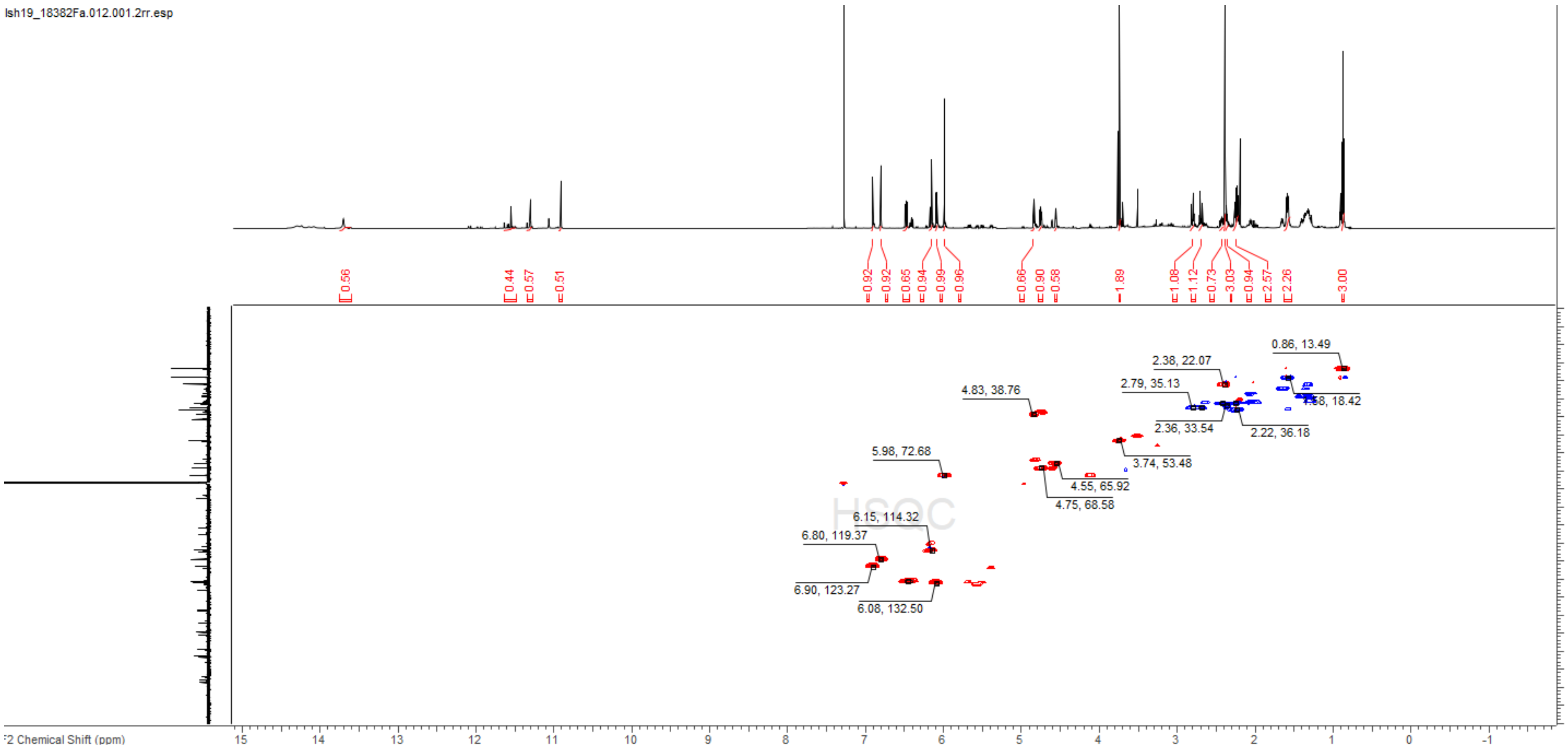

Figure S24. HSQC NMR spectrum (500 MHz, chloroform-*d*) of xanthoquinodin B12 (**4**)

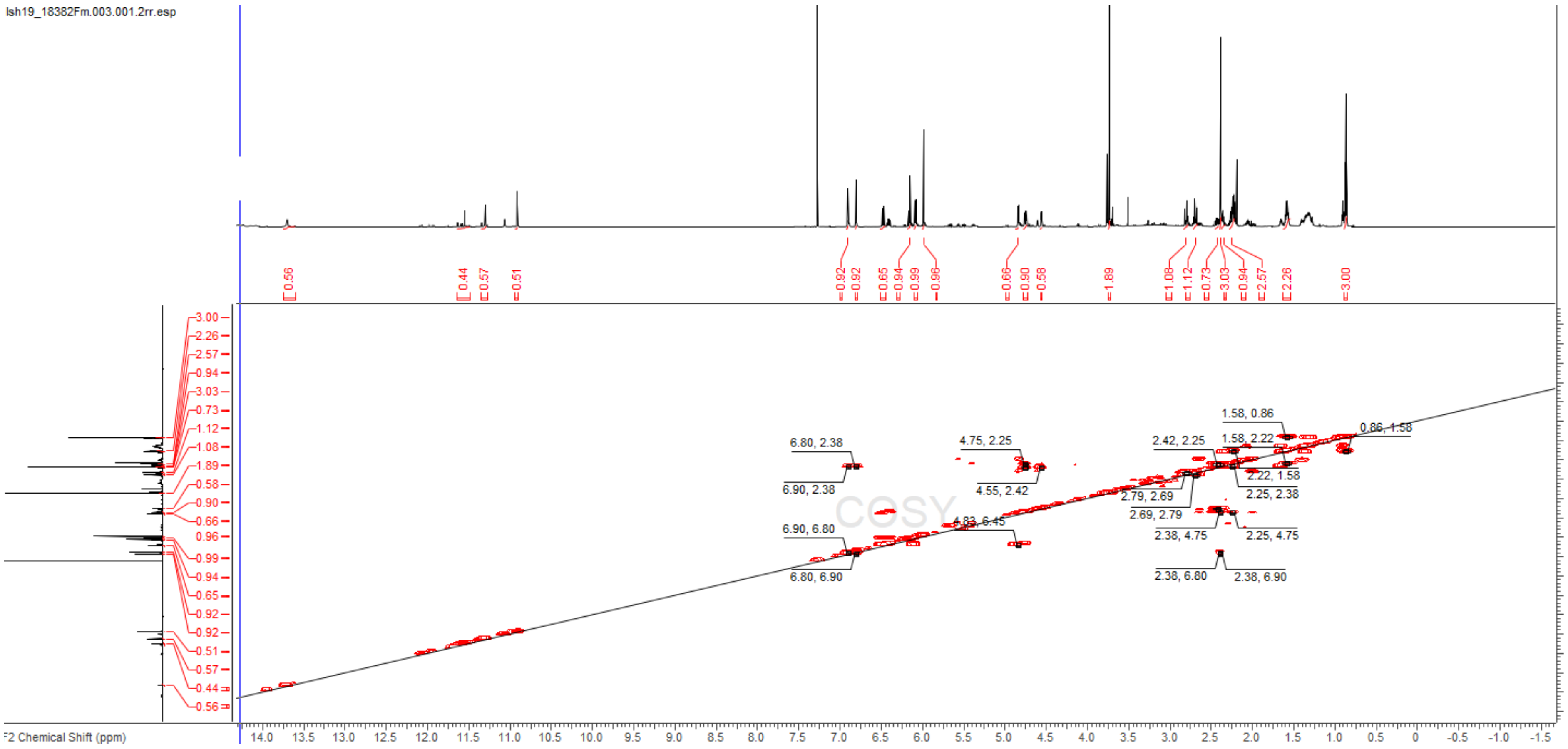

Figure S25. COSY NMR spectrum (500 MHz, chloroform-*d*) of xanthoquinodin B12 (**4**)

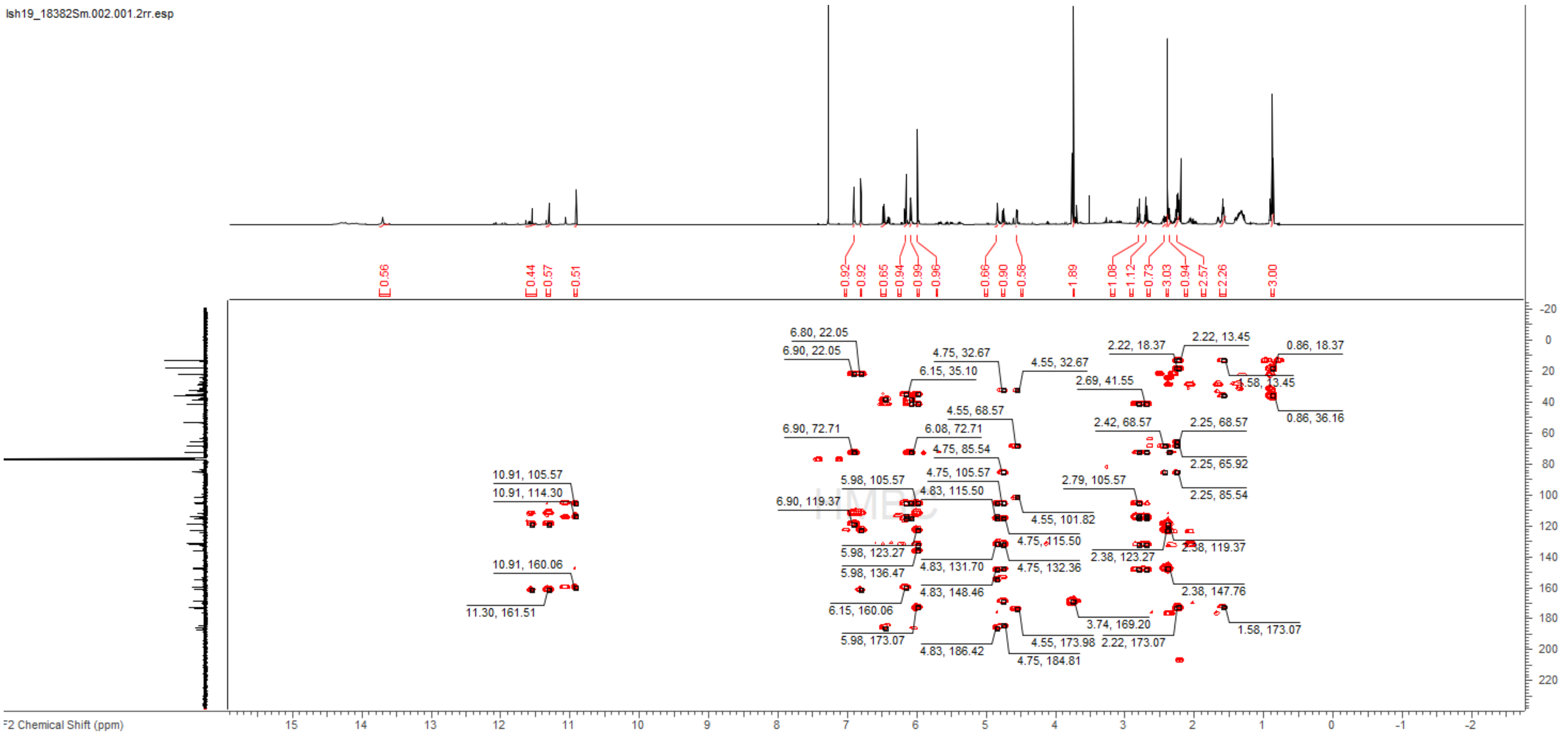

Figure S26. HMBC NMR spectrum (700 MHz, chloroform-*d*) of xanthoquinodin B12 (**4**)

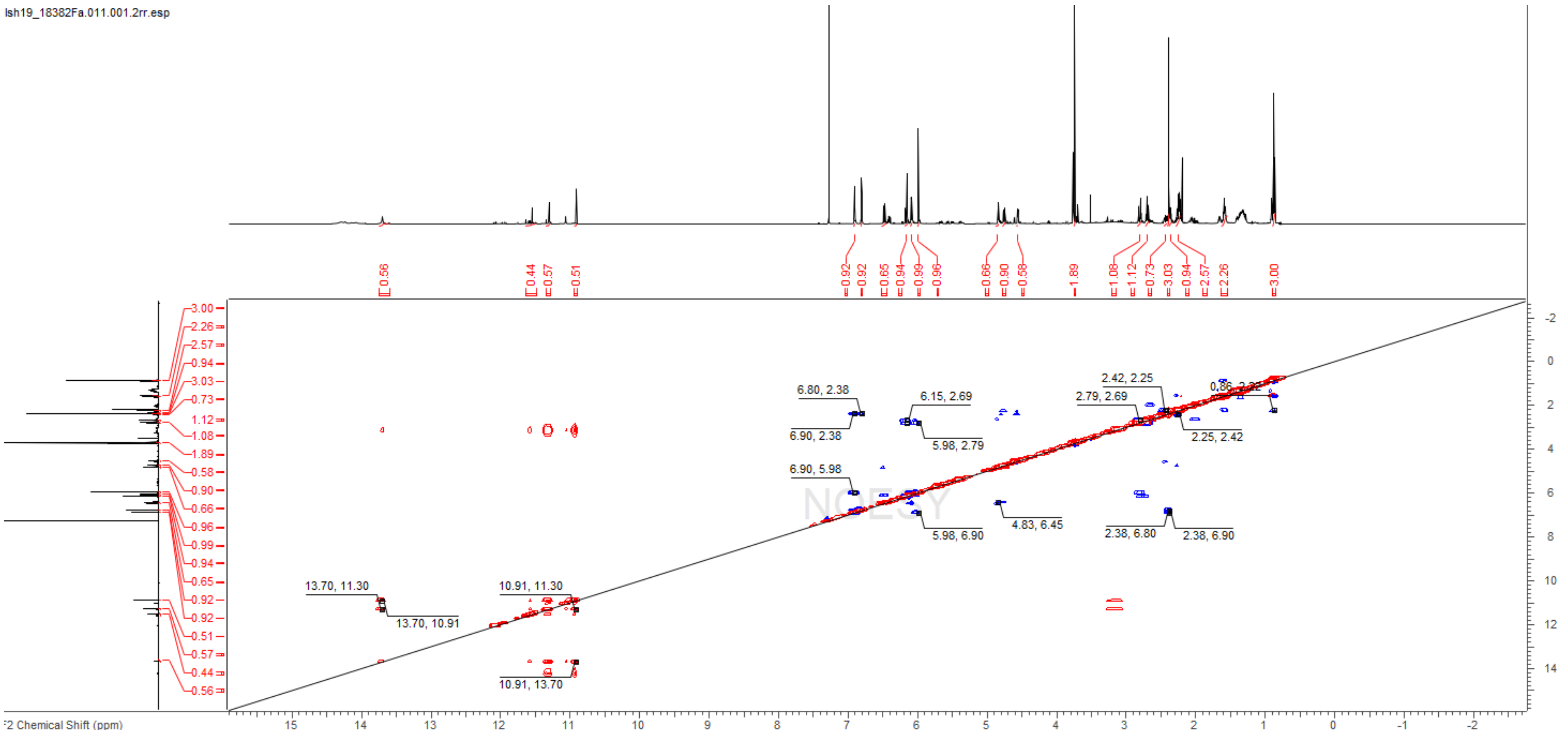

Figure S27. NOESY NMR spectrum (500 MHz, chloroform-*d*) of xanthoquinodin B12 (**4**)

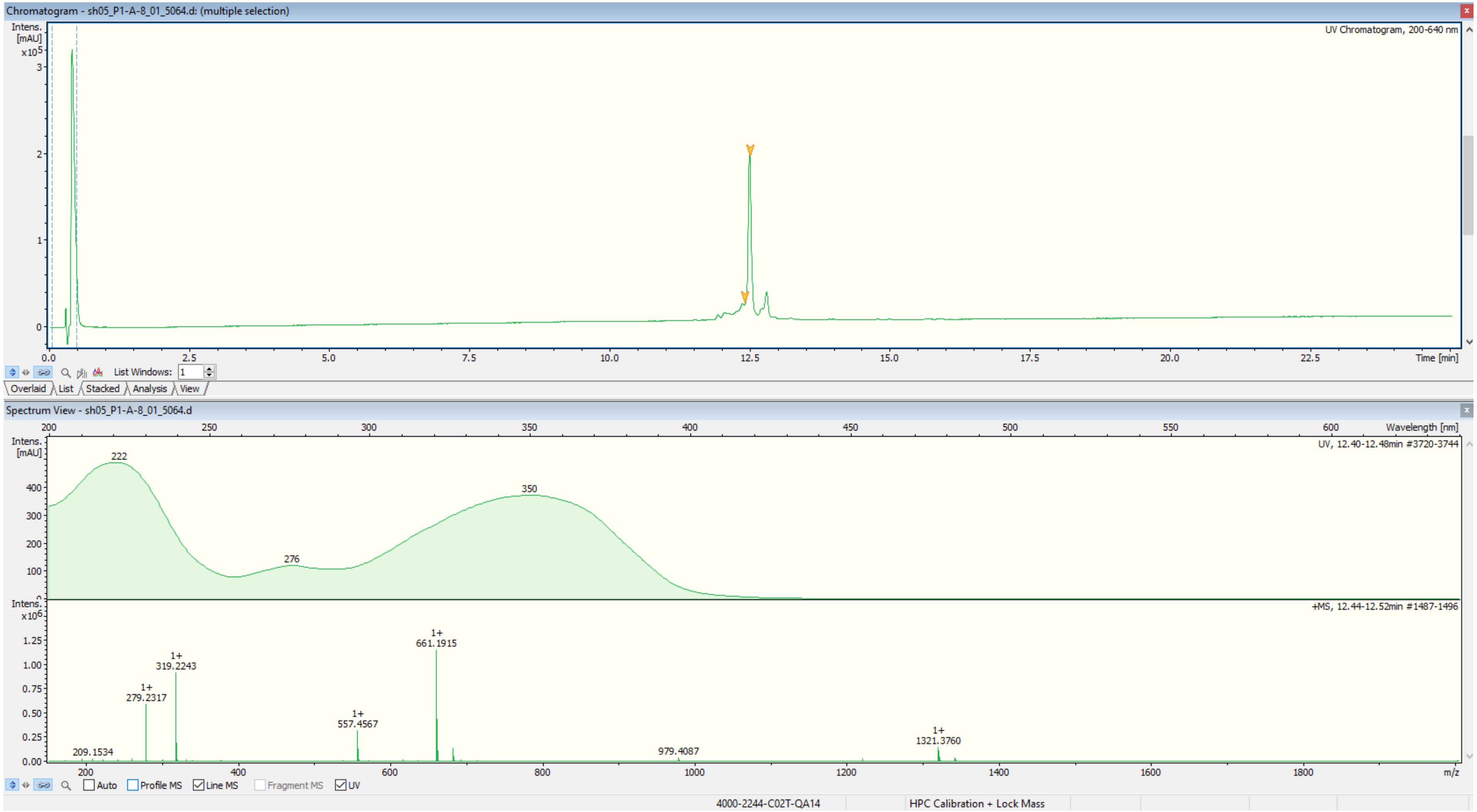

Figure S28. HRESIMS data of xanthoquinodin B12 (**4**)

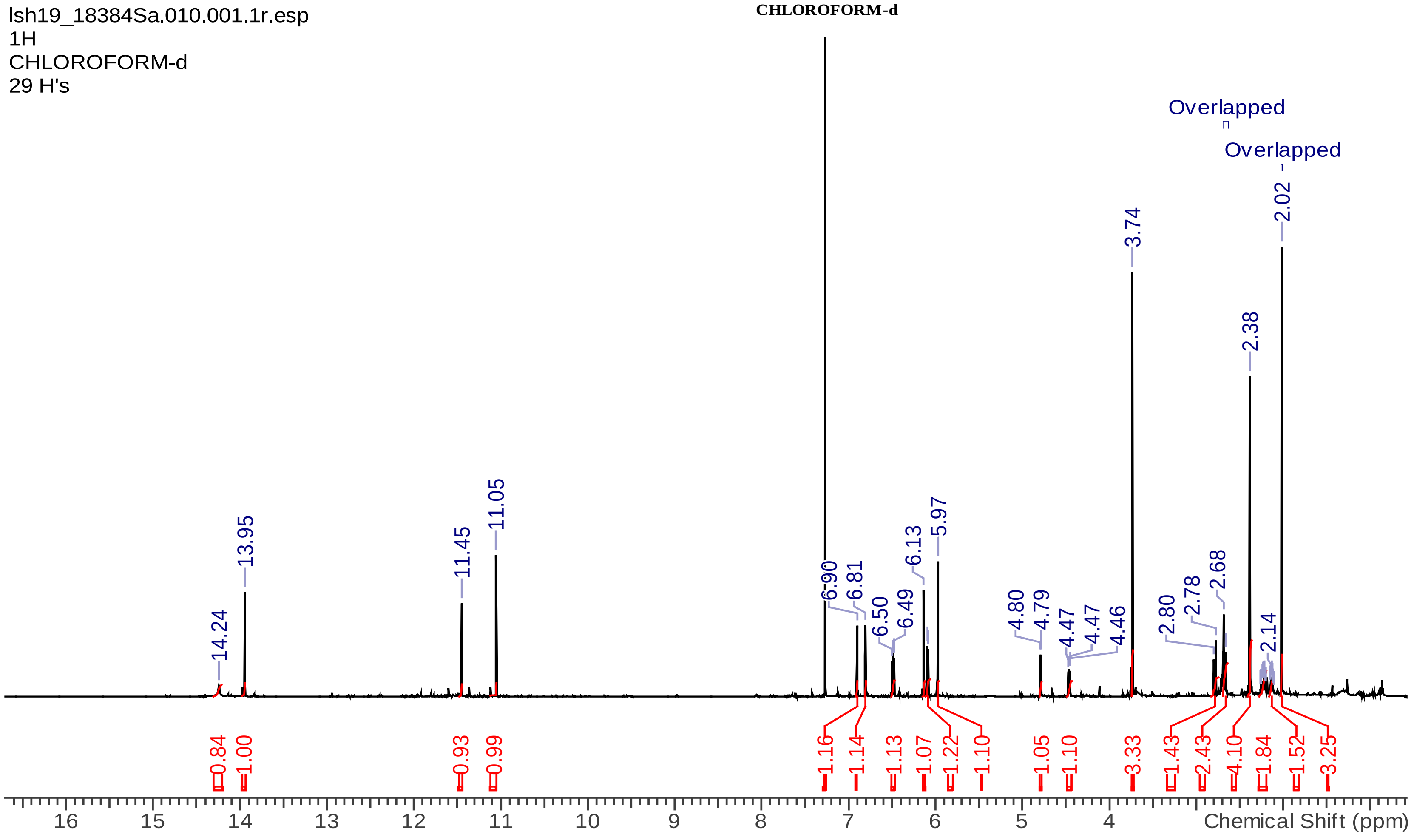

Figure S29. ^{1}H NMR spectrum (700 MHz, chloroform-*d*) of xanthoquinodin B13 (**5**)

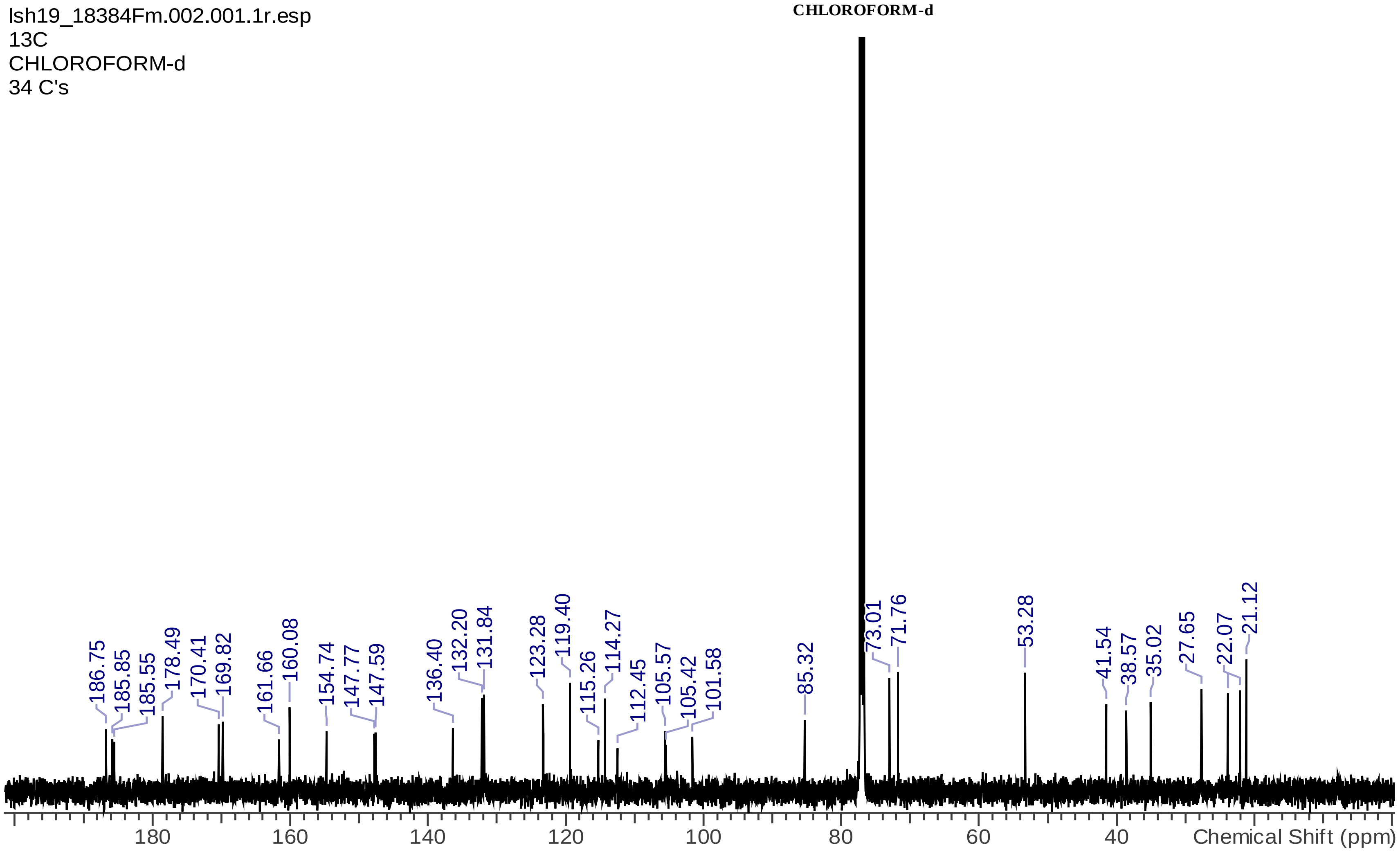

Figure S30. ^{13}C NMR spectrum (125 MHz, chloroform-*d*) of xanthoquinodin B13 (**5**)

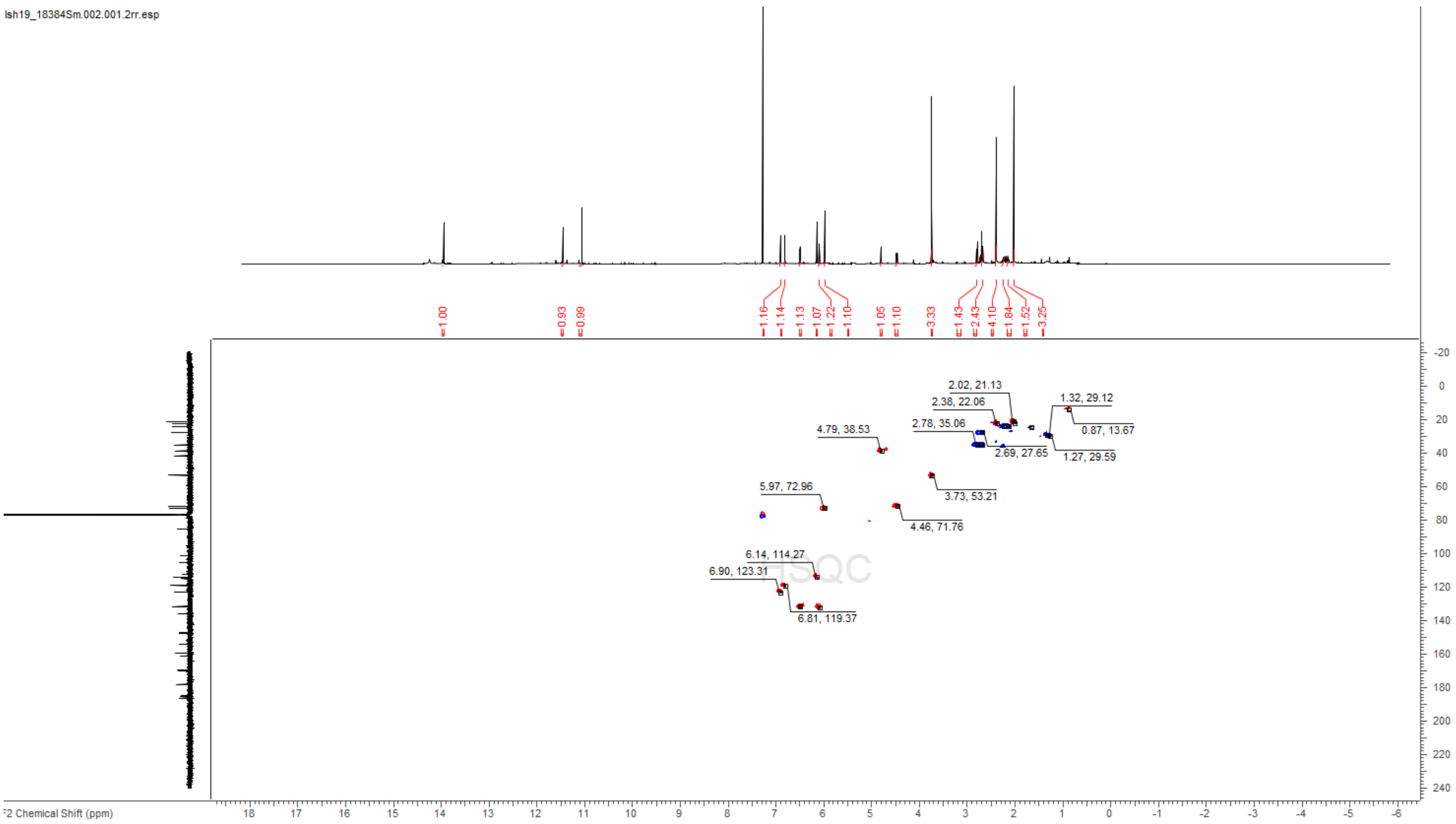

Figure S31. HSQC NMR spectrum (700 MHz, chloroform-*d*) of xanthoquinodin B13 (**5**)

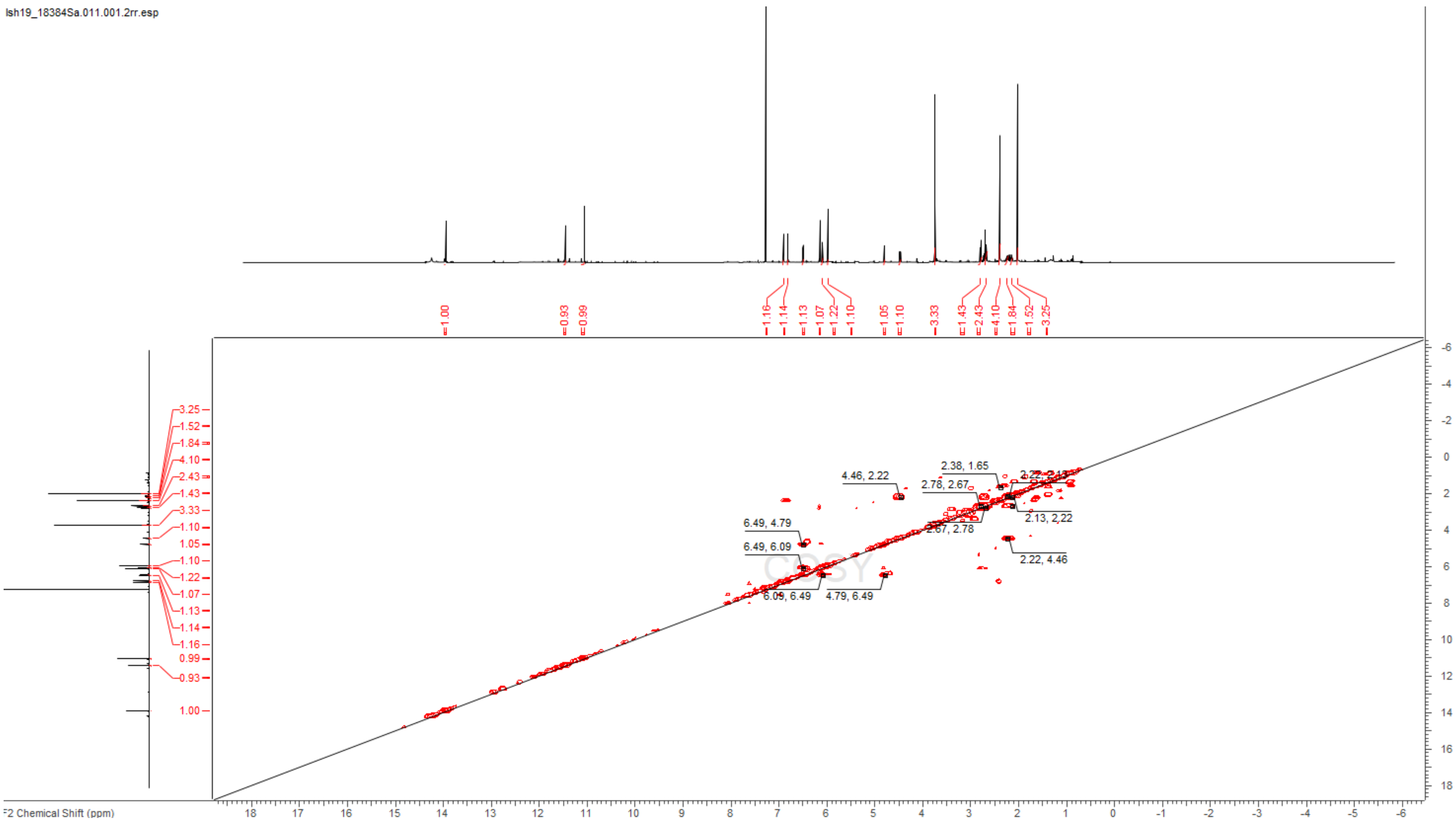

Figure S32. COSY NMR spectrum (700 MHz, chloroform-*d*) of xanthoquinodin B13 (**5**)

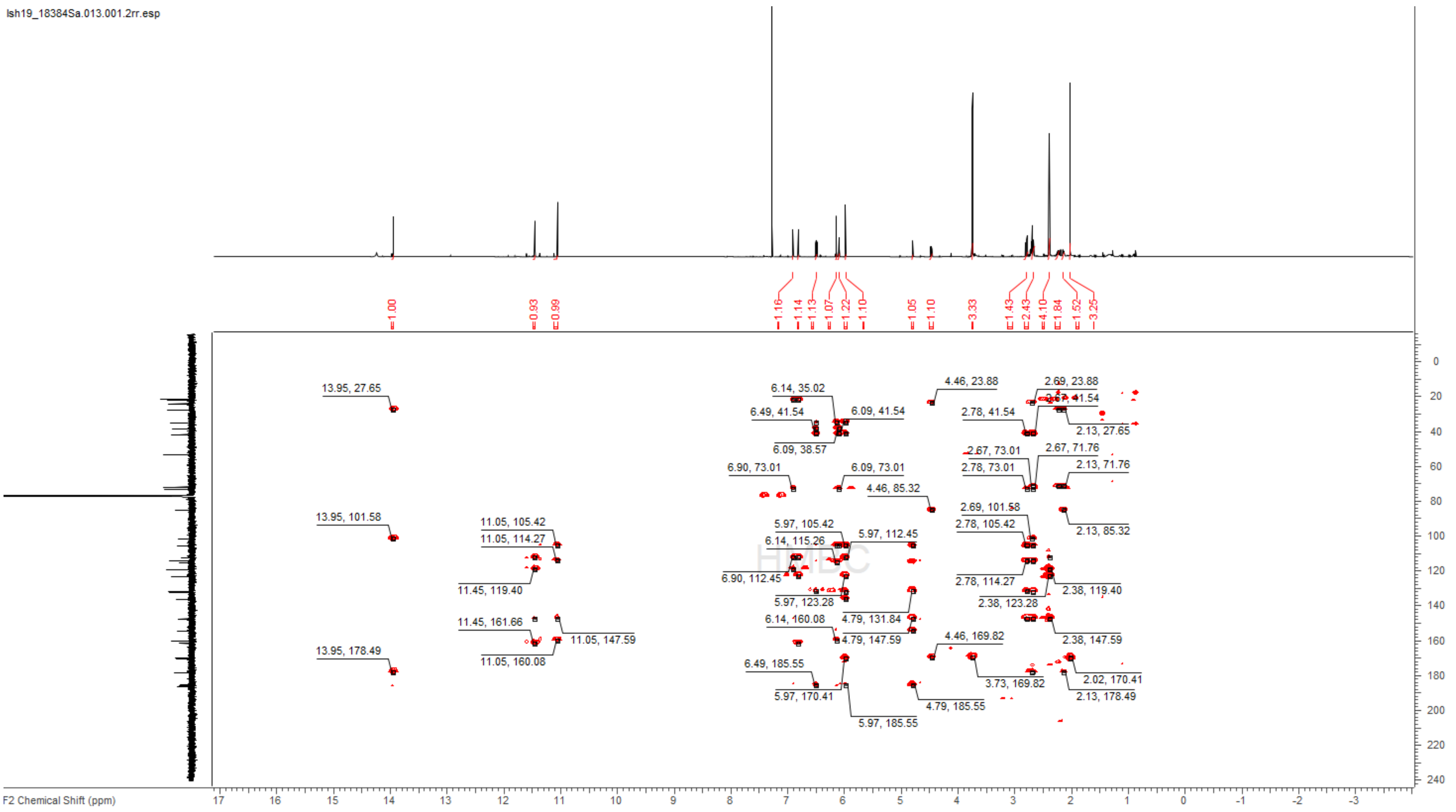

Figure S33. HMBC NMR spectrum (700 MHz, chloroform-*d*) of xanthoquinodin B13 (**5**)

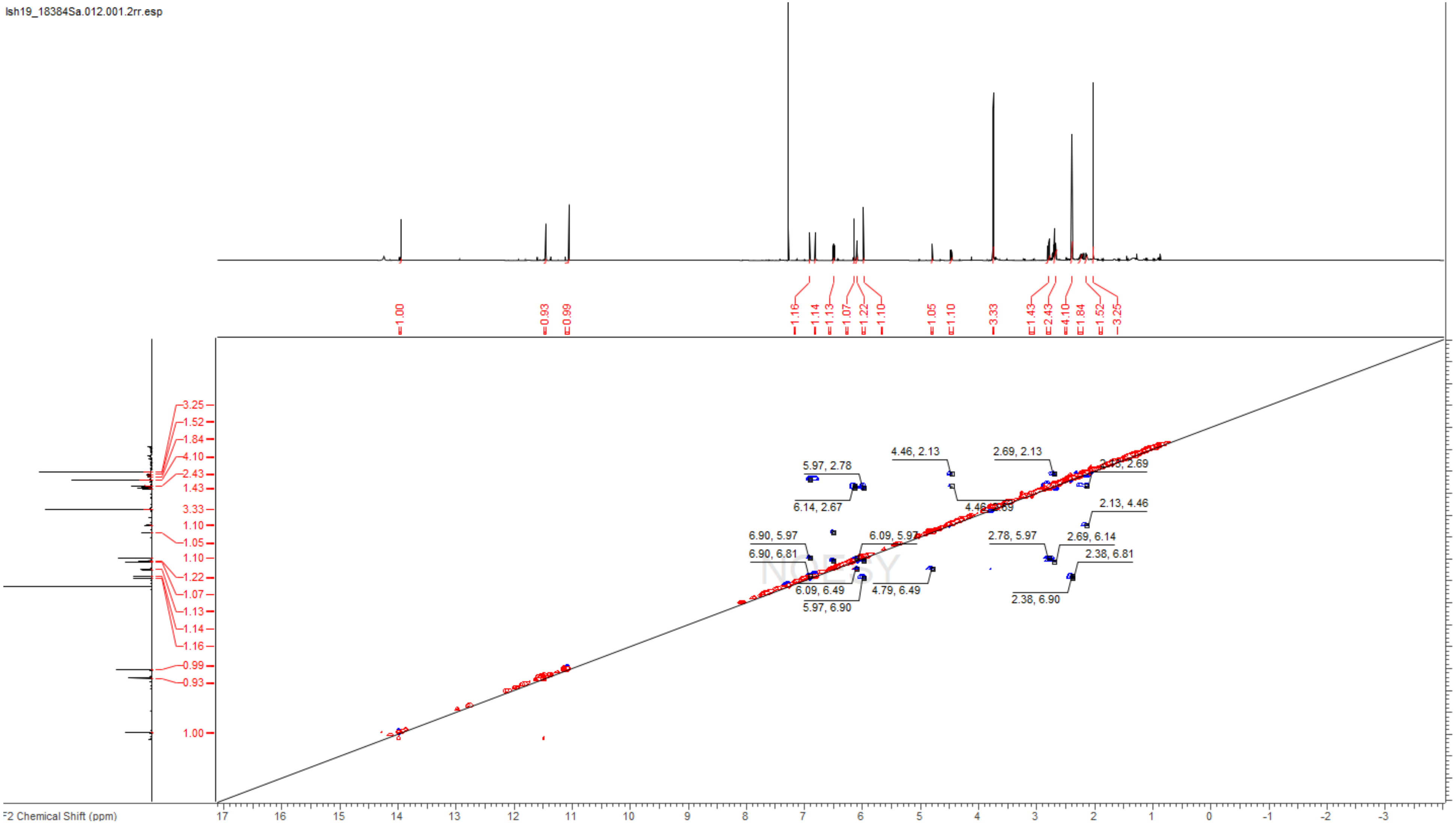

Figure S34. NOESY NMR spectrum (700 MHz, chloroform-*d*) of xanthoquinodin B13 (**5**)

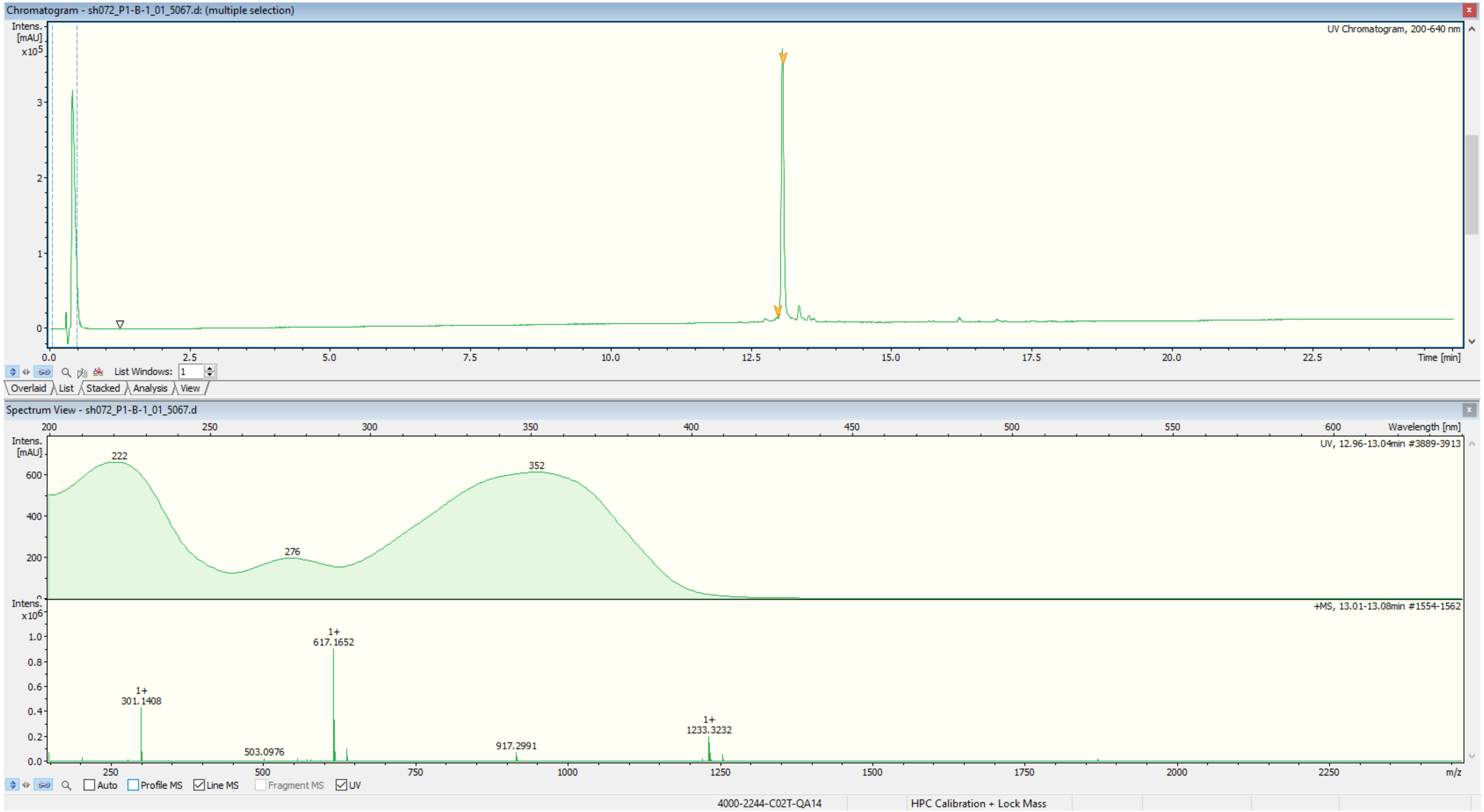

Figure S35. HRESIMS data of xanthoquinodin B13 (**5**)

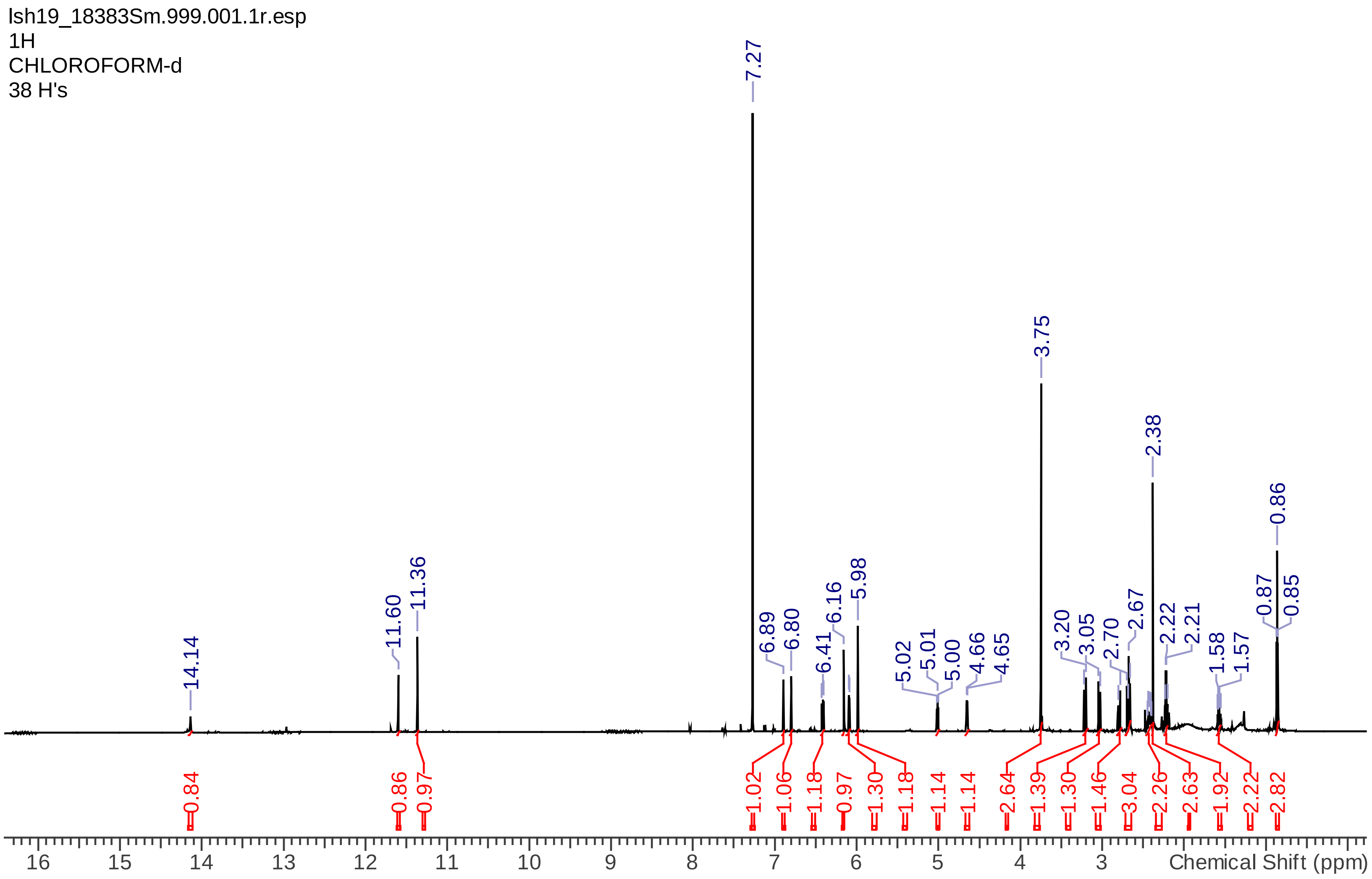

Figure S36. ^{1}H NMR spectrum (700 MHz, chloroform-*d*) of xanthoquinodin B14 (**6**)

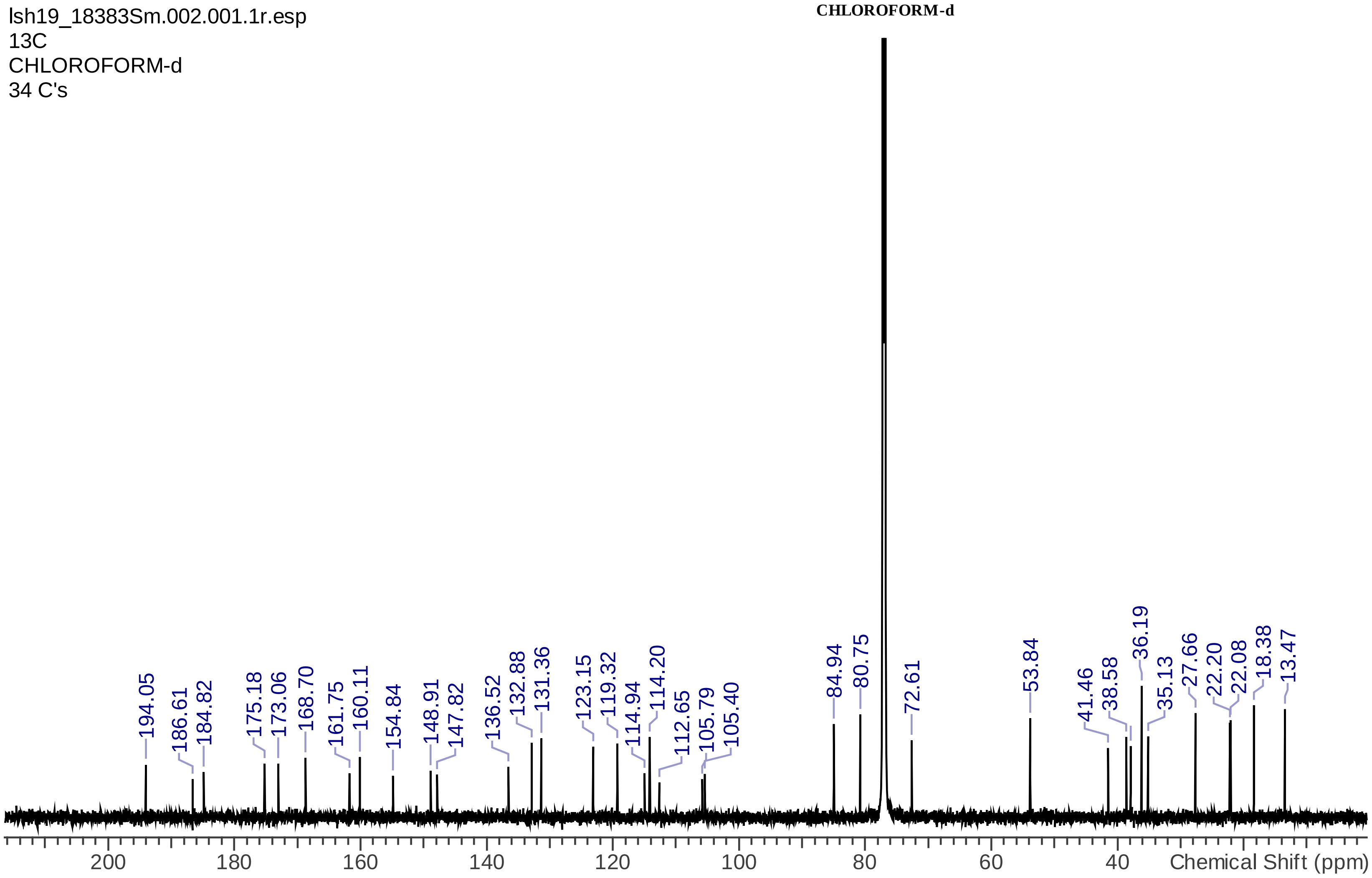

Figure S37. ^{13}C NMR spectrum (176 MHz, chloroform-*d*) of xanthoquinodin B14 (**6**)

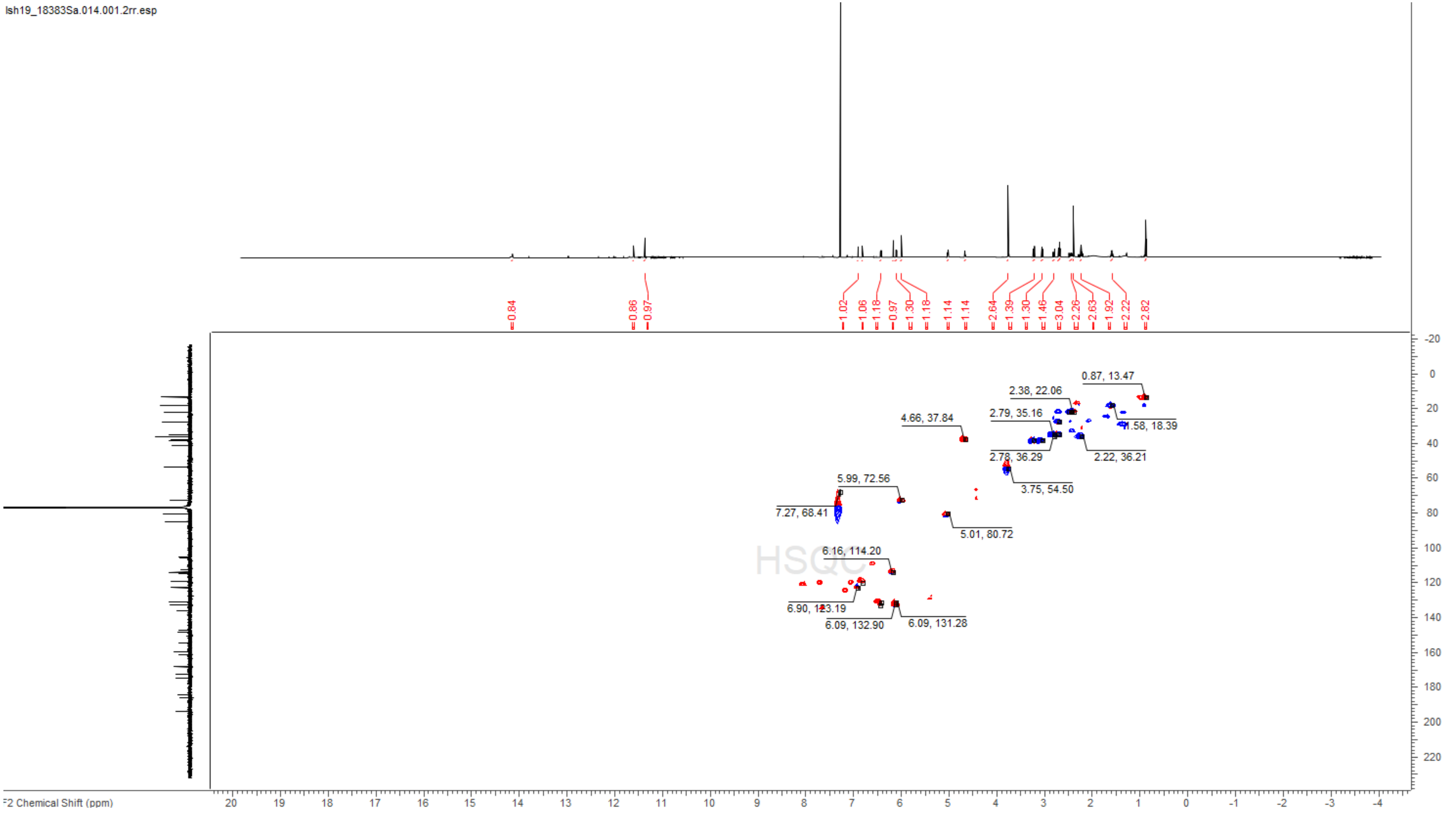

Figure S38. HSQC NMR spectrum (700 MHz, chloroform-*d*) of xanthoquinodin B14 (**6**)

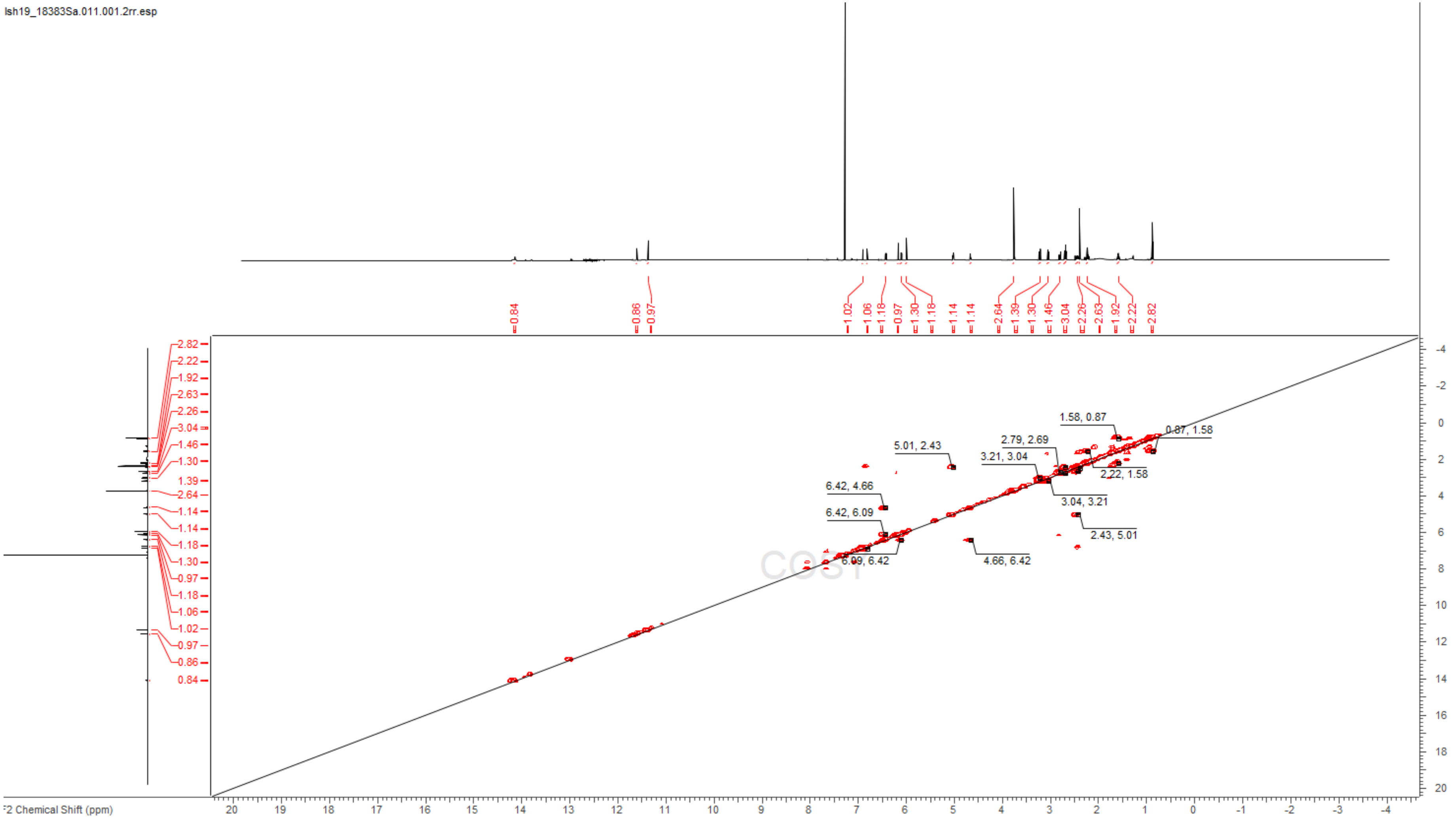

Figure S39. COSY NMR spectrum (700 MHz, chloroform-*d*) of xanthoquinodin B14 (**6**)

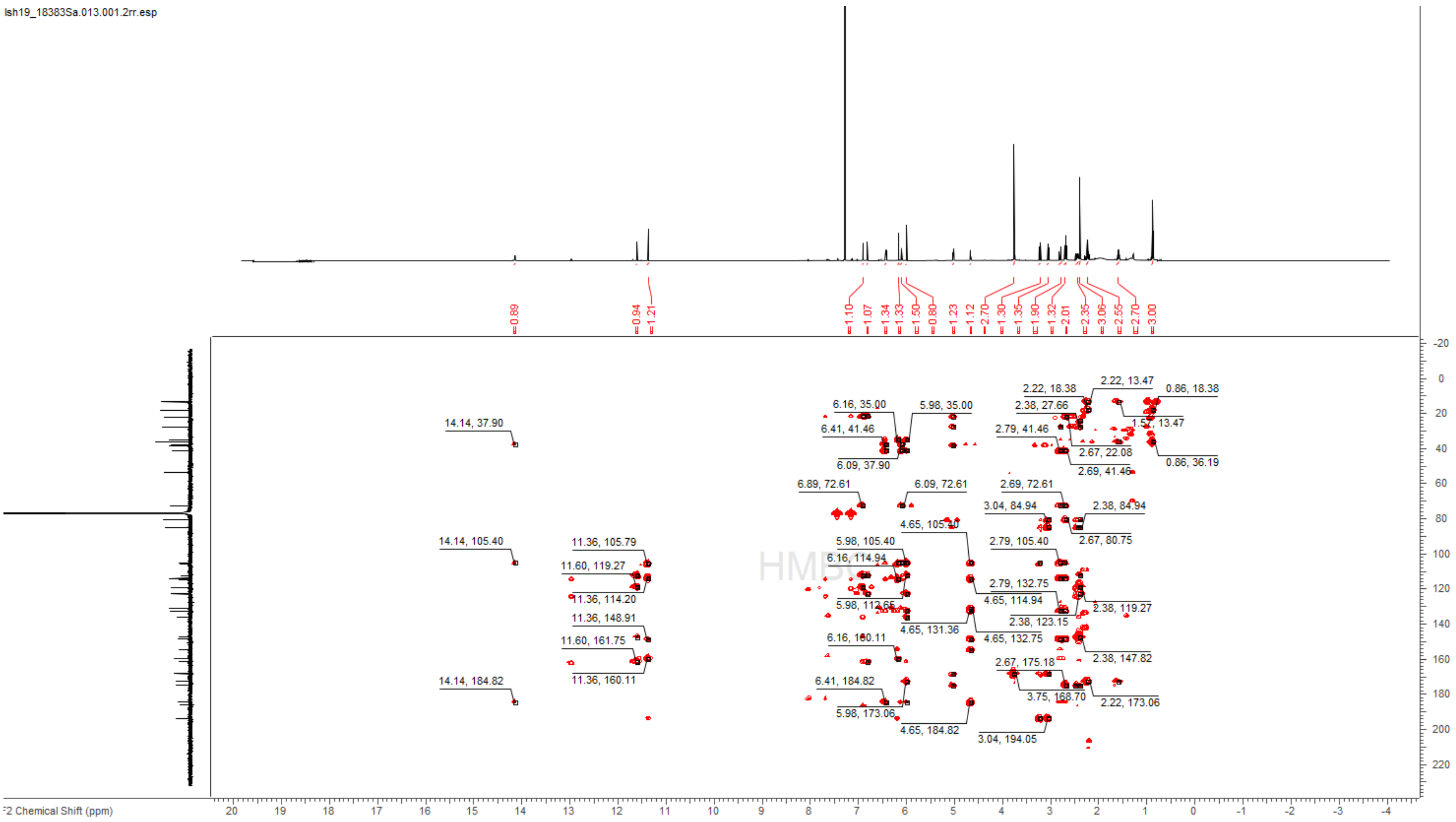

Figure S40. HMBC NMR spectrum (700 MHz, chloroform-*d*) of xanthoquinodin B14 (**6**)

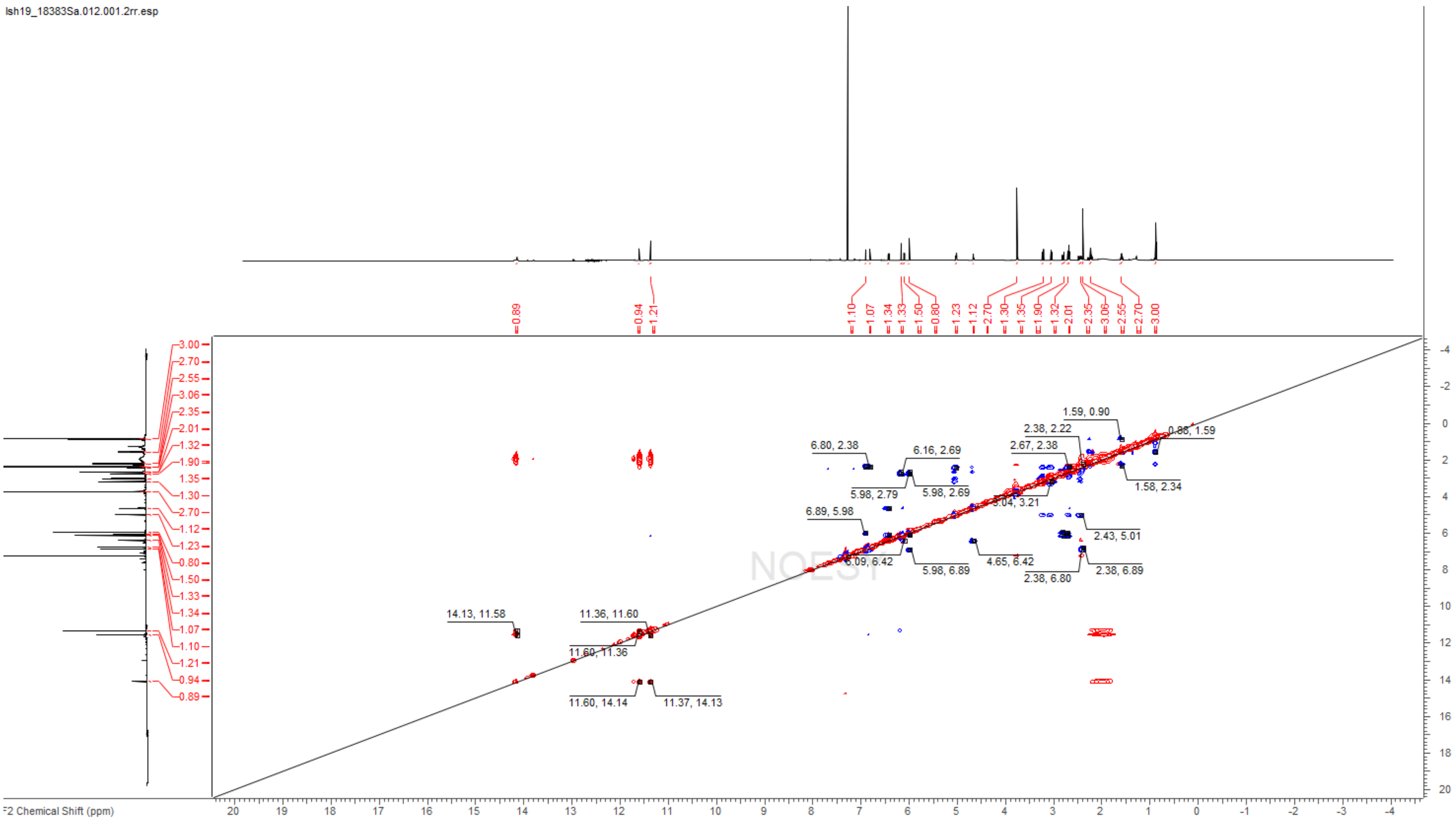

Figure S41. NOESY NMR spectrum (700 MHz, chloroform-*d*) of xanthoquinodin B14 (**6**)

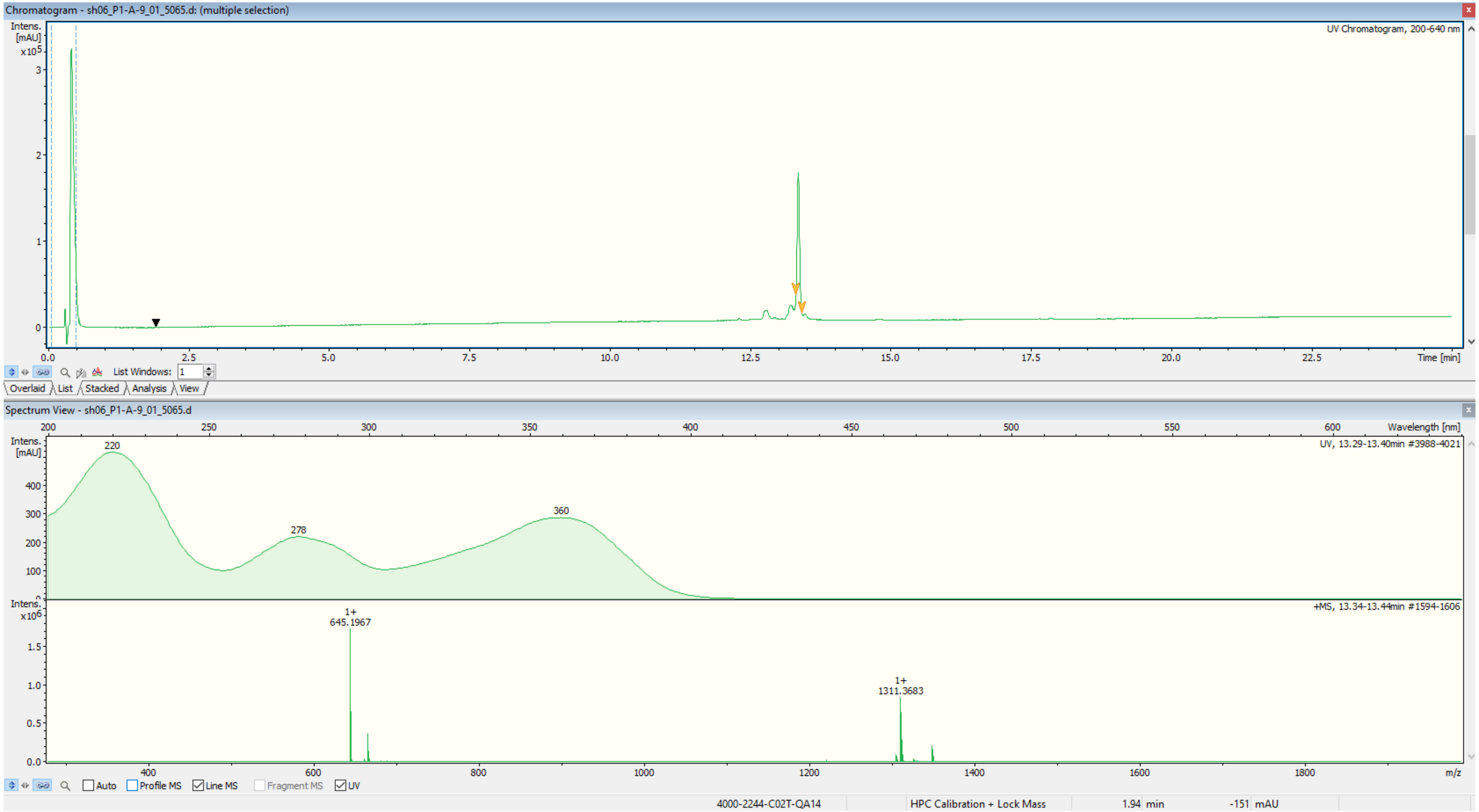

Figure S42. HRESIMS data of xanthoquinodin B14 (**6**)

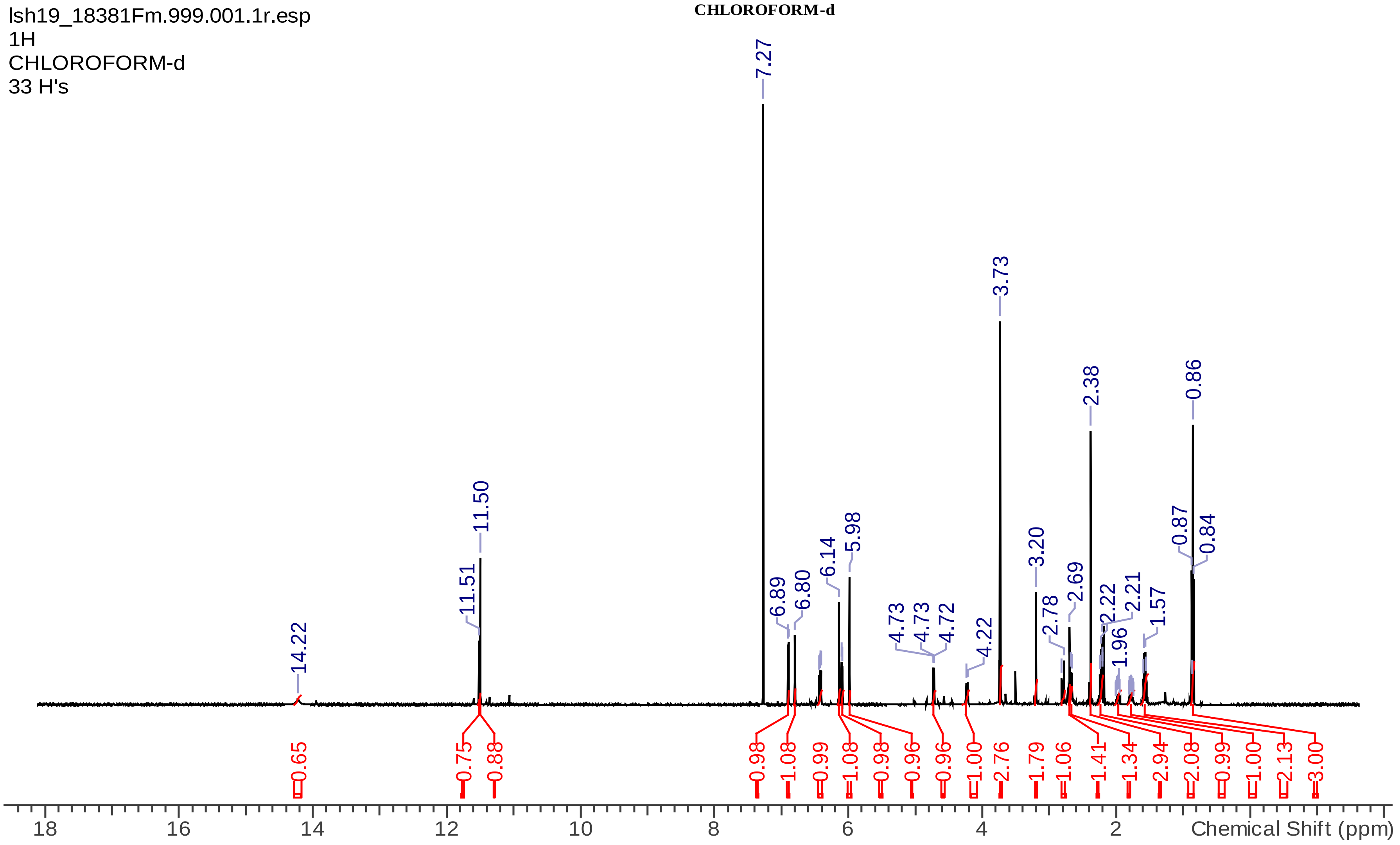

Figure S43. ^{1}H NMR spectrum (500 MHz, chloroform-*d*) of xanthoquinodin B15 (**7**)

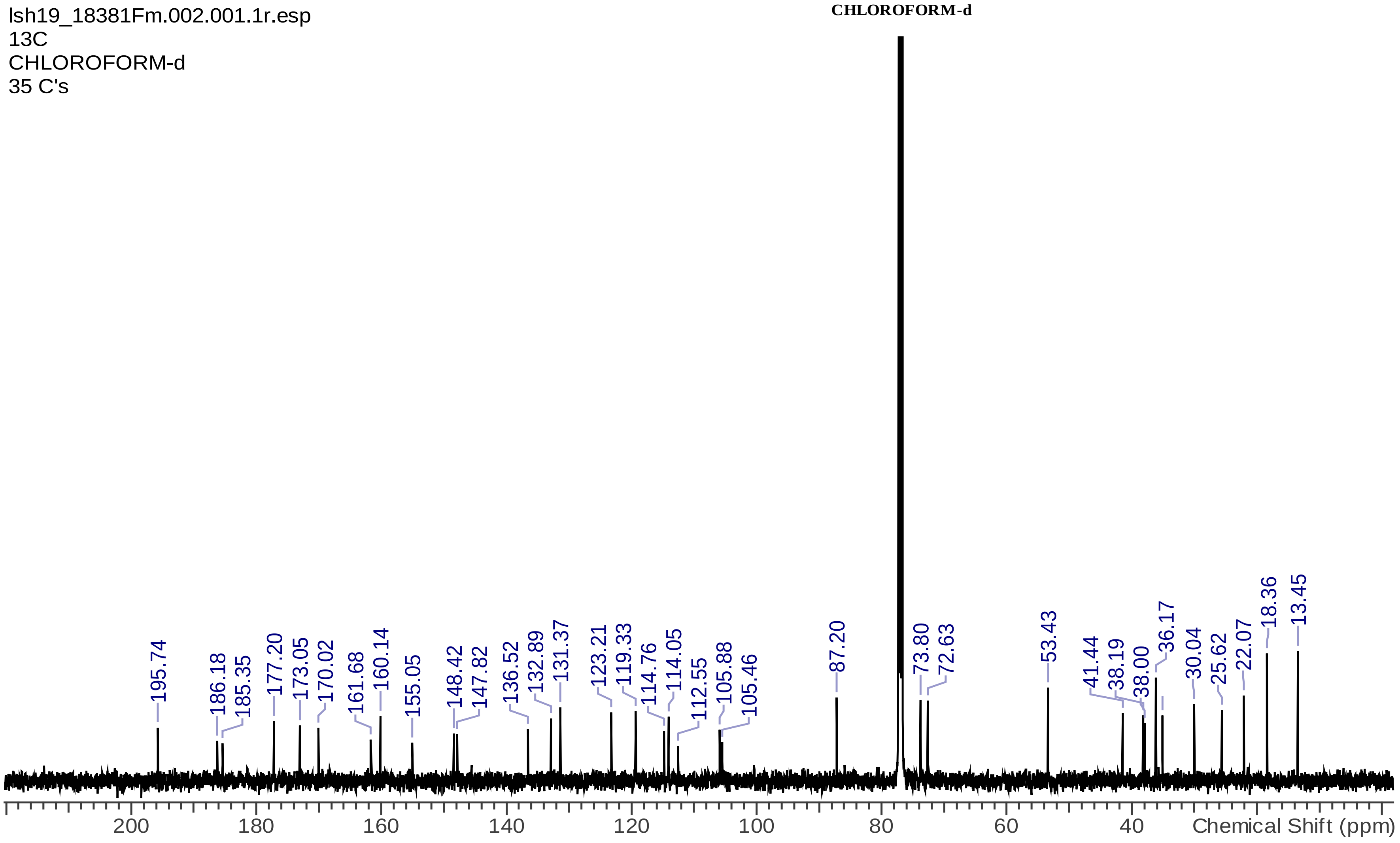

Figure S44. ^{13}C NMR spectrum (125 MHz, chloroform-*d*) of xanthoquinodin B15 (**7**)

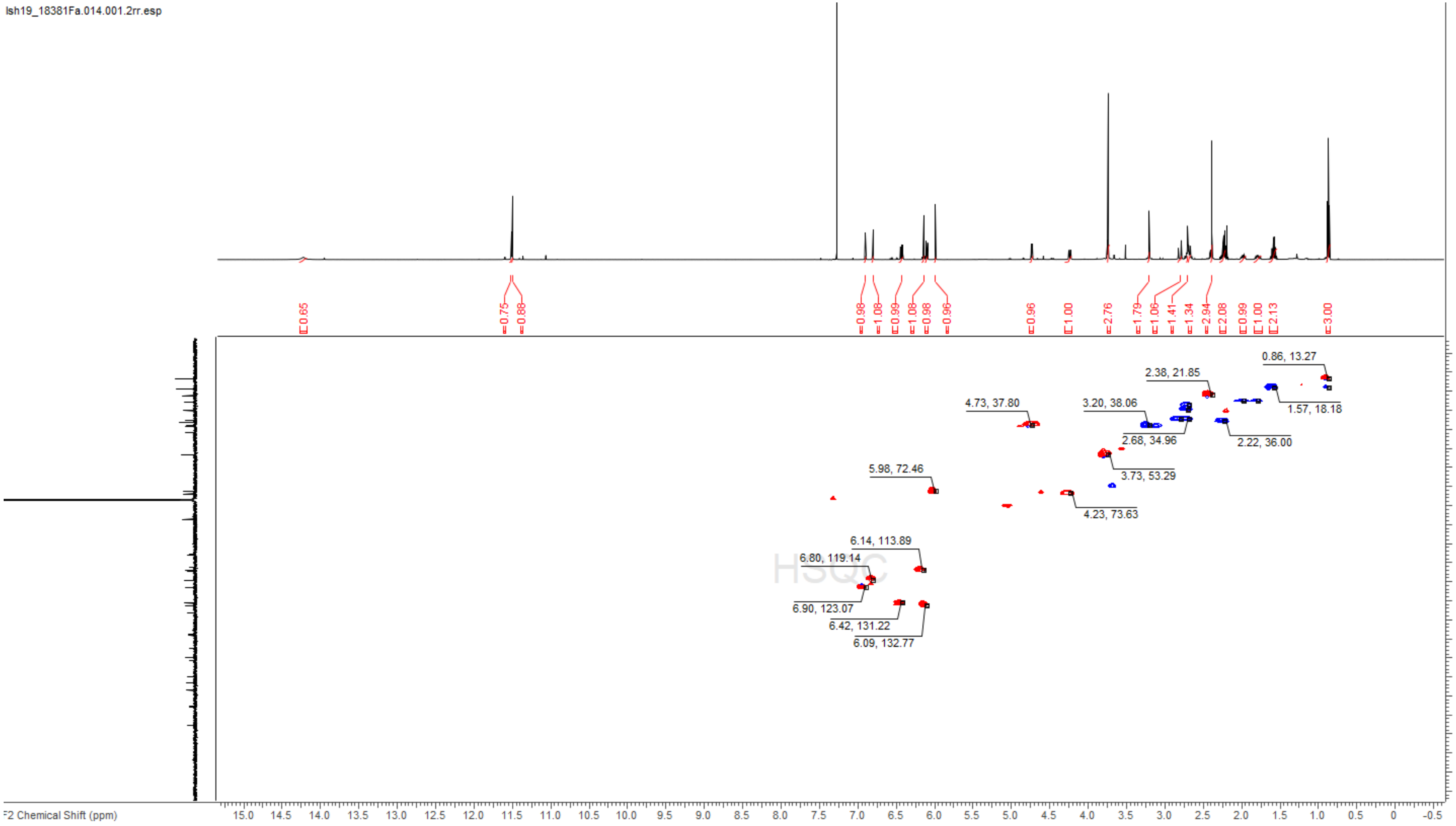

Figure S45. HSQC NMR spectrum (500 MHz, chloroform-*d*) of xanthoquinodin B15 (**7**)

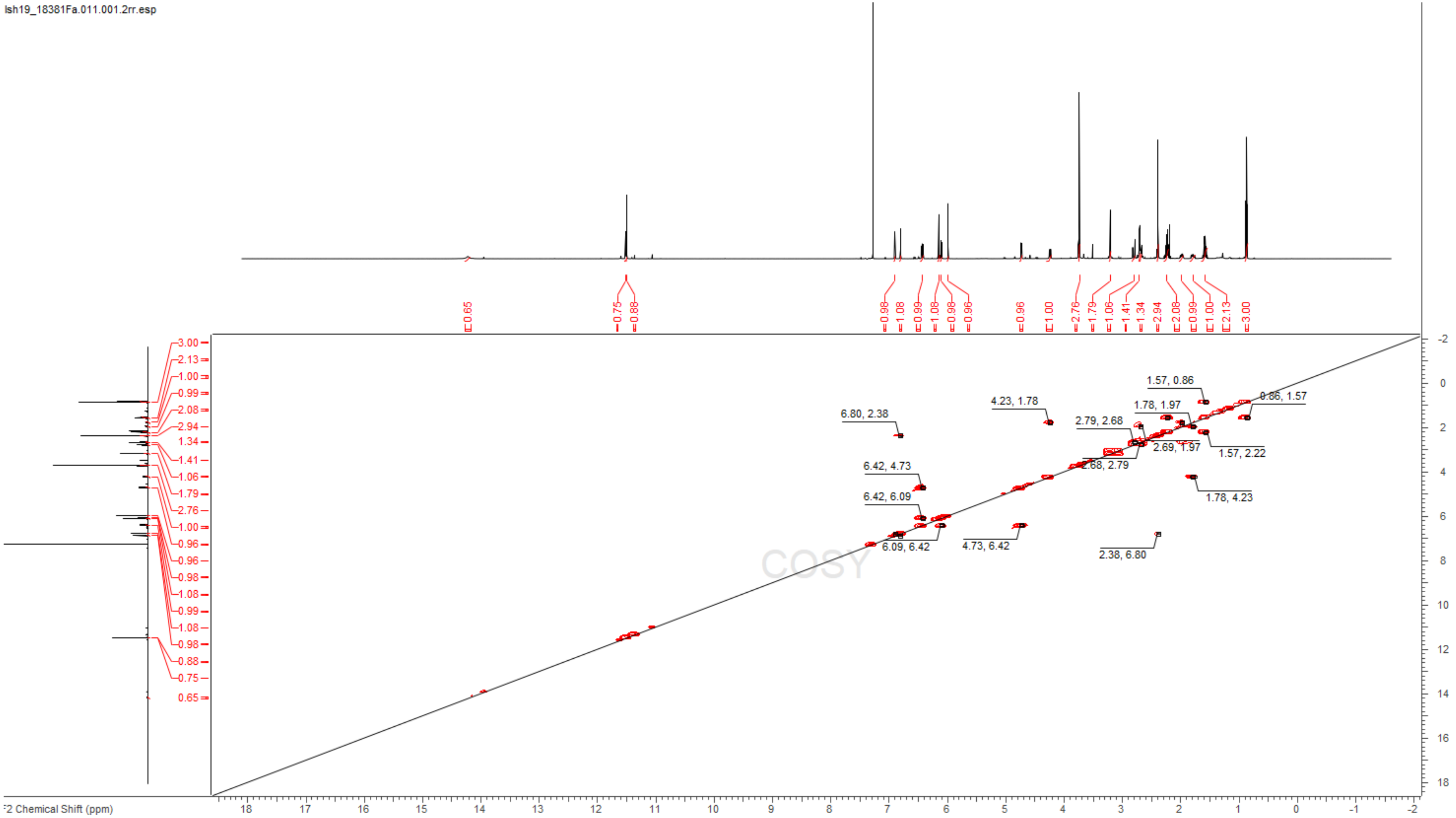

Figure S46. COSY NMR spectrum (500 MHz, chloroform-*d*) of xanthoquinodin B15 (**7**)

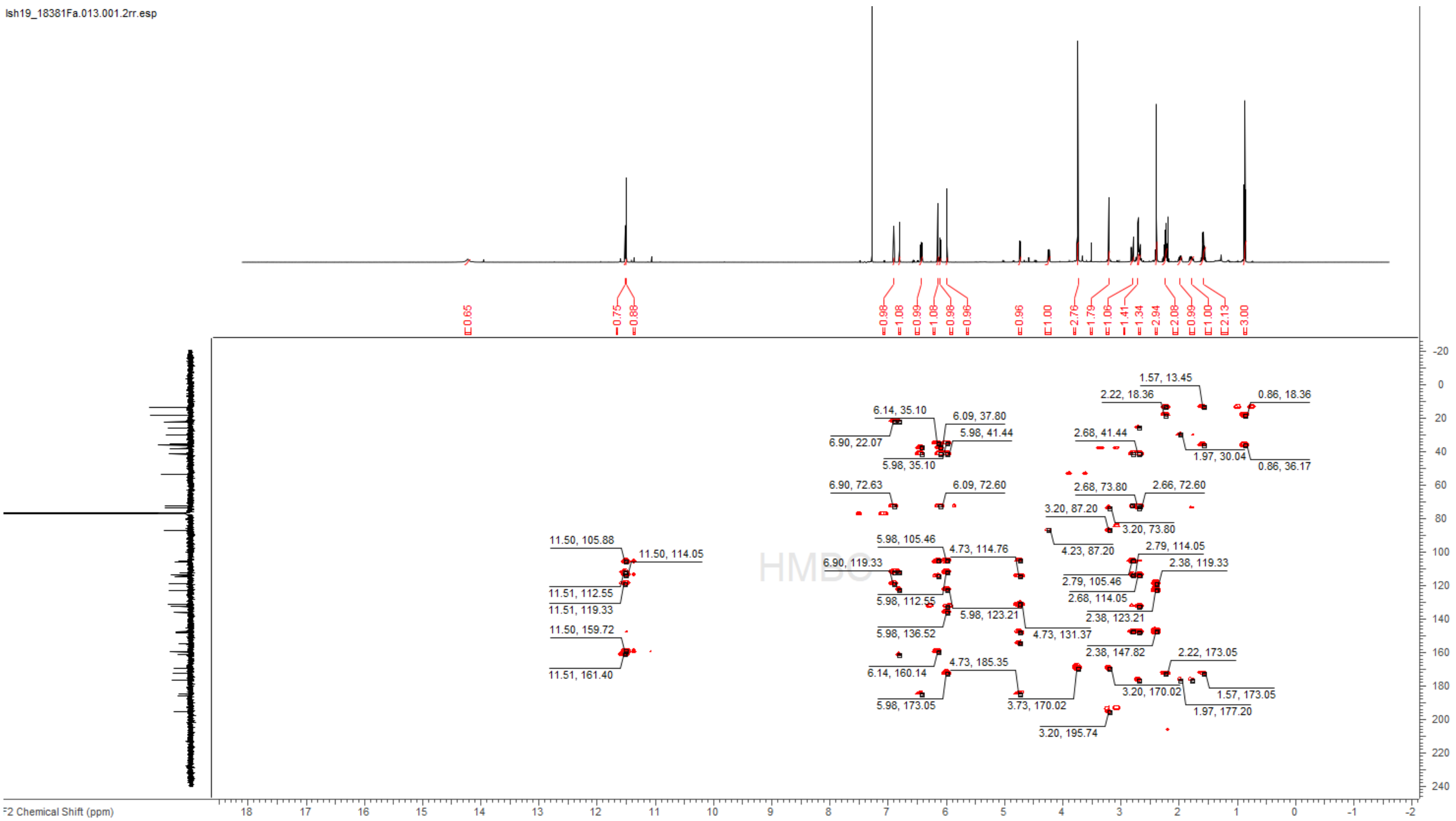

Figure S47. HMBC NMR spectrum (500 MHz, chloroform-*d*) of xanthoquinodin B15 (**7**)

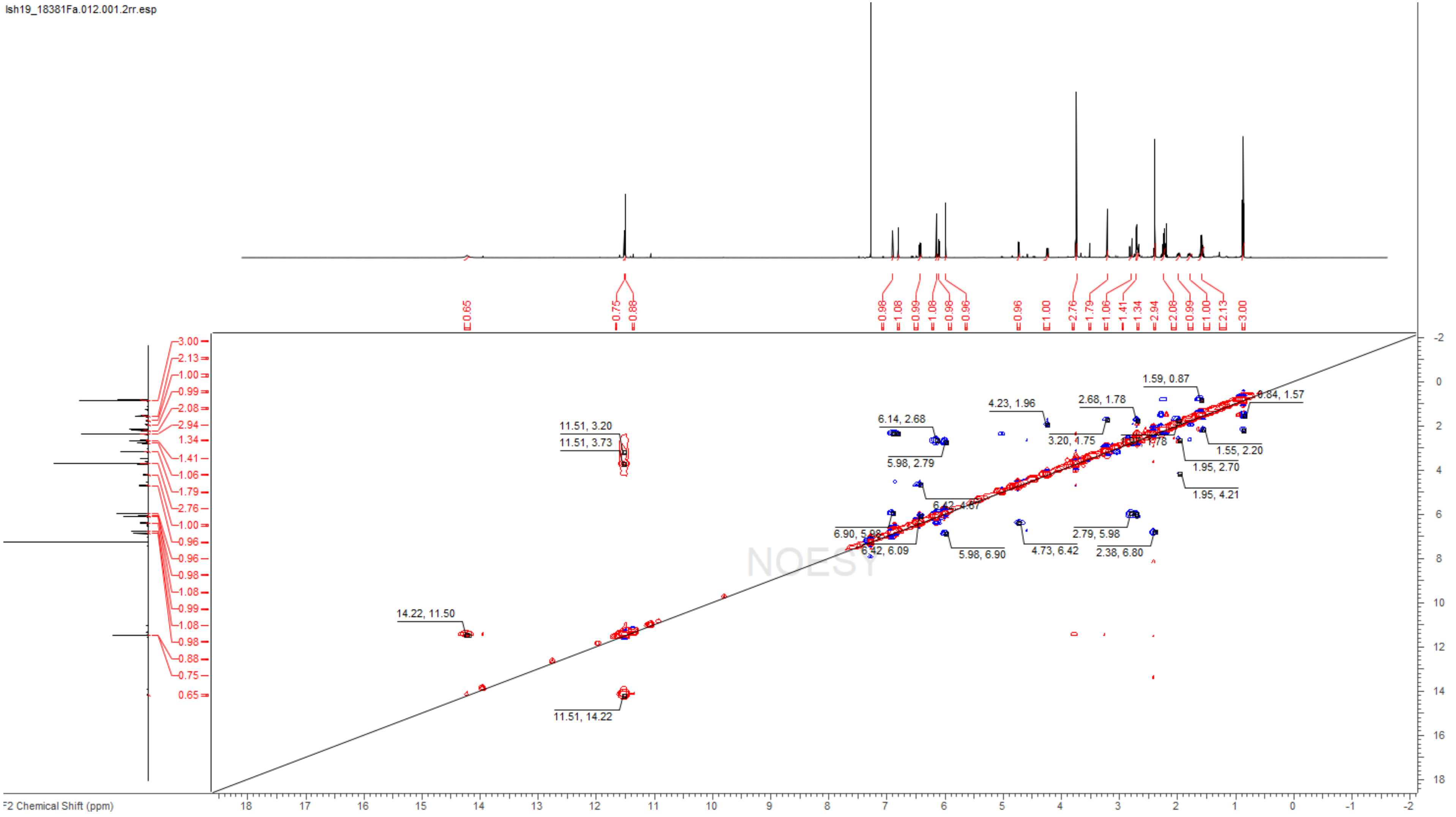

Figure S48. NOESY NMR spectrum (500 MHz, chloroform-*d*) of xanthoquinodin B15 (**7**)

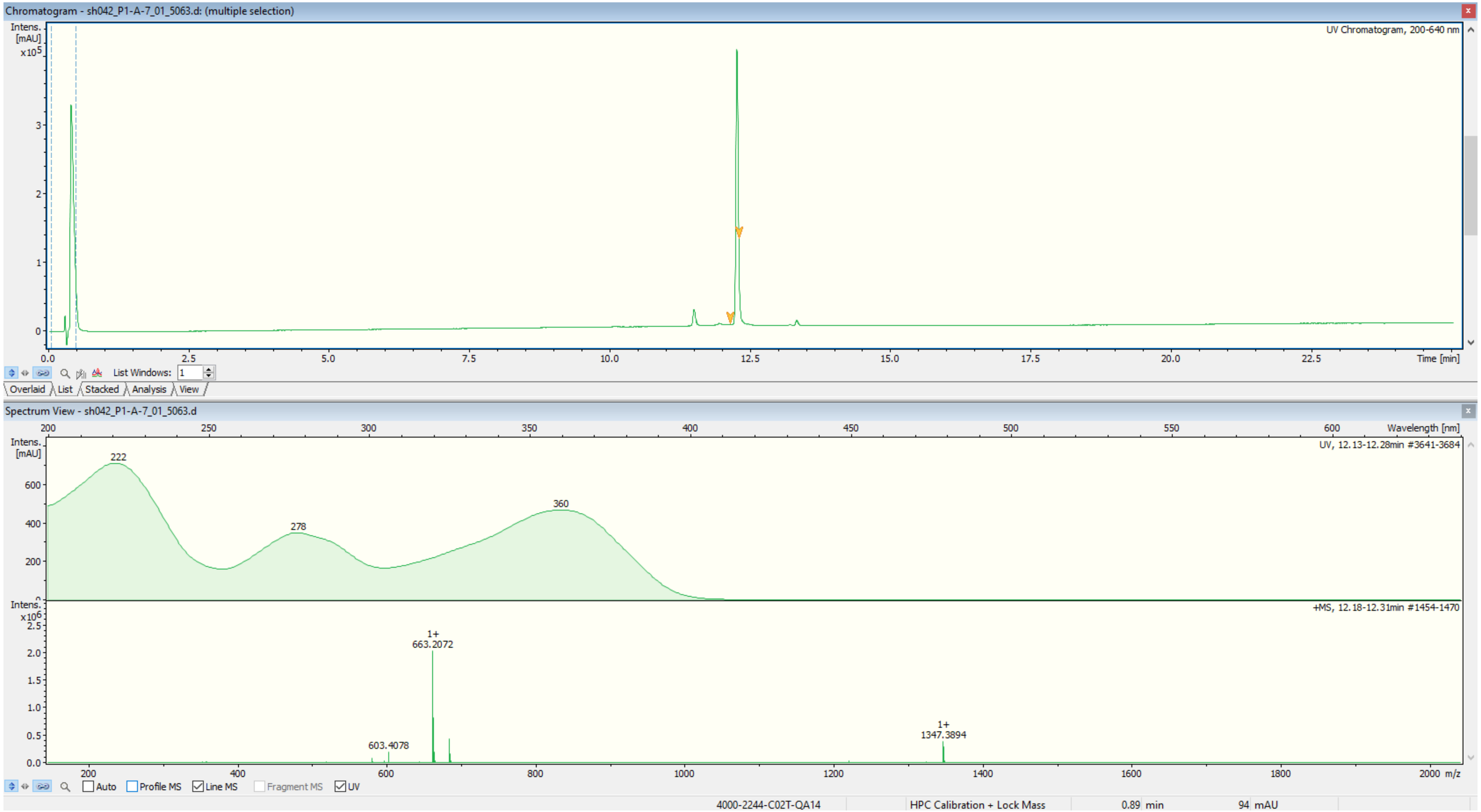

Figure S49. HRESIMS data of xanthoquinodin B15 (**7**)
